# Physical Origin of Temperature Induced Activation Energy Switching in Electrically Conductive Cement

**DOI:** 10.1002/advs.202517384

**Published:** 2025-11-21

**Authors:** Jiacheng Zhang, Xinyuan Ke, Andrew Heath, Richard J. Ball, Jiawei Xie, Jinchen Fan, Guisheng Li, Kevin Paine

**Affiliations:** ^1^ School of Materials and Chemistry University of Shanghai for Science and Technology Shanghai 200093 P. R. China; ^2^ Department of Architecture and Civil Engineering University of Bath Claverton Down Bath BA2 7AY UK

**Keywords:** activation energy switching, electrically conductive cement (CEMe), Meyer–Neldel rule (MNR), non‐Arrhenius behaviour, temperatures, thermodynamic modelling, tunnelling transmission

## Abstract

Electrically conductive cement (CEMe), enabled by percolative networks of conductive fillers, presents a promising future for multifunctional cementitious composites in next‐generation sustainable infrastructure. However, the mechanisms governing temperature‐dependent charge transport, particularly the temperature‐induced switching of Arrhenius activation energy, remain poorly understood. This study provides a rigorous investigation into the physical origin of variable activation energy behaviour in CEMe across different percolation regimes, a key factor for ensuring reliable multifunctional performance. It is demonstrated that activation energy switching arises from structural degradation within the biphasic conduction architecture: ionic transport through liquid‐filled connected pore network and electronic conduction via conductive carbon fibre network. In contrast, the intrinsic non‐Arrhenius behaviour of the pore solution and the Arrhenius behaviour of the carbon fibre have negligible influence on the overall activation energy switching behaviour. For the first time, Meyer–Neldel Rule (MNR) is observed in CEMe, attributed to the stable intrinsic conductivity (≈0.046 S m^−1^) of calcium silicate hydrate (C─S─H) gel across the temperature range of 5–90 °C and carbon fibre contents of 0–1.5 vol%. These findings advance the fundamental understanding of charge transport in CEMe and biphasic conducting materials systems, establishing a robust scientific basis for designing intelligent, multifunctional materials that adapt to dynamic environments.

## Graphical abstract

The temperature‐induced Arrhenius activation energy switching phenomenon of electrical conduction in electrically conductive cement originates from structural degradation within the biphasic ionic‐electronic conduction architecture and shows percolation‐governed characteristics: pore network opening dominates the low‐percolation regime with downward switch of activation energy whilst fibrous network degradation prevails in the high‐percolation regime with activation energy switching upward.

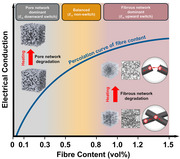

## 1 Introduction

### 1.1 Next‐Generation Building with Electrically Conductive Cement

The next‐generation building strategy calls for stringent demands that the concrete infrastructures should possess minimized environmental impact whilst maintaining the necessary performance characteristics.^[^ ^1^, ^2^ ^]^ To that end, contemporary concrete structures are advancing toward biomimetic self‐sufficiency which allows for long‐term energy self‐circulation and intelligent structure self‐maintenance. This can be achieved by integrating cementitious composites with multifunctionalities, such as energy harvesting,^[^ ^3^, ^4^ ^]^ self‐powering,^[^ ^5^, ^6^, ^7^, ^8^ ^]^ self‐sensing,^[^ ^9^, ^10^, ^11^ ^]^ self‐healing,^[^ ^12^, ^13^ ^]^ self‐cleaning,^[^ ^14^, ^15^ ^]^ and self‐heating.^[^ ^16^, ^17^ ^]^ These innovative technologies offer superior advantages over off‐the‐shelf devices owing to their natural compatibility with the main concrete structure, cost‐effectiveness, and most importantly, the tailored functionality. Currently, most multifunctional cementitious composites exploit “electrically coupled” properties such as piezo‐resistivity and thermo‐electricity. These can be considered a subclass of electrically conductive cement that relies on charge transport kinetics for mass transfer and energy conversion. Electrically conductive cement is constructed via the incorporation of functional fillers with distinctive conductive features (i.e., graphene, carbon fibre, defective titania, etc.). Through the percolative process of the functional fillers, electrically conductive cement exhibits a spectrum with interconvertible conducting natures: insulative, ionic, and electronic, where different functionalities of interest can be fulfilled. For example, the self‐sensing concrete (with respect to stress/strain) requires the conduction to be strongly electronic for a stable signal transmission,^[^ ^18^, ^19^ ^]^ whilst energy storage concrete requires a strongly ionic conduction for efficient charge transport but preventing direct short circuit between the electrodes.^[^ ^20^, ^21^ ^]^ Despite extensive research on electrically conductive cement, from proof‐of‐concept studies utilizing innovative functional fillers for the attainment of advanced physicochemical properties,^[^ ^22^ ^]^ to modelling approaches attempting to unveil the origin of multifunctionality,^[^ ^23^, ^24^, ^25^ ^]^ the underlying mechanisms governing the temperature dependence of its electrical conduction behaviours remain insufficiently understood. Changes in temperature almost cover every aspect of anthropogenic activity and occur frequently in electrically conductive cement with respect to direct environmental influence on performance,^[^ ^26^, ^27^, ^28^ ^]^ joule heating effect utilization,^[^ ^29^, ^30^ ^]^ limited heat‐dissipation problem from long‐term servicing,^[^ ^31^ ^]^ etc. Because charge transport is thermodynamically driven, temperature directly influences the electrical conduction and, ultimately, the functional performance. Understanding the temperature‐dependent charge transport mechanism is therefore fundamental for innovative and sustainable development of electrically conductive cement.

The activation energy, *E_a_*, quantifies temperature‐induced variations in many physico‐chemical processes, such as chemical reaction,^[^ ^32^ ^]^ nucleation,^[^ ^33^ ^]^ diffusion,^[^ ^34^ ^]^ and electrical conduction.^[^ ^35^ ^]^ It is often obtained from the slope of the linearized Arrhenius plot, where the logarithm of the quantity of a process is linearly correlated to the inverse of the temperature. When subject to temperature variation which is wide enough, charge transport in hardened cement, which is manifested as electrical conduction properties, can have activation energy switching (or variable activation energy) behaviour showing different activation energies at different temperature ranges.^[^ ^36^ ^]^ For hardened cement as a single ionic‐conducting system through pore solution,^[^ ^37^ ^]^ the activation energy decreases above 50 °C^[^ ^38^ ^]^ and increases below −8.7 °C.^[^ ^39^ ^]^ The latter effect was postulated to result from a significant reduction in the volume of liquid‐form pore solution due to freezing,^[^ ^40^ ^]^ whereas the origin of the former remains undefined. Moreover, when subject to narrower temperature variations, activation energy stays constant, but being inversely dependent on moisture content^[^ ^41^, ^42^ ^]^ and positively dependent on pore structure constriction.^[^ ^43^ ^]^ Apart from ionically conductive systems like hardened cement, activation energy switching was found to exist in many electronically conductive systems such as semiconductors and composite polymers.^[^ ^44^, ^45^, ^46^ ^]^

Despite being a powerful approach for empirical practices, there have been no studies quantitively and experimentally uncovering the physical origin of temperature‐induced activation energy switching behaviour of the electrical conduction process in hardened cement. Also, existing studies have focused on the activation energy of conduction in hardened plain or blended cements as a single ionic‐conducting system. There has been no study formally defining the physical concept of the Arrhenius activation energy *E_a_* of the bulk conduction process in electrically conductive cement, which behaves as a biphasic conducting system. The physical origin of the activation energy switching in electrically conductive cement has yet to be uncovered. Moreover, the percolation law governing electrically conductive cement, which is key to designing multifunctional cementitious composites, remains underexplored with respect to temperature‐dependent conductivity and standardised activation energy values.

### 1.2 Definition of *E_a_* in Electrically Conductive Cement

The electrical conduction process in electrically conductive cement is biphasic, consisting of both ionic and electronic conduction. The ionic conduction is produced by the migration of free ions (i.e., mainly K^+^, Na^+^, Ca^2+^, OH^−^, and SO_4_^2−^) through continuously connected liquid filled pore network,^[^ ^47^, ^48^ ^]^ whilst the electronic conduction is relatively discontinuous and disordered which is produced by the transport of electrons/holes through filler body, filler–filler direct contacting points,^[^ ^49^, ^50^ ^]^ and the filler–filler tunnelling gaps.^[^ ^51^, ^52^, ^53^ ^]^ Based on this rationale, out of simplicity and conciseness, we define the physical concept of Arrhenius activation energy *E_a_* of bulk conduction in electrically conductive cement: it is an effective (or apparent) value by a weighted sum of the activation energies of ionic and electronic conduction:

(1) $$E_{a}=a\left(bE_{a,ionic}+cE_{a,electronic}\right)$$

where *E*_*a*,*ionic*_ and *E*_*a*,*electronic*_ are activation energies of ionic and electronic conduction respectively, and *a*, *b*, and *c* are the coefficients. The *E_a_* quantifies the effective energy barrier that must be overcome for charge carriers to generate a conduction process (i.e., a long‐range drift of charge carriers in the direction of the electric field). Based on the physical definition of electrical conduction, ionic conduction is controlled by the intrinsic conductivity of the pore solution (an intensive property) and the architecture of the pore network (a dimension‐ and morphology‐dependent property), whereas electronic conduction is controlled by the intrinsic conductivity of the filler and the architecture of the filler network. CEMe exhibits compositional and microstructural heterogeneity, and changes in temperature can trigger complex physico‐chemical degradation processes, hence compromising the electrical conduction.^[^ ^54^ ^]^ For instance, the chemical stability of solid phases (i.e., C─S─H gel, portlandite, ettringite, etc.) varies at different temperatures, whose decomposition can influence pore structure and, upon dissolution, can affect the intrinsic conductivity of pore solution.^[^ ^55^ ^]^ The pore solution, as a multi‐species aqueous electrolyte, can undergo solvent precipitation/re‐dissolution (i.e., particularly portlandite) under changing temperature, in turn having a thermodynamically controlled intrinsic conductivity.^[^ ^56^ ^]^ The filler is more chemically inert than hardened cement (particularly carbon‐based conductors), however, its thermally activated electrons/holes transport and volume straining can induce alterations in both intrinsic conductivity and architecture of the filler network.^[^ ^49^, ^57^ ^]^ Therefore, understanding the physical origin of activation energy switching in CEMe, whether governed by intrinsic material properties, microstructural architecture, or their interplay, is essential for understanding the physico‐chemical stability of the biphasic conduction pathways under varying temperature and, consequently, its electrically coupled multifunctionality.

Combining experiments, thermodynamic modelling, and mathematical analysis, this study explores the temperature‐dependent charge transport in electrically conductive cement (CEMe) by uncovering the physical origin of activation energy switching behaviours. CEMes were fabricated with 0–1.5 vol% polyacrylonitrile (PAN) based chopped carbon fibres, and thermal cycling between 5 and 90 °C was applied under near water saturated conditions. Electrochemical impedance spectroscopy (EIS) was used to reveal the Arrhenius and non‐Arrhenius behaviours across the percolation regimes in CEMe. The activation energy switching in ionic conduction was linked to temperature‐induced changes in pore solution conductivity and pore network geometry, predicted and analyzed combining chemical thermodynamic modelling of the cementitious matrix and mercury intrusion porosimetry (MIP) microstructure analysis. The hysteresis of activation energy and conductivity between heating and cooling was experimentally reproduced and validated based on National Institute of Standards and Technology (NIST), Moragues et al., and Taylor's data.^[^ ^58^, ^59^, ^60^, ^61^ ^]^ The physical origin of activation energy switching in the electronic conduction process was then interpretated by experimentation and mathematical analysis (i.e., formation factor approach and percolation theory) on temperature‐induced changes in carbon fibre intrinsic conductivity and the architecture of the fibrous network. The Meyer–Neldel Rule (MNR), an important empirical law for validating Arrhenian behaviour and guiding the design/tuning of conductive/semiconductive materials, was evaluated within the 0–1.5 vol% fibres percolation regime and analyzed in relation to the physico‐chemical properties of the solid and liquid conductive components in CEMe. Ultimately, scientific findings were reformulated into engineering tool references for a straightforward evaluation of charge transport performance, materials degradation, and environmental susceptibility in CEMe under temperature‐varying conditions. Scientific findings and engineering remarks from this study advance the understanding on the thermally activated charge transport physics in CEMe and biphasic conducting materials systems, opening new design and durability control over pathways for multifunctional cementitious composites in next‐generation infrastructure.

## 2 Results and Discussion

### 2.1 Identification of Impedance Characteristics

**Figure 1:**
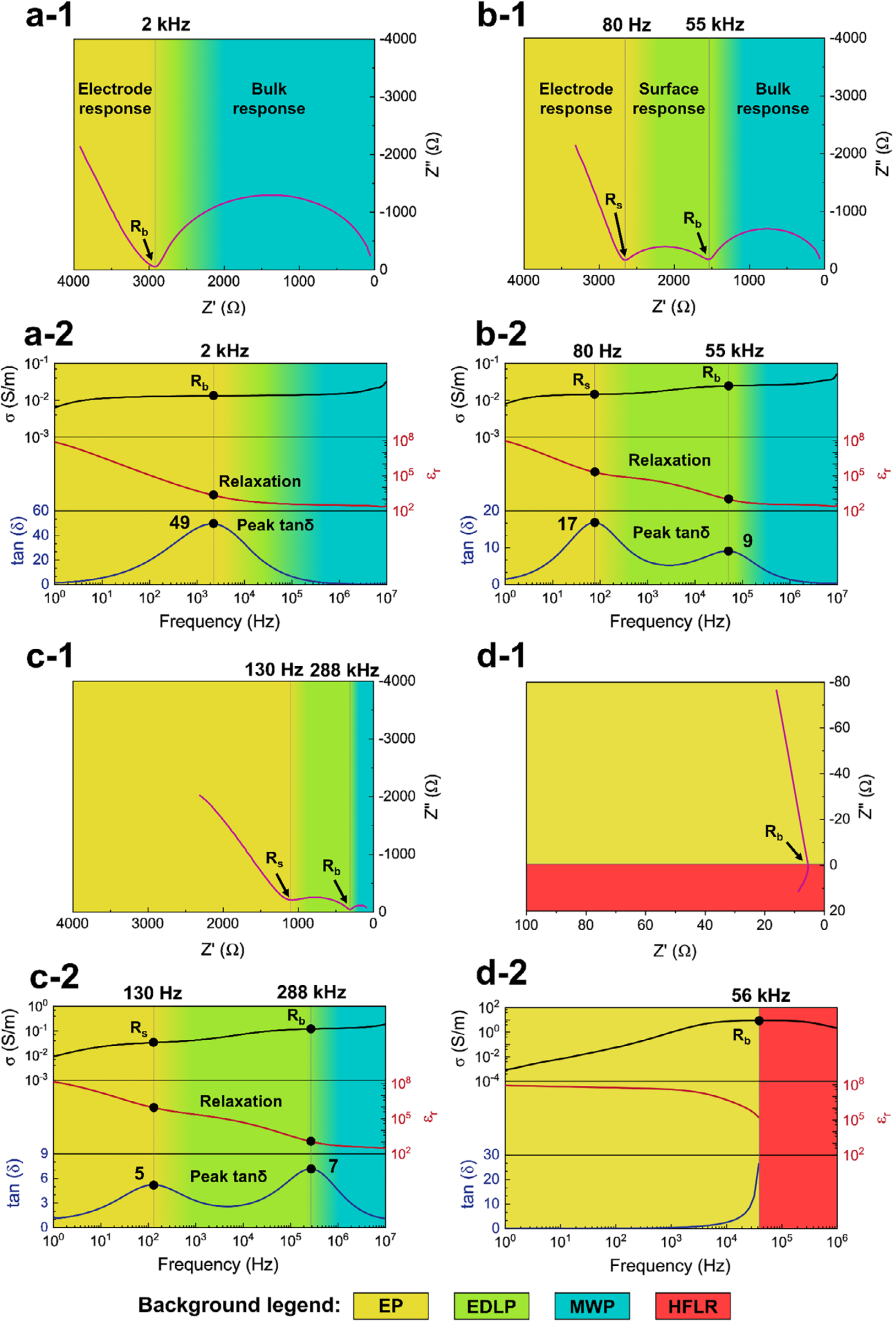
Representative impedance responses of PM and CEMes incorporating different dosages of PAN‐based chopped carbon fibres, and simulated pore solution at pristine conditions. a‐1) Nyquist plot of PM. a‐2) Bode plot of PM. b‐1) Nyquist plot of CEMe with relatively lower fibre contents 0.1, 0.3, 0.5, and 0.8 vol% which are termed as S1, S3, S5, and S8, respectively (i.e., S1 was presented as an example). b‐2) Bode plot of CEMe with relatively lower fibre contents (i.e., S1 was presented as an example). c‐1) Nyquist plot of CEMe with relatively higher fibre contents 1, 1.2, and 1.5 vol% which are termed as S10, S12, S15 (i.e., S15 was presented as an example). c‐2) Bode plot of CEMe with relatively higher fibre contents (i.e., S15 was presented as an example). d‐1) Nyquist plot of simulated pore solution (termed as SPS). d‐2) Bode plot of simulated pore solution.

The impedance response of CEMes with different fibre contents, as well as the simulated pore solution, is presented both in Nyquist and Bode plots (**Figure** **1**), for identifying the characteristic conduction and dielectric parameters, which were further used for extracting Arrhenius behaviours. Four dielectric response regions were identified at pristine conditions, which are electrode polarization (EP, in yellow background), bulk electric double layer polarization (EDLP, in green background), bulk Maxwell–Wagner interfacial polarization (MWP, in blue background), and a high frequency limit region (HFLR, in red background). The impedance response of hardened cement is a collective work of conduction and polarization processes operative on the solid–liquid interfaces between the reaction products of cement and the pore solution, and in the bulk of the pore solution.^[^ ^48^, ^62^ ^]^ Upon the inclusion of conductive carbon fibre, the electronic conduction via fibrous network was introduced, and the polarization process was enhanced.^[^ ^63^, ^64^, ^65^ ^]^

**Figure 2:**
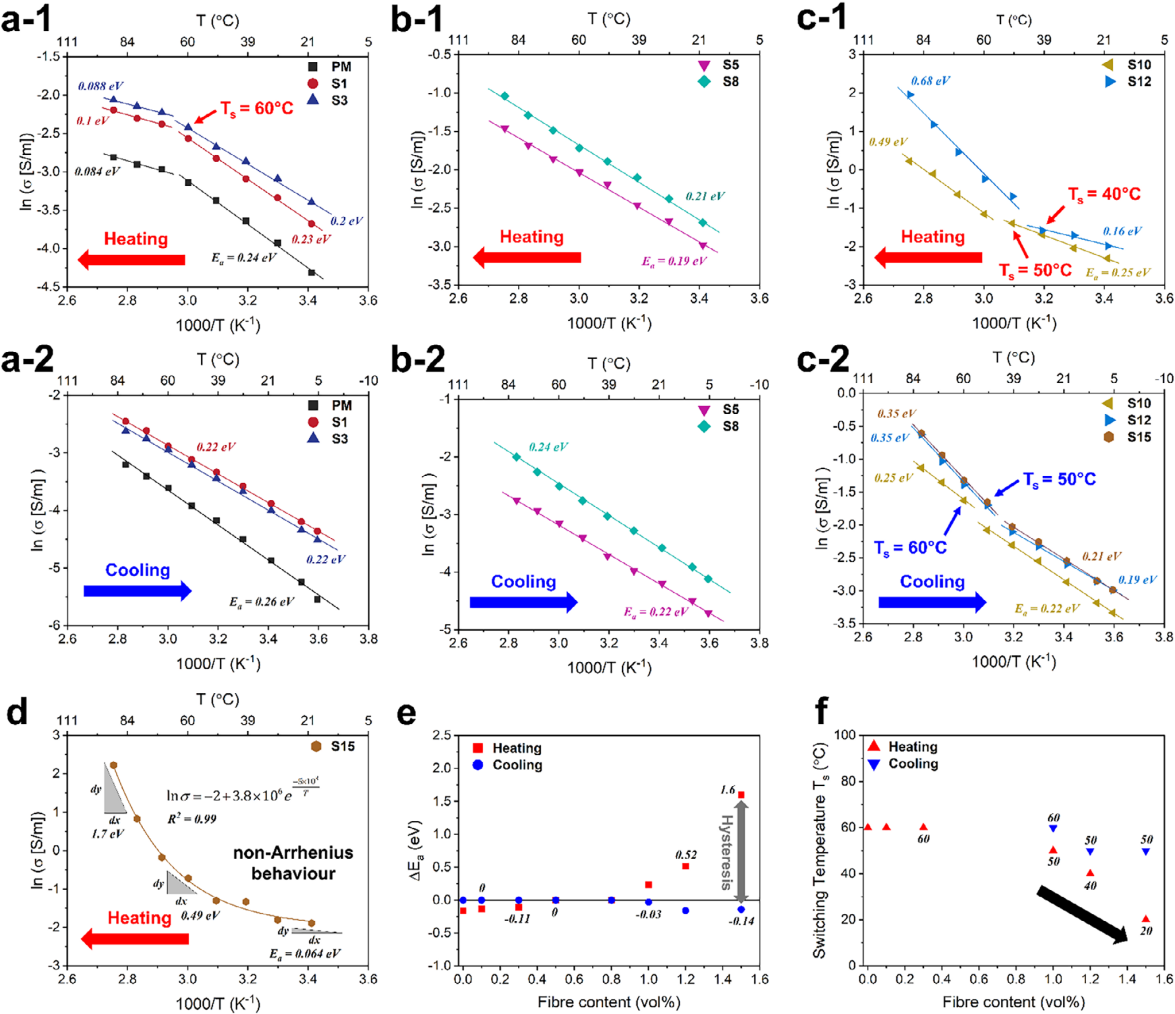
Arrhenius and non‐Arrhenius behaviours of bulk conductivity of CEMe presenting different activation energy switching behaviours at different fibre contents. a‐1) PM, S1, S3 during heating. a‐2) PM, S1, S3 during cooling. b‐1) S5 and S8 during heating. b‐2) S5 and S8 during cooling. c‐1) S10 and S12 during heating. c‐2) S10 and S12 during cooling. d) S15 during heating. e) Δ*E_a_* (i.e., Δ*E_a_*  =  *E*_*a*,*post*_  −  *E*_*a*,*pre*_) during the thermal cycle to quantify the *E_a_* switching degree for CEMes with different fibre contents and the hysteresis of Δ*E_a_* between heating and cooling, *E*_*a*,*pre*_ and *E*_*a*,*post*_ are the activation energies before and after switching, respectively. f) switching temperatures *T*_s_ for PM and CEMes during thermal cycle (non‐switching behaviour was not plotted).

In Figure 1a‐1, the Nyquist plot of plain mortar (PM) consists of an electrode response “spur” at frequencies 1 Hz–2 kHz and a bulk response arc at frequencies 2 kHz–10 MHz. The low‐frequency electrode response originates from the charge transfer resistance and double‐layer capacitance at the electrode/pore solution interface which was a feature for two‐point EIS measurement.^[^ ^62^ ^]^ The high‐frequency bulk arc stems from the weak surface conduction and EDLP process at pore walls/pore solution interfaces, as well as the strong bulk ionic conduction and MWP in the bulk electrolytic environment.^[^ ^66^, ^67^ ^]^ These polarization processes were superimposed with each other at frequency boundaries due to the overlapped operative frequencies, as well as the prevalence of solid–liquid interfaces in the cement matrix.^[^ ^68^ ^]^ Such a superposition, due to covering a few frequencies, was indicated via the gradient transition of the colored backgrounds. The gradient transition of green to light blue indicates the diminishing of bulk EDLP and the prevalence of bulk MWP as the frequency increases. The bulk EDLP occurs only within a narrow frequency range embedded in the bulk MWP arc, due to the low surface charge concentration at the pore wall/solution interface, which limits surface conduction.^[^ ^69^, ^70^ ^]^ The bulk resistance, *R_b_*, can be obtained at the cusp point of electrode response “spur” and the bulk response arc at 2 kHz, where the EP diminished and the bulk EDLP prevails.^[^ ^48^ ^]^ (Section S8.1, Supporting Information). The Bode plot of PM (Figure 1a‐2) was aligned underneath its Nyquist plot (Figure 1a‐1), where the *R_b_* and polarization processes were highlighted consistently. The peak value of tanδ was obtained at 2 kHz, being the same as that of *R_b_*. This is due to the relaxation of electric double layers at the electrode/solution interface, yielding a peak tanδ of 49 and maximum energy dissipation contributing to bulk ionic conduction.^[^ ^71^ ^]^

The incorporation of carbon fibre into the mortar matrix gave rise to a pronounced surface response arc at intermediate frequencies 80 Hz–55 kHz between the electrode “spur” at lower frequencies 1–80 Hz and the bulk response arc at higher frequencies 55 kHz–10 MHz (Figure 1b‐1,b‐2). The bulk response of CEMe is a collective contribution from the ionic conduction through the connected pore network, the electronic conduction through the fibrous network, as well as the bulk polarization processes.^[^ ^72^ ^]^ The surface response arc corresponds to the strong surface conduction and double‐layer capacitance at the fibre/pore solution interface. The surface of the carbon fibre are reservoir to preserve water molecules thanks to the hydrophilic treatment of polycarboxylate superplasticizer, which stores cations and anions from pore solution, hence a pronounced surface impedance response.^[^ ^73^ ^]^ The presence of carbon fibres resulted in two tanδ peaks, one at 80 Hz with a value of 17 and another at 55 kHz with a value of 9, corresponding to the maximized dielectric relaxation processes (Figure 1b‐2). The 80 Hz peak reflects EP relaxation, contributing to surface conduction and aligned with *R*_s_; while the 55 kHz peak arises from surface EDLP relaxation, which transitioned into heat dissipation and contributed to the bulk ionic conduction process. With increasing frequency, the surface EDLP gradually diminished, giving way to bulk MWP dominance.^[^ ^74^ ^]^ This highlights that the cusp point between surface arc and bulk arc shared the same frequency with the high‐frequency tanδ peak, hence the *R_b_* should be the point to obtain the bulk conductivity σ of CEMe (Section S8.1, Supporting Information). As the value of loss tangent tanδ is positively proportional to the ability of dielectric polarization process to convert electrostatic energy into heat and conduction, the higher intensity (tanδ 17) low‐frequency peak compared to the lower intensity (tanδ 9) high‐frequency peak suggests stronger surface conduction (Figure 1b‐2). This is also justified by the smaller diameter of the surface arc than that of the bulk arc (Figure 1b‐1). Such phenomena were observed from CEMes with lower and intermediate fibre contents 0.1, 0.3, 0.5, and 0.8 vol% which were termed as S1, S3, S5, and S8, respectively (Figure S20a, Section S13.3, Supporting Information).

The Nyquist plot of CEMe with a high fibre content 1.5 vol% (termed as Figure 1c‐1; Figure S15, Supporting Information) shares the same pattern with CEMe of a low fibre content (sample Figure 1b‐1; Figure S1, Supporting Information), both consisting of an electrode “spur” at lower frequencies, a pronounced surface arc at intermediate frequencies, as well as a bulk arc at higher frequencies. As frequency increases, the EP relaxed and diminished at ≈130 Hz, followed by a pronounced surface EDLP which relaxed at ≈288 kHz. The bulk MWP operates at frequencies 288 kHz–10 MHz. In contrast to S1, the low‐frequency peak tanδ of S15 was detected to be 5, which was lower than its high‐frequency peak tanδ with a value of 7 (Figure 1c‐1). This suggests that the bulk conduction process of S15 is stronger than its surface conduction process. Likewise, this is also justified by the smaller diameter of the bulk arc than that of the surface arc (Figure 1c‐2). The same features were also observed from CEMes with a high fibre content, 1 and 1.2 vol%, which were termed as S10 and S12 respectively (Figure S20b, Section S13.3, Supporting Information). CEMes incorporating carbon fibres generally had high dielectric loss with the lowest bulk peak tanδ still being 3.8 for S8 at 5 °C (Figure S21a,b, Section S13.3, Supporting Information). This indicates that CEMe incorporating carbon fibre is lossy and conductive, which converts most of its electrical energy into heat and the conduction process, rather than being lossless for energy storage. S5 and S8 are relatively less lossy CEMes with lower bulk peak tanδ. The heating process resulted in higher tanδ than the cooling process, which means CEMes are more conductive under a warmer environment (i.e., receiving heat energy from the environment) than a cooler environment (i.e., dissipating heat energy to the environment).

The impedance responses of SPS at pristine condition, are also presented in Nyquist and Bode formats (Figure 1d‐1,d‐2) for identification of characteristic conduction parameters of a strong alkaline aqueous electrolyte solution with a pH of 13.58 and an IS (i.e., ionic strength) of 0.59 mol L^−1^ (Table S4, Section S2.2, Supporting Information), which is a continuum phase with single conducting mechanism, hence largely differentiating from CEMe with discontinuous and biphasic conducting mechanisms. The Nyquist plot (Figure 1d‐1) of SPS exhibits a typical strong aqueous conductor with minimized capacitive response, exhibiting in the form of a conductive tail in the absence of a capacitive loop throughout the entire frequency spectrum. In Figure 1d‐2, at frequencies above 56 kHz, where the relaxation of EP finishes, the capacitance of the liquid is too low to be detected by the impedance spectrometer, hence the negative values, whilst the conduction persists toward a plateau. Therefore, the *R_b_* of SPS is identified at 56 kHz where the Z″ reached zero (Section S8.1, Supporting Information). This rationale is suitable for all the measurements on conductive aqueous electrolytes using EIS (Section S12.2, Supporting Information).

### 2.2 Activation Energy Switching of CEMe

The temperature dependence of the bulk conductivity of CEMes is presented in Arrhenius plots (**Figure** **2**a–d), where the activation energy *E_a_* values for each sample are marked. Electrical impedance behaviours of CEMes with a standard dimension of 40 × 40 × 160 mm were evaluated under controlled thermal cycling conditions (Figure S2, Section S2.1, Figure S4, Section S3.1, Figure S7, Section S7, Supporting Information). The bulk conductivities of all samples were positively dependent on the temperature and had a hysteretic effect between heating and cooling stages. The *E_a_* of all samples had switching behaviour during thermal cycle, and the switching pattern exhibited strong dependency on the fibre content. For samples with no and lower fibre contents (PM, S1, and S3), the *E_a_* stayed unchanged between 20 and 60 °C and decreased at 70–90 °C during heating stage (Figure 2a‐1). Such switching behaviour was absent during the cooling stage (Figure 2a‐2). With intermediate fibre contents (S5 and S8), no switching behaviour was observed during the entire thermal cycle (Figure 2b‐1,b‐2). With higher fibre contents (S10 and S12), the *E_a_* had an upward switch during heating (Figure 2c‐1), whilst having a downward switch during cooling (Figure 2c‐2). However, for CEMe S15 which had the highest fibre content, non‐Arrhenius behaviour was present during heating where ln σ was non‐linearly correlated to $\frac{1}{T}$ (Figure 2d). Quantification of its *E_a_* was achieved via calculating the derivative of the exponential correlation between ln σ and $\frac{1}{T}$ (Section S9.2, Supporting Information). The *E_a_* of S15 increased consistently from 0.064 to 1.7 eV during heating from 20 to 90 °C (Figure 2d). During cooling, S15 exhibited almost the same behaviour as S12 (Figure 2c‐2). Complete development of impedance response with temperature is recorded in Section S13.1 (Supporting Information), showing continuous shrinkage of arc sizes during heating and extension of arc sizes during cooling for all samples.

The degree of *E_a_* switching (quantified by Δ*E_a_*, Figure 2e) and the temperature at which *E_a_* switching initiated (quantified by switching temperature *T*_s_, Figure 2f) were dependent on thermal stage and fibre content. At low fibre contents (0–0.3 vol%), Δ*E_a_* was negative and increased from −0.16 to −0.11 eV with the increasing fibre content during the heating stage, whilst being 0 eV during the cooling stage, suggesting a hysteresis behaviour where the degree of *E_a_* switching diminished as fibre content increased during heating but stayed constant during cooling. At higher fibre contents 1–1.5 vol%, Δ*E_a_* was positive and increased from 0.23 to 1.6 eV with the increasing fibre content during the heating stage, whilst being negative and decreased with the increasing fibre content during the cooling stage, suggesting also a hysteresis behaviour where the degree of *E_a_* switching enhanced as fibre content increased during both heating and cooling, but the degree of *E_a_* switching was more intensive during heating than that during cooling. The hysteresis behaviour of Δ*E_a_* was absent at intermediate fibre contents 0.5 and 0.8 vol%. During heating, *T*_s_ is equal to 60 °C at lower fibre contents 0–0.3 vol%, whilst decreasing from 50 to 20 °C at higher fibre contents 1–1.5 vol%. During cooling, higher *T*_s_ values were observed at higher fibre contents 1–1.5 vol%, suggesting also a hysteresis behaviour of *T*_s_.

The physical origin of the activation energy switching behaviours presented in Figure 2 is revealed in Sections 2.3–2.5 by a combination of experimentation and thermodynamic modelling.

### 2.3 Non‐Arrhenius Behaviour of Pore Solution

#### 2.3.1 Chemical Thermodynamic Modelling

**Figure 3:**
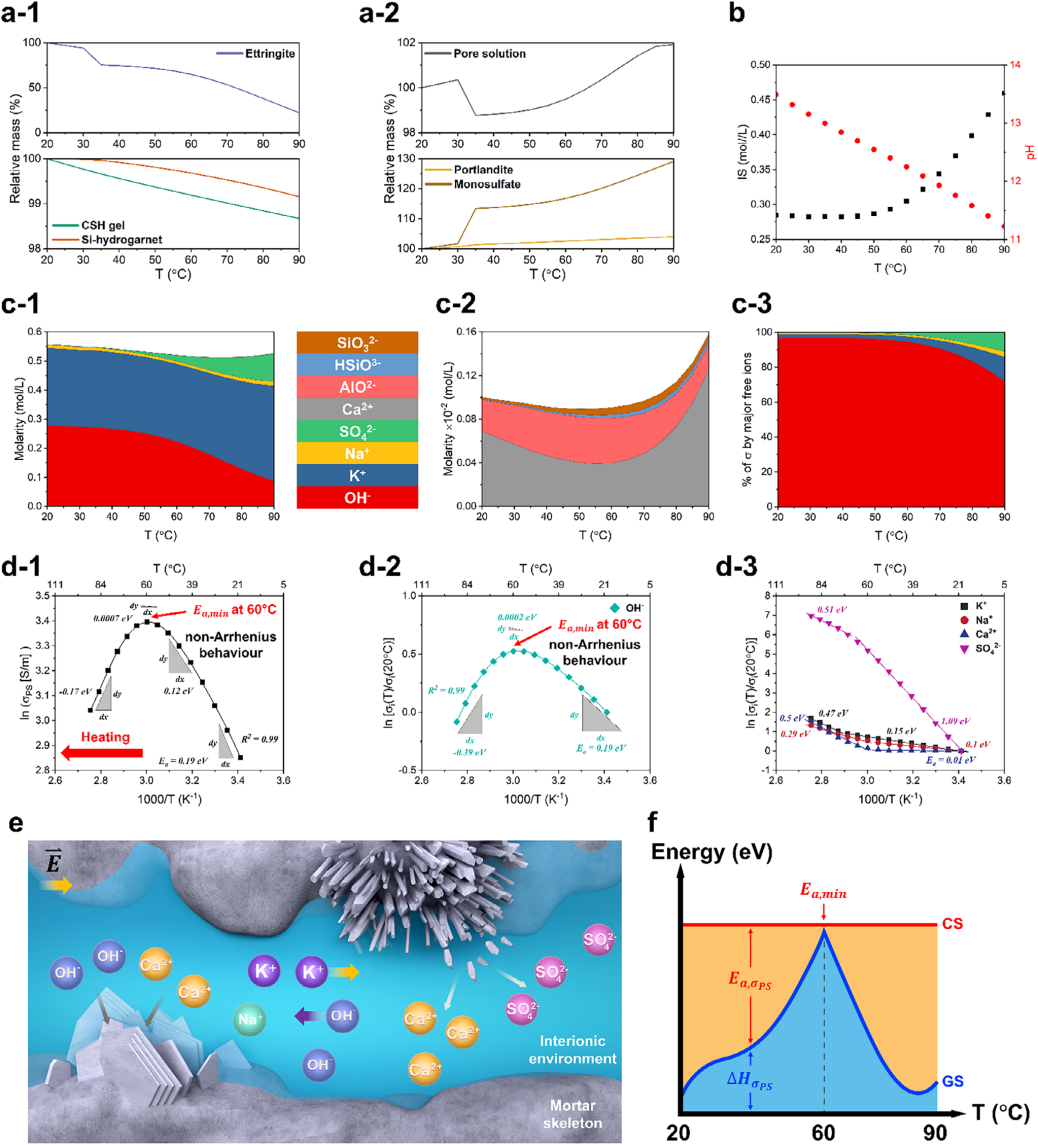
GEMs simulation of a hardened OPC system at temperature range between 20–90 °C for the thermally activated behaviour of pore solution and assemblages of solid phases. a‐1) Solid phases reduced in mass when the equilibrium temperature increased (C─S─H gel, ettringite, and Si‐hydrogarnet), relative mass percentage was calculated with respect to the pristine mass. a‐2) Solid phases increased in mass (portlandite and monosulfate) and total mass of pore solution which showed fluctuational behaviour. b) Development of ionic strength (IS) and pH of pore solution when the equilibrium temperature increased from 20 to 90 °C. c‐1) Major free ions in pore solution with molarity >5 × 10^−4^ mol L^−1^. c‐2) Ionic species with molarity <5 × 10^−4^ mol L^−1^. c‐3) percentage contribution of ionic species to the effective intrinsic electrical conductivity of pore solution (only the five major free ions K^+^, Na^+^, Ca^2+^, SO_4_^2−^, and OH^−^ were considered). d‐1) Non‐Arrhenius behaviour of intrinsic electrical conductivity of pore solution under confined conditions during heating from 20 to 90 °C. d‐2) Non‐Arrhenius behaviour of conduction by OH^−^ ion (conductivity is normalized with respect to the value at 20 °C. d‐3) Arrhenius behaviours of conduction by other major free ions). e) schematic illustration to present the precipitation of flake‐shaped portlandite (grey arrow) to reduce OH^−^ concentration and the decomposition of needle‐like ettringite (white arrow) to increase pore solution IS by increasing Ca^2+^ and SO_4_^2−^ concentration, the yellow arrow marks the direction of electric field and migration direction of positively charged ions, the purple arrow marks the migration direction of negatively charged ions, the grey solid boundary structure is mortar skeleton, the light blue background is water, and only the five major free ions are illustrated. f) schematic energy diagram to demonstrate the development of activation energy required for free ions to generate conduction during heating with a non‐Arrhenius behaviour caused by portlandite precipitation and ettringite decomposition: the blue curve GS refers to the ground state where the free ions were at equilibrium quantified by analogous enthalpy $\Delta H_{\sigma_{PS}}$ whose relationship with temperature was inversely proportional to the tangent of the polynomial fitting (i.e., $E_{a,\sigma_{PS}}/k_{B}$) in Equation S23 (Supporting Information) meaning lower $\Delta H_{\sigma_{PS}}$ requires higher $E_{a,\sigma_{PS}}$ to change from GS to CS; the red line CS refers to the conduction state where the free ions were having a long‐range transport in the direction of the electric field to generate electrical conduction.

In this section, the temperature dependence of the intrinsic conductivity of pore solution under confined conditions was realized based on chemical thermodynamic models (Section S7, Supporting Information). The condition confined here simulates the realistic behaviour of the OPC system (Section S1, Supporting Information) under heating from 20 to 90 °C, where the composition evolution of solid mineral phases and ionic species in pore solutions was produced under equilibrium (**Figure** **3**). The phase evolutions suggest that C─S─H gel, ettringite, and silicious hydrogarnet phases were destabilized during the heating process (Figure 3a‐1) while portlandite and monosulfate phases increased (Figure 3a‐2). The ettringite mass had the most remarkable decrease by 3.1 g accounting for 78% of its pristine mass, whereas the masses of portlandite and monosulfate had the most remarkable increase by 0.83 and 2.4 g, respectively, which accounts for 4% and 29% of their pristine masses. Temperature‐dependence of molarities of the five major free ionic species, K^+^, Na^+^, Ca^2+^, SO_4_^2−^, and OH^−^, are presented in Figure 3c‐1,c‐2, which dominate the intrinsic conductivity of pore solution.^[^ ^75^ ^]^ The reduction of OH^−^ molarity (Figure 3c‐1) was directly attributed by the increased precipitation of portlandite (for having a negative temperature dependence of solubility), which is the cause of the reducing pH from 13.5 to 11.2 (Figure 3b). The ettringite decomposes into monosulfate, which accompanies with the increase in monosulfate mass, as well as the increase in the molarities of Ca^2+^ (Figure 3c‐2) and SO_4_^2−^ (Figure 3c‐1) in pore solution. However, the development of Ca^2+^ molarity was fluctuational, which first decreased at 20–60 °C then increased at 60–90 °C (Figure 3c‐2). In conjunction with the continuously increasing portlandite mass and the decreasing OH^−^ molarity, as well as the absence of gypsum which was totally consumed to form ettringite (Section S7.3, Supporting Information), it is suggested that at 20–60 °C the portlandite precipitation prevailed the ettringite decomposition to decrease the overall Ca^2+^ molarity, whilst at 60–90 °C the ettringite decomposition intensified due to a lowered pH and higher temperature (pH = 12.2–11.2 at 60–90 °C, Figure 3b), which prevailed the portlandite precipitation to increase the overall Ca^2+^ molarity.^[^ ^76^, ^77^ ^]^ The degree of increase in SO_4_^2−^ molarity was the greatest (i.e., 40 000% of its pristine molarity) amongst all free ions, which led to large increases in the molarities of strong bases K^+^ (i.e., 23%) and Na^+^ (i.e., 30%) for charge neutralization (Figure 3c‐1). The silicious hydrogarnet is metastable at room temperature.^[^ ^78^ ^]^ It decomposes as soon as the heating is initiated, which corresponds to the changes in the concentrations of AlO_2_^−^, Ca^2+^, SiO_3_^2−^, and HSiO_3_^−^, but all are too low to be considered influential (Figure 3c‐2). The nanocrystal structure of C─S─H is stable under 105 °C hence it is only losing a slight amount of interlayer water for only 0.6 g (i.e., 1.3% of pristine mass). Molarity changes in other large ions (considered minimal) and neutral ion clusters (excluded from the conduction process) through heating are all recorded in Section S7 (Supporting Information). The ionic strength (IS) remained relatively stable between 20 to 50 °C whilst increased continuously and sharply from 0.3 to 0.47 mol L^−1^ from 50 to 90 °C (Figure 3b), indicating a continuously intensifying interionic environment beyond 50 °C with strong ion–ion electrostatic force to drag the free ions from migrating.^[^ ^79^ ^]^ It is suggested that the increase of both the IS and pore solution mass (Figure 3a‐2) at higher temperatures is due to the increase in SO_4_^2−^, K^+^, and Na^+^ molarities as a result of large ettringite decomposition. The ettringite, impregnated with thermodynamically developing pore solution, if skipping metastable phase state, is expressed by the following reaction to decompose:

(2) $$\begin{array}{ccc} & & 3CaO\cdot Al_{2}O_{3}\cdot 3CaSO_{4}\cdot 32H_{2}O\\ & & \;\to 3CaO\cdot Al_{2}O_{3}\cdot CaSO_{4}\cdot 12H_{2}O+2Ca^{2+}\\ & & \;+\;2S{O_{4}}^{2-}+20H_{2}O \end{array}$$

which intensified under decreasing pH and increasing temperature. The 1.7% reduction in pore solution mass at 20–35 °C is possibly a result of moisture loss (Section S5, Supporting Information).

The intrinsic electrical conductivity of pore solution calculated through the modified Nernst–Einstein relation using temperature‐dependent data of the five major free ions via thermodynamic models (Section S8.2, Supporting Information), is shown in Figure 3d‐1, showing that the intrinsic conductivity of pore solution exhibited non‐Arrhenius behaviour. During heating from 20–60 °C, *E_a_* of pore solution showed positive values indicating a positive temperature dependence of conductivity, which decreased from 0.19 eV at 20 °C to a minimum of 0.0007 eV at 60 °C (i.e., the *E*_*a*,*min*_ value should be infinitely close to zero if the model was infinitely accurate, Section S9.2, Supporting Information). During heating from 60–90 °C, the *E_a_* showed negative values indicating a negative temperature dependence of conductivity, whose absolute value increased until reaching a maximum of 0.17 eV at 90 °C. This means when heating from 20–90 °C the interionic environment in pore solution first became increasingly easier for free ions to migrate reaching an optimum at 60 °C, then became increasingly resistive. To gain a deeper insight, *E_a_* of conduction process of individual ions were obtained. It is obvious that OH^−^ conduction also exhibited non‐Arrhenius behaviour being consistent with that of pore solution, whose optimal interionic environment was also located at 60 °C with a *E*_*a*,*min*_ of 0.0002 eV (Figure 3d‐2). In contrast, other major free ions, K^+^, Na^+^, Ca^2+^, and SO_4_^2−^ exhibited Arrhenius behaviour and positive temperature dependence throughout the heating process, whose *E_a_* switched at *T*_s_ = 70, 55, 55, and 65 °C, respectively (Figure 3a‐3). The *E_a_* of K^+^, Na^+^, and Ca^2+^ had an upward switch indicating that high temperature had detrimental effect on their migration, whilst the *E_a_* of SO_4_^2−^ had a downward switch indicating that high temperature facilitated its migration, yet with a high *E_a_* of 0.51 eV. Based on the modified Nernst–Einstein relation, the percentage contribution to intrinsic conductivity of pore solution by OH^−^ decreased from 96 to 73% through heating maintaining at a dominant level, despite the large increase in the percentage contribution of SO_4_^2−^, K^+^, and Na^+^ (Figure 3c‐3) where Ca^2+^ was even visually unnoticeable. The OH^−^ conduction dominates the effective behaviour of pore solution owing to three reasons: 1) the concentration dominance, 2) the highest mobility (or diffusivity) of OH^−^ amongst the major free ions (Table S4, Section S2.2, Supporting Information), 3) the unique Grotthuss hopping mechanism of OH^−^ in addition to its migration to form “dual conduction” mechanism. Therefore, referring back to the consistency of non‐Arrhenius behaviour between OH^−^ conduction and pore solution, it is suggested that the state of OH^−^ conduction in reaction to the change of interionic environment is the dominant reason to control the effective behaviour of pore solution. This, in turn, corresponds to the continuous ettringite decomposition (to increase IS) and portlandite precipitation (to reduce OH^−^ molarity) through heating (Figure 3e,f). An overly crowded interionic environment to hinder OH^−^ migration, as well as a reducing OH^−^ molarity, are the main reasons for a negatively temperature‐dependent intrinsic conductivity of pore solution (with an increasing |*E_a_* |) at 60–90 °C. Furthermore, it is suggested that the *E*_*a*,*min*_ at *T*_s_ = 60 °C (Figure 3f) is a collective work of precipitation/dissolution, chemical equilibria, and ion migration kinetics, which are all thermodynamically dependent and can change if modifying the mix design.

It should be noted that the thermodynamic modelling employed in this study describes the equilibrium state of the chemical system, rather than the transient kinetic processes that may occur during temperature variation. However, given that the experimental measurements were conducted after sufficient stabilization to ensure steady‐state conditions, such equilibrium modelling is well‐suited for elucidating the governing relationships between temperature, phase assemblage, and pore solution composition relevant to the observed conduction behaviour in this study.

#### 2.3.2 Reproduction of Hysteresis

**Figure 4:**
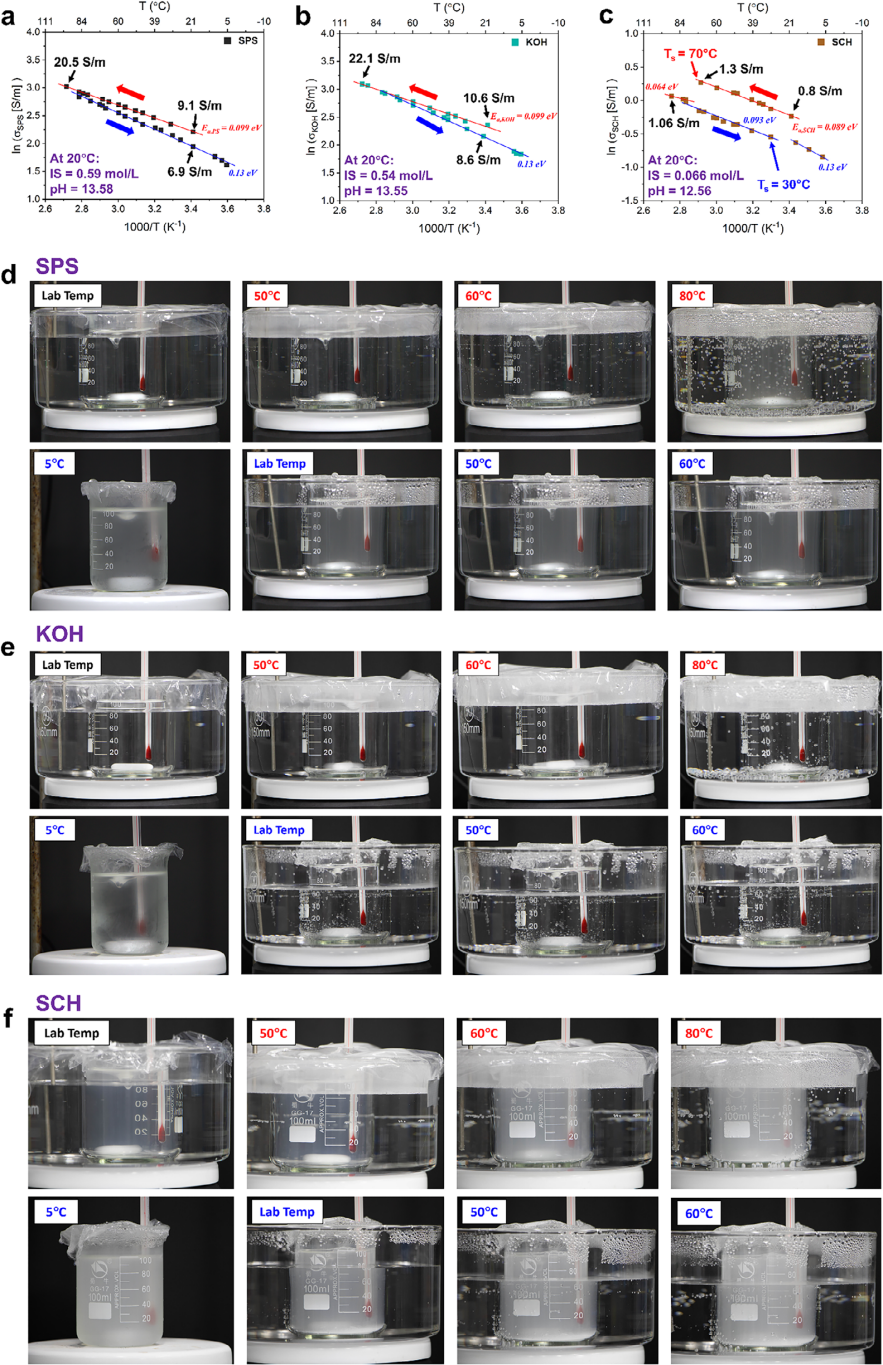
Arrhenius behaviours of bulk conductivity of simulated pore solution (abbrev. SPS, IS = 0.59 mol L^−1^, pH 13.58, at 20 °C), potassium hydroxide solution (abbrev. KOH, IS = 0.54 mol L^−1^, pH 13.55, at 20 °C), and saturated calcium hydroxide solution (abbrev. SCH, IS = 0.066 mol L^−1^, pH 12.56, at 20 °C) at free form condition under thermal cycle to reproduce the hysteresis of conductivity and activation energy between heating and cooling. a) SPS. b) KOH. c) SCH. d,e) real‐time photograph of the nucleation and re‐dissolution process of solutes in SPS, KOH, and SCH during thermal cycle.

The same thermal cycle was employed on as‐prepared SPS to reproduce the hysteresis of intrinsic conductivity and activation energy switching behaviour of pore solution between heating and cooling (**Figure** **4**a). To understand the influence of Ca^2+^ concentration on the hysteresis behaviour which is associated with the thermodynamic solubility behaviour of portlandite, the as‐prepared KOH and SCH solutions were also evaluated and compared (Figure 4b,c, Tables S4,S5, Section S2.2, Supporting Information). The precipitation/re‐dissolution process of portlandite was photographed at characteristic temperatures as soon as showing visually noticeable increase/decrease in precipitated products (Figure 4d–f). Molarities of the five free ions in SPS (Table S4, Section S2.2, Supporting Information) are all higher than that in the pore solution by GEMs simulation (Figure 3c‐1). This is because the ion speciation phenomenon in highly concentrated solution was considered in thermodynamic modelling (Section S7.2, Supporting Information), whilst being ruled out in NIST and Moragues et al.’s methods which overestimated the molarities of the five free ions. Nevertheless, combining NIST and Moragues et al.’s methods prioritize the state of saturation (or super‐saturation) of Ca(OH)_2_ amongst the solutes (Section S2.2, Supporting Information), which is a realistic situation of pore solution in the OPC system and is beneficial for visualizing precipitation/re‐dissolution of portlandite experimentally. The Ca^2+^ molarity was the lowest in KOH (0 mol L^−1^), followed by 0.012 mol L^−1^ in SPS and then the highest of 0.022 mol L^−1^ in SCH. For SCH, it exhibited pronounced *E_a_* switching behaviour with three switching stages: during heating a downward switch at *T*_s_ = 70 °C from 0.089 to 0.064 eV, during cooling at 90 °C an upward switch of *E_a_* from 0.064 to 0.093 eV, and finally during cooling at 30 °C an upward switch from 0.093 to 0.13 eV. Referring to Figure 4f, the appearance of SCH carried a heavy degree of cloudiness. Significant precipitation appeared at 60 °C and intensified at 80 °C during heating, which stayed consistent until the cooling end. It is expected that the precipitation consisted of portlandite and calcite (i.e., inevitable carbonation). The decrease in *E_a_* during heating was due to a less crowded ionic environment for free Ca^2+^ and OH^−^ ions to migrate which was contributed by the nucleation of portlandite.^[^ ^80^, ^81^ ^]^ The increase in *E_a_* during cooling was due to the re‐dissolution of portlandite and reduced thermal energy but only minimally because it is more difficult for larger crystalline particles to dissolve.^[^ ^82^ ^]^ Unlike the pore solution predicted from thermodynamic models, SPS shows no *E_a_* switching behaviour (Figure 4a) during thermal cycling, attributable to its free‐form condition unaffected by thermally induced solid‐phase decomposition. However, visually noticeable cloudiness still took place at 80 °C during heating (Figure 4d), but being significantly lighter than that of SCH (Figure 4f). Therefore, lower Ca^2+^ concentration, as in SPS, contributes to less significant precipitation and re‐dissolution process of portlandite, hence a none (or undiscernible) *E_a_* switching behaviour. Referring back to Figure 3c‐2, the pore solution in the thermodynamic model showed that the Ca^2+^ concentration stayed below 0.0016 mol L^−1^, which was much lower than the SPS and SCH solutions in the experiment. Therefore, it is safe to propose that during cooling under realistic confined OPC conditions, the *E_a_* switching behaviour of the intrinsic conductivity of pore solution, was non‐existent. The IS was not the dominant factor to control the *E_a_* switching behaviour of pore solution, at least below 0.59 mol L^−1^ in this study. This is justified by the non‐existence of *E_a_* switching behaviour of KOH solution throughout the thermal cycle (Figure 4b), although having higher IS of 0.54 mol L^−1^ than SCH (0.066 mol L^−1^, Figure 4c), but no precipitation of solutes to affect the interionic environment. It should be noted that the hysteresis behaviour existed between heating and cooling for all solutions, where the *E_a_* during heating was lower than that during cooling and the conductivity during heating was higher than that during heating. This is because heating simulates a positive energy transfer (i.e., energy receiving process of the sample from the environment) where the inherent ion mobility increases, the viscosity of water decreases, and the charge carriers have an easier desolvation process, which is all vice versa during cooling (i.e., simulates a negative energy transfer).^[^ ^83^, ^84^ ^]^ This can also be justified by KOH solution without precipitation throughout the thermal cycle (Figure 4b,e), but still with a hysteresis behaviour. Complete development of the impedance response of solutions with temperature is recorded in Section S13.2, Supporting Information.

### 2.4 Thermally Induced Alteration of Pore Network

Liquid‐filled pore network provides a physical pathway for ion migration. Since the conduction of PM is totally ionic, the physical hindrance/facilitation of the pore network over ion migration, can be described by the formation factor, *F*_*ion*, *PM*_, which is the ratio of the intrinsic conductivity of the pore solution σ_*PS*_ over its bulk conductivity σ_*PM*_:

(3) $$F_{ion,PM}=\frac{\sigma_{PS}}{\sigma_{PM}}$$

Physics‐based function of formation factor for ionic conduction, *F_ion_*, should be constructed by connected porosity ϕ, pore tortuosity τ, and pore fractal dimension *D_s_*,^[^ ^85^, ^86^, ^87^ ^]^ which are the geometric characteristics of the pore network to affect ion migration:

(4) $$F_{ion}=f\left(\phi ,\tau ,D_{s}\right)$$

The connected porosity ϕ defines the flux of ions to migrate in the cement matrix. The pore tortuosity τ (inverse of connectivity) weighs the effective conduction pathways as ions are blocked as soon as encountering solid pore walls and prefer to migrate through pores with less curvature (the “least impedance principle”). The pore fractal dimension *D_s_* measures the roughness (or morphological complexity) of pore walls which is proportional to the chances for ions to meet solid barriers^[^ ^87^ ^]^ (Sections S11.2.3,S11.2.4, Supporting Information).

**Figure 5:**
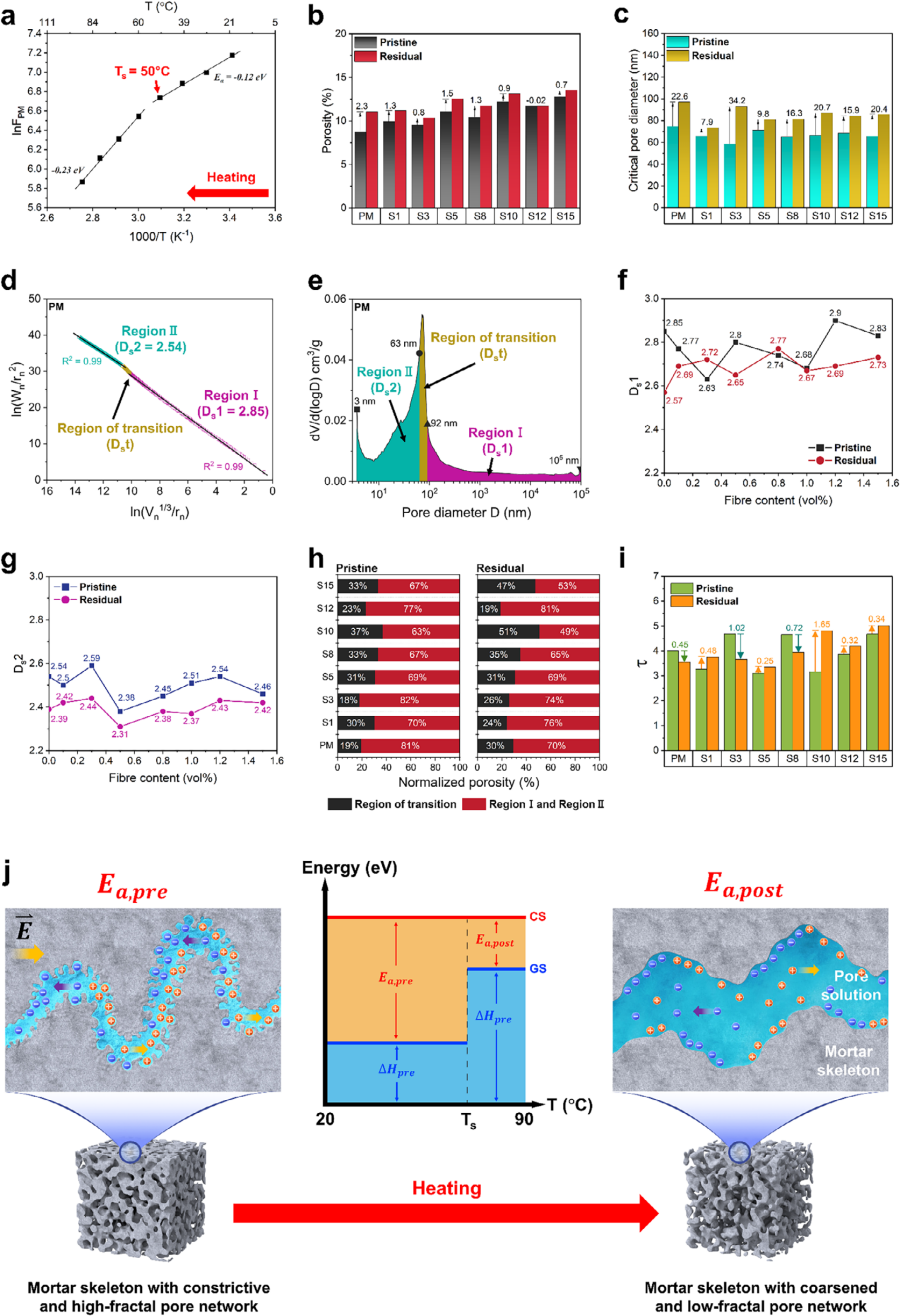
Thermally induced alteration in pore network described by formation factor and determined by mercury intrusion porosimetry (MIP). a) Arrhenius behaviour of *F*_*ion*,*PM*_ during heating with an upward *E_a_* switching at *T*_s_ = 50 °C. b) porosity at pristine (without any thermal treatment at laboratory temperature) and residual (after completion of thermal cycle at laboratory temperature) conditions measured by MIP. c) critical pore diameter at pristine and residual conditions measured by MIP. d) determination of pore fractal dimension at characteristic fractal regions (PM at pristine condition as an example). e) projection of multi‐fractal regions onto differential intrusion plot for the calculation of porosity percentage of each fractal region (area under curve): regionII for smaller pores with a fractal dimension of D_s_2, regionI for larger pores with a fractal dimension of D_s_1, and region of transition for intermediate‐sized pores with a fractal dimension of D_s_t. f) D_s_1 at pristine and residual conditions. g) D_s_2 at pristine and residual conditions. h) porosity percentage of the fractal regions at pristine and residual conditions. i) pore tortuosity at pristine and residual conditions. j) schematic illustration of the opening effect of the pore network in PM during heating to physically facilitate ion migration, hence the reduced effective *E_a_* of conduction: the pore network transitioned from constrictive and high‐fractal into coarsened and low‐fractal through the heating process. The free ions were easily blocked at solid pore walls in a constrictive and high‐fractal pore network (positive ions are accumulated on the right side and negative ions are accumulated on the left side under a right‐directed electric field). Such a mechanism can also generate strong Maxwell–Wagner interfacial polarization. When the temperature rises, the free ions were less likely to be blocked at solid pore walls in a coarsened and low‐fractal pore network being easier to migrate hence reduced effective *E_a_* for conduction. In the middle energy diagram, the blue line GS refers to the ground state where the free ions were at an equilibrium state quantified by analogous enthalpy Δ*H* (Δ*H_pre_*  <  Δ*H_post_*), lower Δ*H* requires higher *E_a_* to change from GS to CS, the red line CS refers to the conduction state where the free ions were generating electrical conduction, hence *E*_*a*,*pre*_  >  *E*_*a*,*post*_. Only the free ions in the bulk pore solution for conduction were illustrated, and electrical double layers were omitted. The yellow arrow marks the direction of the electric field and migrating direction of positively charged ions, the purple arrow marks the migrating direction of negatively charged ions, the grey solid structure collectively describes the mortar skeleton, the light blue background impregnated in the mortar skeleton is bulk H_2_O.

Combining the experimental results in Figure 2 and the thermodynamic modelling results in Figure 3, thermally induced alteration of the pore network of PM was quantified using Equation (3), as presented in Arrhenius form in **Figure** **5**a. As the temperature increased from 20 to 90 °C, *F*_*ion*,*PM*_ decreased continuously. This suggests that the pore network became increasingly less resistive for ion migration as the temperature increased. Negative value of *E_a_* value means negative temperature dependence of *F*_*ion*,*PM*_. The above behaviours were consistent for all samples (Section S14.1, Supporting Information). Therefore, |*E_a_* | calculated in Figure 5a should be the lowest energy required for the architecture of pore network to change. The |*E_a_* | was 0.12 eV switching upward to 0.23 eV at *T*_s_ = 50 °C, which indicates that although facilitating ion migration with increasing temperature, the pore structure architecture changed less intensively between 50 and 90 °C than that between 20  and 50 °C.

Figure 5b,c showed an increased total porosity and coarsened pore size distribution after thermal cycle. In detail, connected nanoscale pores feature diameters <200 nm predominated the coarsening process (Section S11.2.5, Supporting Information). Using PM as an example, the multi‐fractal characteristics of the samples are categorized into three regions with each having a consistent value of *D*_s_ (Figure 5d). The differential MIP intrusion plot (Figure 5e) provides a better visualization of the size dependency of the pore fractality. The lower and upper pore size boundaries of each fractal region were marked, and the region of transition (63–92 nm) corresponds to capillary pores. The area under the curve was used to determine the percentage of each fractal region with respect to the total ϕ. Both *D*_s_1 and *D*_s_2 decreased after thermal cycle, except for the *D*_s_1 of S3 and S8, which was possibly influenced by the selection of fragments for MIP test (Figure 5f,g). The collective porosity of regionI and II comprised over two‐thirds of the total porosity throughout the thermal cycle, except for S10 and S15 at the residual state which was still ≈50% of the total porosity (Figure 5h). Therefore, the reduction in *D*_s_1 and *D*_s_2 dominated the overall multifractal behaviour of the sample volumetric‐wise, suggesting that the surface of pore walls had been smoothened after the thermal cycle. Upon completion of the thermal cycle, the changes in pore tortuosity varied under different percolation regimes: it decreased at lower fibre contents for PM and S3, whilst increased at intermediate and higher fibre contents except for S8. The increase in fibre content can contribute to the reduction of C─S─H gel in the bulk cement matrix due to more C─S─H gels are formed on the hydrophilic fibre surface.^[^ ^72^ ^]^ Therefore, it is proposed that the decreased pore tortuosity at lower fibre contents corresponds to a direct coarsening process of nanopores, whilst the increased pore tortuosity at intermediate and higher fibre contents corresponds to a disordered connectivity rearrangement of unstable hydration products (e.g., portlandite, ettringite,) and unreacted cement due to the lack of thermally stable C─S─H gel in the bulk cement matrix. Critically, pore tortuosity was hence an inconsistent index for representing the opening process of the pore network during thermal cycle owning to inconsistent behaviour through the percolation regime.

It is suggested that the facilitation of the pore network on ion migration, where *F_ion_* increased with increasing temperature, was through an opening effect: the connected porosity increased, the pore size distribution coarsened, and the pore fractal dimension decreased (Figure 5j). Therefore, for PM whose conduction was totally ionic, the opening effect of the pore network was the predominant reason for controlling the Arrhenius behaviour of bulk conductivity with a downward switch of bulk *E_a_* (Figure 2a), rather than the non‐Arrhenius behaviour of the pore solution. In this study, the moisture state of the samples can be defined as “near water saturated”. Through mass recording, the moisture loss was intensive during heating whilst being marginal during cooling (Section S5, Supporting Information). Therefore, the underlying mechanism for such an opening effect can be ascribed to the microcracking during heating due to a mismatch of the coefficient of thermal expansion (CTE) between solid and liquid components, where a positive pore pressure was induced upon gas production (Table S3, Section S1, Figure S10c‐1, Section S7.3, Supporting Information). More importantly, decomposition of ettringite and dehydration of C─S─H gel can create additional gaps and voids both in matrix and fiber–matrix interface, leading to opened pore network (as depicted in Figure 3a‐1 and the BSE images in Figure S11, Section S11.1, Supporting Information). Such an opening process was constantly operative with the increasing temperature until a discernible *E_a_* switching at *T*_s_ (Figures 5j,2a). During cooling, the water molecule carried less kinetic energy, hence a reduced positive pore pressure comparing to heating. Liquid and solid components can have reversed thermal strain to cause more microcracking.^[^ ^88^ ^]^ Also, it is possible for metaettringite to recover back to ettringite. However, the above processes were only partially reversible being less intensive than that during heating because cooling is a process of energy dissipation rather than receiving. Therefore, pore network opening during cooling contributed minimally to the overall *E_a_* switching effect.

### 2.5 Thermally Induced Alteration of Fibrous Network

At temperatures 5–90 °C, the intrinsic conductivity of carbon fibre has a positive temperature dependence with a constant *E_a_* of 0.0027 eV throughout the thermal cycle. Also, its CTE maintained at −0.7 × 10^−6^ °C^−1^, indicating a thermo‐mechanical behaviour of shrinking during heating and expanding during cooling. Such extent is two orders of magnitude lower than that of hardened cement, being the most thermo‐mechanically stable component in CEMe (Table S3, Figure S1, Section S1, Supporting Information). In analogous to the pore network, the architecture of the fibrous network can be quantified through formation factor, which is a function of the fibre content ϕ_*f*_, number of contacting points *N_c_*, and number of tunnelling gaps *N_t_*:

(5) $$F_{e}=\frac{\sigma_{CF}}{\sigma_{e}}=f\left(\phi_{f},N_{c},N_{t}\right)$$

where *F_e_* is the formation factor of the electronic conduction pathway (i.e., fibrous network), σ_*CF*_ the intrinsic conductivity of carbon fibre (in S m^−1^), and σ_*e*_ the effective electronic conductivity through the fibrous network (in S m^−1^).

**Figure 6:**
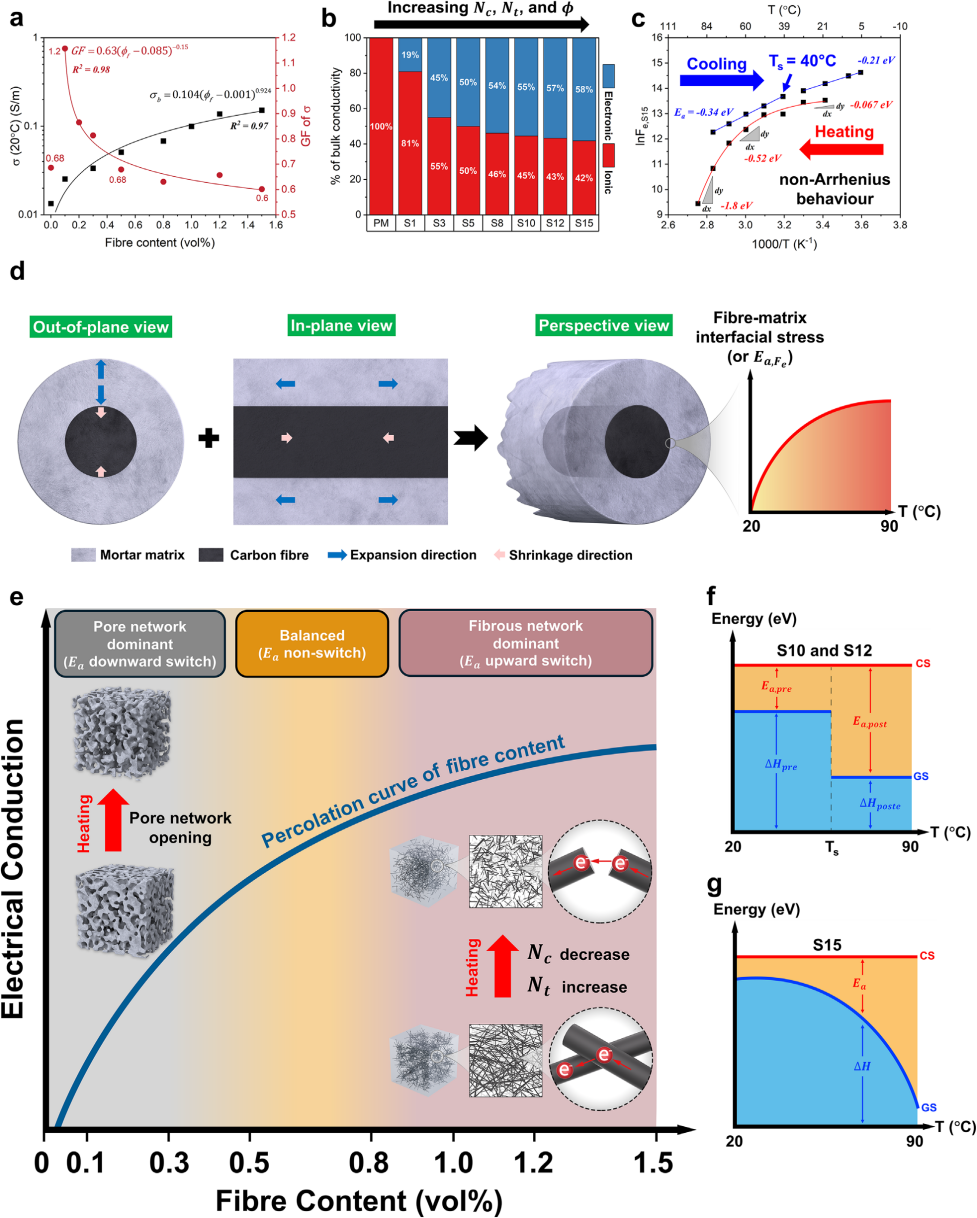
Thermally induced alteration in the fibrous network described by the formation factor (i.e., S15 as an example) and percolation‐governed competition between pore and fibrous network. a) Percolation of carbon fibre in cement matrix with a percolation threshold of 0.001 vol% and a percolation exponent of 0.924, as well as the gauge factor (GF) of bulk conductivity with respect to moisture content for the quantification of the percentage of ionic conduction with respect to effective bulk conductivity through fibre percolation. b) Percentage of ionic and electronic conduction with respect to effective bulk conductivity through fibre percolation at the samples’ “near water saturated” condition. c) Non‐Arrhenius behaviour of formation factor *F*_*e*,*S*15_ of the fibrous network in CEMe during heating and its Arrhenius behaviour with *E_a_* switching during cooling using S15 as an example (i.e., “e” denotes the electronic conduction pathway). d) schematic illustration for the development of fibre–matrix interfacial stress during heating which is positively correlated to $E_{a,F_{e}}$, the mortar matrix expands with positive CTE of 15 × 10^−6^ °C^−1^ which was two orders of magnitude higher than the carbon fibre shrinkage with negative CTE of −0.7 × 10^−6^ °C^−1^. e) schematic illustration of percolation‐controlled activation energy switching behaviour during heating (the percolation curve was obtained from Figure 6a and only the fibre content used in this study were marked on horizontal axis): PM, S1, and S3 are controlled by pore network architecture with *E_a_* downward switching Arrhenius behaviour; S5 and S8 had balanced competition between pore and fibrous network with *E_a_* non‐switching Arrhenius behaviour; S10, S12, and S15 are controlled by fibrous network with *E_a_* upward switching where S10 and S12 had Arrhenius activation energy switching behaviour and S15 had non‐Arrhenius behaviour. f) activation energy switching diagram for S10 and S12 during heating: the blue line GS refers to the ground state where the electrons/holes were at equilibrium state quantified by analogous enthalpy Δ*H* (Δ*H_pre_*  >  Δ*H_post_*), lower Δ*H* requires higher *E_a_* to change from GS to CS, the red line CS refers to the conduction state where the electrons/holes were generating electrical conduction with *E*_*a*,*pre*_  <  *E*_*a*,*post*_. g) non‐Arrhenius activation energy behaviour for S15 during heating: analogous enthalpy Δ*H* continuously decreased indicating a continuously increasing high activation energy pathway *N_t_*, hence the continuously increasing effective *E_a_* with a *T*_s_ as early as 20 °C.

The increase of fibre content in the cement matrix can induce a percolating process where *N_c_*, *N_t_*, and the porosity ϕ all increase (Figures 5b,**6**b), hence the increase in bulk conductivity both ionically and electronically. Such a percolating process is well‐approximated by the percolation theory which was validated with an exponent of 0.924 and a percolation threshold of 0.001 vol% (Figure 6a). The contribution of ionic conduction to the overall bulk conductivity have been determined by analyzing the gauge factor (GF) of bulk conductivity (Figure 6a), which was obtained through a long‐term drying experiment (Section S15, Supporting Information). The percentage of ionic conduction dropped from 100 to 42% and the percentage of electronic conduction increased from 0 to 58% as the fibre content increased from 0 to 1.5 vol% (Figure 6b). For the “near water saturated” and well fibre‐percolated CEMe S15, the ionic conduction still took up 42% of its bulk conductivity, suggesting the importance of both ionic and electronic conductions in CEMe.

In Figure 6c, the development of the formation factor for the fibrous network *F_e_* under thermal cycle was plotted in Arrhenius form using the same method as described in Section 2.4 and Equation (5). CEMe S15 is presented as an example. The σ_*e*_ was obtained under the assumption that the electronic conduction was fixed at 58% throughout the entire thermal cycle since the moisture loss was minimal and the fibre content was constant. The *F*_*e*,*S*15_ had a negative temperature dependence with negative *E_a_* values throughout the entire thermal cycle, which exhibited non‐Arrhenius behaviour during heating and Arrhenius activation energy switching behaviour during cooling. This was consistent with its behaviour of bulk conductivity (Figure 2c‐2,d). The reducing *F*_*e*,*S*15_ through heating suggests a facilitation effect of fibrous network for electrons/holes transport. However, the continuously increasing absolute activation energy value of *F*_*e*,*S*15_ from 0.067 to 1.8 eV (denoted as $|E_{a,F_{e}}|$) suggests that the fibrous network became increasingly more difficult to be altered through heating. It is proposed that this corresponds to the increasing fibre–matrix bonding stress with the increase in fibre–matrix interfacial thermal strain due to fibre–matrix CTE mismatching (Figure 6d). The increasing *F*_*e*,*S*15_ through cooling suggests a hinderance effect of fibrous network for electrons/holes transport. The $|E_{a,F_{e}}|$ had a downward switch from 0.34 to 0.21 eV at *T*_s_ = 40 °C, which means the alteration of fibrous network at 30–5 °C was easier than that at 80–40 °C during cooling. Likewise, this corresponds to the decreasing fibre–matrix bonding stress from the reversed fibre–matrix straining during cooling. The hysteresis, where *F*_*e*,*S*15_ was lower during heating than cooling, was indicative of a more disconnected fibrous network during heating than that during cooling. This can be ascribed to two reasons: 1) hysteresis of thermo‐mechanical straining of fibre and cement matrix between heating and cooling, 2) debris from the cement matrix microcracking along with precipitation and decomposition products to disconnect fibrous network (as shown in BSE imaging Figure S11 (Supporting Information) where the presence of portlandite and ettringite is universal around carbon fibre), a process that was more intensive during heating than cooling. Such hysteresis of *F_e_* was consistent amongst all CEMes with negative *E_a_* values during thermal cycle (Section S14.2, Supporting Information).

The carbon fibres are present in a rather restricted environment, that is, the hardened cement matrix. The CTE of “near water saturated” cement matrix is around two orders of magnitude higher than that of carbon fibre, which is, in particular, positive (Figure 6d, Table S3, Section S1, Supporting Information). That means the fibres were collectively displaced away from each other along with the surrounding cement matrix, driven by interfacial stress at the fibre–matrix boundary during heating. Therefore, it is unlikely that *N_c_* increases during heating. Instead, a decrease in *N_c_* is expected to be accompanied by an increase in *N_t_*, where contact conduction diminishes and tunnelling transmission becomes more prominent. In turn, the facilitation effect of the fibrous network for electronic conduction, which is reflected by a decrease in *F_e_*, can suggest the enhancement of tunnelling transmission. The tunnelling effect can be enhanced by enhancing voltage excitation and heat energy transfer flux, and by reducing the tunnelling gap, as well as by any means to enhance electrons/holes kinetic energy.^[^ ^89^, ^90^, ^91^ ^]^ Hence during cooling, where CEMe was dissipating heat energy toward the environment, the tunnelling effect was less pronounced than that during heating where CEMe was receiving heat energy from the environment.

### 2.6 Percolation‐Governed Conduction Network Competition

The non‐Arrhenius behaviour of the pore solution (Figure 3d‐1) and the reversible Arrhenius behaviour of the carbon fibre (Figure S1, Section S1, Supporting Information) were both inconsistent with the observed effective behaviours of CEMes across the entire percolation regime including Arrhenius activation energy switching, Arrhenius non‐switching, and the non‐Arrhenius behaviour (as presented in Figure 2). Therefore, the effective behaviour of CEMe is unlikely to be governed by the thermally activated intrinsic conductivity of the pore solution and the carbon fibre. Instead, it is primarily governed by the competing interactions between the architecture of the pore network and the fibrous network. For the fibrous network, a standard value for the activation energy of contact conduction (i.e., denoted as $E_{a,N_{c}}$) is determined to be 0.064 eV (as in Figure 2d) because S15 was universally percolated by carbon fibres (Figure 6a). Considering the voltage applied in this study (i.e., RMS = 707.107 mV), the activation energy for the tunnelling effect is 0.36 eV (i.e., denoted as $E_{a,N_{t}}$).^[^ ^92^ ^]^ For the pore network, 0.24 and 0.084 eV can be taken as standard values for activation energy of ionic conduction through pore network of a standard mortar (i.e., denoted as *E*_*a*,*ionic*_ as in Equation (1)) at 20–60 °C and at 60–90 °C during heating, respectively. Based on the “least impedance” principle,^[^ ^93^ ^]^ the fibre–fibre tunnelling gap is the conduction pathway with the highest activation energy, making it the most difficult for electrons/holes to transmit, followed by the pore network, and finally the fibre–fibre direct contact points with the lowest activation energy hence being the easiest conduction path, that:

(6) $$E_{a,N_{t}}\;>\;E_{a,ionic}\;>\;E_{a,N_{c}}$$

Therefore, upon heating and according to the physical concept defined in Equation (1), the competitive interaction between the pore network and the fibrous network governing the alteration in activation energy switching direction can be interpreted as follows: the pore network opens to facilitate ion transport (i.e., increase in low activation energy channel), meanwhile the fibrous network tends to be disconnected, leading to an increase in *N_t_* (i.e., increase in high activation energy channel). Through the entire percolation regime, the pore network prevailed over the fibrous network in such competition at lower fibre contents, whereas the fibrous network became dominant at higher fibre contents (Figure 6e). The downward *E_a_* switching of S1 and S3 (Figure 2a‐1) indicates that the increase in low *E_a_* path through pore network opening (Figure 5j) had prevailed over the increase in high *E_a_* path through increase in *N_t_* of fibrous network (Figure 6e). As the percolation of fibre content proceeds where both *N_c_* and *N_t_* were increased (Figure 6a,b), the non‐existence of *E_a_* switching for S5 and S8 through heating (Figure 2b‐1), was attributed to a dynamic, collectively counterbalanced equilibrium amongst the remaining *N_c_* of fibrous network, the increasing *N_t_* of fibrous network, and the opening of pore network. Ultimately, the upward *E_a_* switching of S10 and S12 (Figure 2c‐1), as well as the non‐Arrhenius behaviour of S15 (Figure 2d), can be attributed to the increase in *N_t_* of fibre network, which had prevailed the opening of pore network and the remaining *N_c_* of fibrous network. In addition, the decrease in *T*_s_ as the fibre content increased from 1 to 1.5 vol% (as shown in Figure 2f) supports the percolation‐governed activation energy sequence at higher fibre contents. Since S10, S12, and S15 were all electronically dominant CEMes and the fibre shrinkage could initiate immediately as soon as being heated. S15 exhibited the highest *N_c_* and *N_t_* (Figure 6b) therefore its *E_a_* switched at a *T*_s_ as early as 20 °C, accompanied by a non‐Arrhenius behaviour where the disconnecting process of fibrous network showed the greatest temperature sensitivity (Figure 6g). This was followed by S12 with relatively lower *N_c_* and *N_t_* thus requiring a higher *T*_s_ of 40 °C to switch *E_a_*, and subsequently by S10 (Figure 6f). Finally, the continuous increase in bulk conductivity during heating across all samples was primarily due to the elevated kinetic energy of charge carriers at increased temperatures, with a shift in charge transport difficulty reflected by activation energy switching.

### 2.7 Meyer–Neldel Rule (MNR) in CEMe

**Figure 7:**
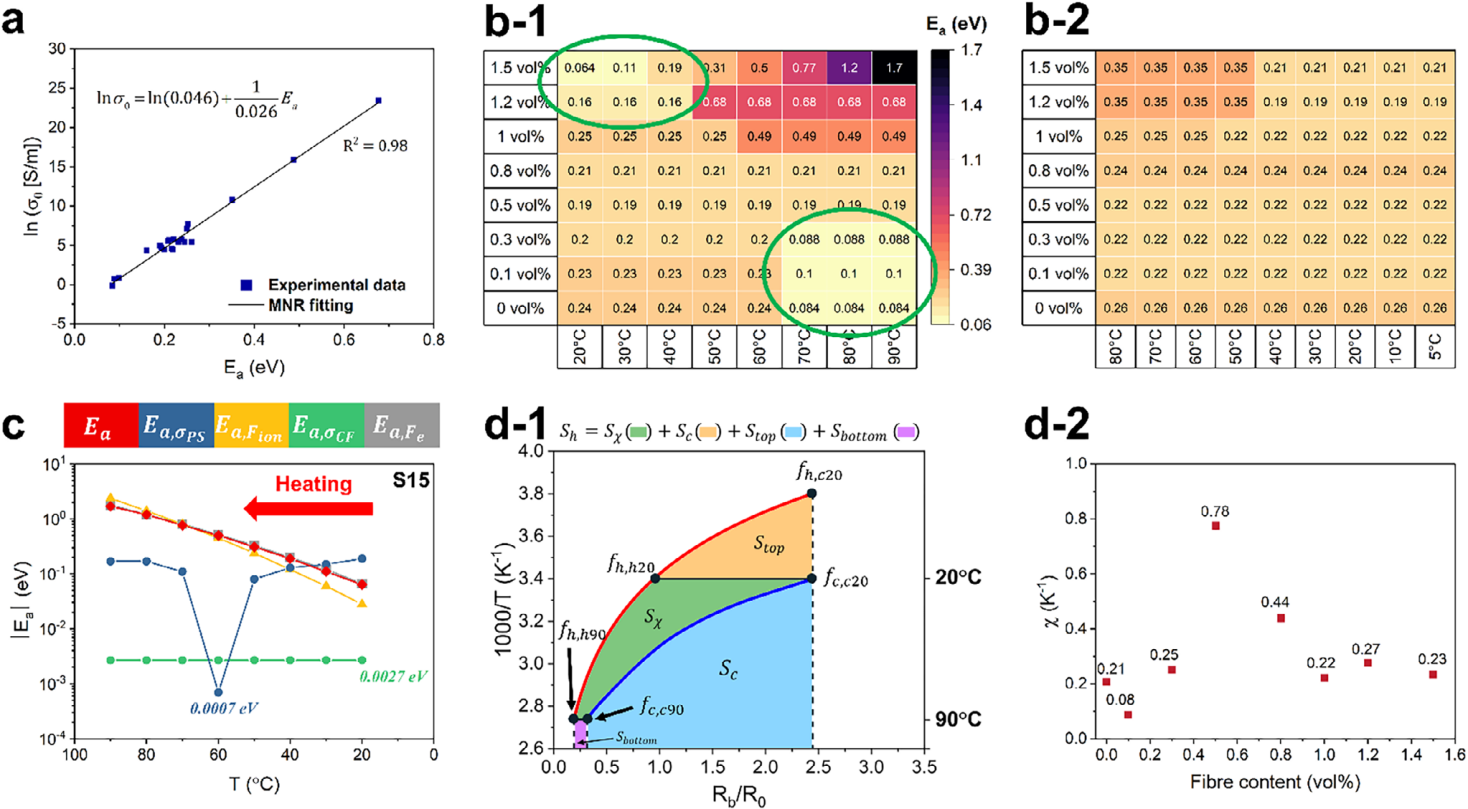
Compliance with Meyer–Neldel Rule and engineering remarks of CEMe. a) Compliance of MNR for samples obeying Arrhenius law. b‐1) Activation energy heat map for charge transport easiness during heating. b‐2) Activation energy heat map for charge transport easiness during cooling. c) Energy diagram of S15 during heating. Figure legends: *E_a_* the effective activation energy of bulk conductivity in red, $E_{a,\sigma_{PS}}$ the activation energy of intrinsic conductivity of pore solution in deep blue, $E_{a,F_{ion}}$ the activation energy for the opening process of the pore network in yellow, $E_{a,\sigma_{CF}}$ the activation energy of the intrinsic conductivity of carbon fibre in green, $E_{a,F_{e}}$ the activation energy for the “disconnecting” process of the fibrous network in grey. d‐1) Steinhart–Hart formulation and area segmentation method for the quantification of the hysteresis effect of normalized bulk resistance between heating and cooling: *R_b_* is the bulk resistance, *R_o_* the bulk resistance at pristine condition, *S_h_* the area enclosed by the curve of heating (in red) and the horizontal axis, *S_c_* the area enclosed by the curve of cooling (in blue) and the horizontal axis, *S_top_* the area on the top by extending the fitting of heating until having the same $\frac{R_{b}}{R_{0}}$ as that of cooling, *S_bottom_* the area at the bottom. Complete physical meaning of figure annotations is recorded in detailed in Section S16, Supporting Information. d‐2) Environmental susceptibility χ (K^−1^) for the quantification of bulk resistance hysteresis for CEMe with fibre contents 0–1.5 vol%.

MNR has been proven powerful for designing crystalline (or amorphous) semiconductors or solid‐state ionic conductors as a phenomenological validation of Arrhenius activation energy–conductivity relationship.^[^ ^94^, ^95^, ^96^ ^]^ In this study, compliance with MNR was validated in CEMes and PM for the first time (**Figure** **7**a). All samples with Arrhenius activation energy switching behaviour obeyed MNR except for the non‐Arrhenius behaviour of S15 during heating (Figure 2d). σ_00_ had a value of 0.046 S m^−1^ and the Meyer–Neldel Energy *E_MN_* equalled to 0.026 eV, in turn, the isokinetic temperature *T_iso_* was obtained to be 298.87 K (25.7 °C) which is near the sample's pristine temperature at laboratory condition (Section S10, Supporting Information). The MNR, also termed as the “compensation law”,^[^ ^97^ ^]^ originates from materials systems with inhomogeneity, disorder, and defects as the most prevalent properties,^[^ ^98^ ^]^ where “assistive activation energy” can be generated as a consequence of thermally dependent conductivity to compensation for the originally activated system.^[^ ^97^, ^99^, ^100^ ^]^ Analogously, the physical origin of MNR in CEMe, a biphasic conducting system with pronounced microstructure disorder and composition inhomogeneity, can be interpreted from a composite and macroscopic point of view. The compliance with MNR following Equation S25 (Supporting Information) suggests a linear relation between ln σ_0_ and activation energy *E_a_*, which holds persistently regardless of the fibre content and the receiving/dissipation of heat energy (i.e., heating or cooling) under the premise of the Arrhenius law. This implies that a component in CEMe exists which governs such persistent behaviour, whose chemical composition, phase state, microstructure, and conducting mechanism can maintain stable against the changes in fibre content and variation in heat transfer state. Such presence must be dominant (in the scale of either volume or mass) amongst all comprising components in CEMe.

The C─S─H gel, conducts electricity ionically and dominates a hardened cement matrix in terms of volumetric scale, morphological complexity, and chemical composition,^[^ ^101^, ^102^, ^103^, ^104^ ^]^ as well as being chemically stable over the measured temperature range (according to thermodynamic modelling, Figure 3a‐1). It has been validated that the relative conductivity of C─S─H gel (ratio of intrinsic electrical conductivity of C─S─H gel solids over that of pore solution) is between 0.0025 to 0.0078 under room temperature^[^ ^105^, ^106^, ^107^ ^]^ depending on the mix design. Applying the room temperature conditions, the *E_MN_* (0.026 eV) and *T_iso_* (298.87 K) derived in this study, were very close to the condition in which the standard thermal energy is 0.026 eV at an ambient temperature of 300 K. Therefore, as described by Equations S26,S27 (Supporting Information), under a thermal energy of *E_MN_*, the bulk conductivity σ is independent of the activation energy *E_a_* and equals to σ_00_. In this study, the intrinsic electrical conductivity of pore solution at room temperature was thermodynamically modelled to be 19 S m^−1^ (Figure 3d‐1). Consequently, by the ratio of σ_00_ (0.046 S m^−1^) over intrinsic electrical conductivity of pore solution, a relative conductivity of 0.0024 can be obtained, which is close to those estimated in the literature. Therefore, the physical meaning of MNR for CEMes and PM with Arrhenius behaviour is that the intrinsic conductivity of C─S─H gel in this study is 0.046 S m^−1^ under a room temperature of 298.87 K with a thermal energy of 0.026 eV, which maintained stable under changing fibre content and temperature (i.e., 5–90 °C), regardless of heating or cooling. Suggested interpretation on the disobedience of MNR for S15 was possibly that the incorporation of the percolated amount of fibre content had damaged the thermal stability of C─S─H gel (mainly through fibre surface bonding water molecules for a reduced available water for hydration^[^ ^73^ ^]^).

## 3 Engineering Remarks

In this section, we provide a straightforward performance evaluation on CEMe under the influence of two major variables in this study: fibre content and heat transfer condition. The percolation state can be controlled by purposefully controlling the fibre content whilst the heat transfer condition is an inevitable environmental influence. Charge transport performance, materials degradation process, and environmental susceptibility were evaluated and quantified using data from the above scientific findings and analyses. The findings presented here can serve as engineering reference toolbox for designing CEMe tailored to specific functionalities and service conditions, and for estimating degradation of conduction networks under certain service conditions, without the need for extensive experiments.

**(1) Charge transport heatmap**

The activation energy heatmap here can be used to predict CEMe's charge transport state under changing temperature and fibre content. During heating, high temperature facilitates conduction for the PM, as well as S1 and S3 due to their ionically dominant nature by the pore network. Whilst high temperature induces detrimental effects making charge transport more difficult for S10, S12, and S15 due to their electrically dominant nature by the fibrous network (Figure 7b‐1). During cooling, the charge transport state was relatively stable in comparison to that during heating (Figure 7b‐2). A relatively lower fibre content (preferably 0.3 vol%) is recommended for easier charge transport if facing frequent high‐temperature service conditions, whilst a higher fibre content (preferably 1.2 vol%) is recommended for easier charge transport if facing a regular and service condition with narrow temperature variation (marked in green circles).

**(2) Materials degradation energy diagram**

Figure 7c can be used as a reference diagram to estimate the sequential degradation process of the conduction pathways (i.e., pore network and fibrous network), as well as identifying potential microstructural flaws in CEMe at certain temperature (i.e., S15 with the most complex non‐Arrhenius behaviour as an example, complete energy diagrams for all the other samples are recorded in Section S14.3, Supporting Information). At the same temperature, lower |*E_a_* | means an easier change in property. At 20–30 °C, $|E_{a,\sigma_{CF}}|$ < $|E_{a,F_{ion}}|$ < $|E_{a,F_{e}}|$ < $|E_{a,\sigma_{PS}}|$, which means the increase in carbon fibre intrinsic conductivity was the most susceptibility to temperature, followed by the opening process of pore network, then the disconnecting process (i.e., decrease in *N_c_* and increase in *N_t_*) of fibrous network, and finally the increase in pore solution conductivity. Above 50 °C, both the opening of pore network and the disconnecting of fibrous network had higher activation energy hence developing less intensively than the changes in pore solution and carbon fibre conductivity. 60 °C is a temperature where the ion transport in pore solution had a lower energy barrier than the electrons/holes transport in fibre body (i.e., $|E_{a,\sigma_{CF}}|\;$= 0.0027 > $|E_{a,\sigma_{PS}}|$ = 0.0007 eV). Comparing in between the non‐Arrhenius behaviours of $|E_{a,F_{ion}}|$ and $|E_{a,F_{e}}|$, it can be seen that at 20–60 °C the opening of pore network was more pronounced than the disconnecting of the fibre network, reaching an equivalent at 70 °C, after which the disconnecting of the fibre network prevailed the opening of the pore network at 80–90 °C. It is proposed that 60 °C is a temperature where the PM and CEMes are the most susceptible to the chemical attacks (i.e., chloride/sulfuric ingressions) due to having an interionic environment of the lowest energy barrier for ion migration. Furthermore, it is proposed that the non‐Arrhenius behaviour is merely an algebraic manifestation of activation energy switching (or variable activation energy) behaviour with close *T*_s_ intervals at each step where the conduction pathway was remarkably sensitive to the temperature.

**(3) Environmental susceptibility**

The above scientific findings suggest that the hysteresis of conductivity and activation energy between heating and cooling (Figure 2) is inevitable under in‐service conditions due to irreversible materials thermal degradation. Nevertheless, quantifying the hysteresis can help to understand the thermal stability of conductivity in CEMes with different fibre contents, which can be a designing reference for targeted environmental conditions by only adjusting the fibre content without the need for additional physico‐chemical modifications. In Figure 7d‐1, Steinhart–Hart formulation in conjunction with the area segmentation method^[^ ^108^ ^]^ was employed for quantifying the environmental susceptibility of CEMe between 20–90 °C by calculating the area enclosed by the heating and cooling curves *S*_χ_, which can enlarge if CEMe is more susceptible to the environment and vice versa. Detailed solving process and methodologies are presented in Section S16 (Supporting Information), which is recommended for being embedded into the automation algorithm for further real‐time environmental susceptibility evaluation of CEMe. The results show S5 and S8 had the highest χ indicating the most unstable conductivity performance under thermal cycle, S1 had the lowest χ, meanwhile S3, S10, S12, and S15 shared a close χ value of ≈0.2 K^−1^ (Figure 7d‐2).

From the above engineering evaluations, it is obvious that through the percolation process of carbon fibre in cement matrix, the conducting nature transits from ionically dominant to electronically dominant, and different fibre range exhibits distinctive charge transport and susceptibility advantage under temperature varying conditions. **Table** **1** summarizes the preferred fibre content for targeted in‐service conditions (S10 was excluded due to poor charge transport performance during thermal cycle). If under in‐service condition with narrow and frequent temperature variation, S1, S3, S12, and S15 are preferable, whilst if under wide and infrequent temperature variation condition, S5 and S8 are preferable.

**Table 1:** Engineering look‐up table for designing CEMe by tuning fibre contents for the suggested in‐service conditions.

		Suggested in‐service condition	
CEMe	Fibre content [vol%]	Temperature range [°C]	Temperature variation frequency
S1	0.1	60—90	High
S3	0.3	60—90	High
S5	0.5	20—90	Low
S8	0.8	20—90	Low
S12	1.2	20—40	High
S15	1.5	20—40	High

## 4 Conclusions and Outlook

This study provides the first systematic investigation into the physical origin of activation energy switching and non‐Arrhenius behaviour in the bulk conductivity of plain mortar (PM) and electrically conductive cement (CEMe) under thermal cycling between 5 and 90 °C. The results reveal a percolation‐governed, counter‐competing mechanism between ionic and electronic conductive pathways. At low fibre contents (≤0.3 vol%), pore network opening dominates switching; at intermediate fibre contents (0.5–0.8 vol%), network effects balance and suppress switching; and at high fibre contents (≥1 vol%), fibrous network disconnection prevails. Standardized activation energies at ambient temperature were quantified, including 0.24 eV for effective ionic conduction in plain mortar and 0.064 eV for electronic contact conduction of fibre‐percolated CEMe, with 60 °C identified as the most degradation‐susceptible temperature. For the first time, compliance with Meyer–Neldel Rule was observed in CEMe, attributed to the thermal stability, volumetric dominance, and topological complexity of C─S─H gel phase.

Scientific findings in this study redefine activation energy switching as a universal indicator of conduction pathway degradation and establish a theoretical foundation for designing CEMes with tailored thermal‐electrical performance for multifunctional purposes. Engineering remarks in this study offer a substantive step forward for predictive control over durability and function, as well as the development of automation algorithms for real‐time performance monitoring of CEMe under in‐service conditions. The mechanistic framework developed here can be transferred to other multifunctional material systems with biphasic conducting networks where ionic–electronic competition governs charge transport (i.e., self‐sensing concrete, energy storage concrete, photocatalytic geopolymers, polymer–cement hybrids, etc.). While the present study is directly applicable to service conditions typical of moderately warm and humid climates (e.g., Bath, Shanghai, and other regions with comparable environmental characteristics), it offers a rigorous and transferable foundation for assessing the conduction behaviour of CEMe under more extreme service conditions, including frequent freeze–thaw cycles, prolonged drying, intense solar radiation, and repeated chemical attacks. This study provides a scientific basis for thermally activated charge transport physics in biphasic conducting materials, whilst advancing electrically conductive cement for multifunctional purposes in next‐generation infrastructure.

## 5 Experimental Section

CEMes were prepared by the incorporation of Polyacrylonitrile (PAN‐based) chopped carbon fibres (i.e., 6 mm in length and 7.5 µm in diameter) into the OPC mortar matrix. The dosages of carbon fibres were configured at 0, 0.1, 0.3, 0.5, 0.8, 1, 1.2, and 1.5 percentage by total volume (vol%), which were termed as PM (i.e., abbrev. plain mortar), S1 (i.e., abbrev. sample with 0.1 vol% of carbon fibres inclusion), S3, S5, S8, S10, S12, and S15, respectively. Thermally activated behaviour of electrical conductivity of the cementitious sample was characterized by controlling the temperature of the sample in a climate chamber (i.e., one thermal cycle with heating from 20 to 90 °C followed by cooling to 5 °C). The electrical impedance response was recorded via EIS, and the activation energy was extracted through the Arrhenius approach. Thermodynamic modelling was conducted on open‐source software GEM‐Selektor v.3 (http://gems.web.psi.ch/GEMS3/). Microstructural characterization was achieved via MIP and SEM – BSE. Compositional analysis was completed via XRF and EDX. Detailed fabrication, testing configuration, microstructural characterization, thermodynamic modelling methodology, and mathematical deduction processes are all specified in the Supporting Information.

## Conflict of Interest

The authors declare no conflict of interest.

## Supporting information



Supporting Information

## Data Availability

All data created during this research will be made available from the University of Bath Research Data Archive at: Zhang, J., ***2025***. *Data set for “Physical Origin of Temperature Induced Activation Energy Switching in Electrically Conductive Cement”*. Bath: University of Bath Research Data Archive. https://doi.org/10.15125/BATH‐01577 under the consent of all corresponding authors upon reasonable request.
