# Supplementary material for: Physical Origin of Temperature Induced Activation Energy Switching in Electrically Conductive Cement
Source: Adv Sci (Weinh). 2025 Nov 21:e17384. Online ahead of print. doi: 10.1002/advs.202517384 (PMC13325511; doi:10.1002/advs.202517384)
Supplement: Supplementary file 1 — Supporting Information [file ADVS-9999-e17384-s001.docx]

Supporting Information for

Physical Origin of Temperature Induced Activation Energy Switching in Electrically Conductive Cement

Jiacheng Zhang^a, b^, Xinyuan Ke ^b^*, Andrew Heath^b^*, Richard J Ball^b^, Jiawei Xie^a^, Jinchen Fan^a^, Guisheng Li^a^*, and Kevin Paine^b^

^a^ School of Materials and Chemistry, University of Shanghai for Science and Technology, Shanghai 200093, P.R. China

^b^ Department of Architecture and Civil Engineering, University of Bath, Claverton Down, BA2 7AY, Bath, United Kingdom

Corresponding authors

[X.Ke@bath.ac.uk](mailto:X.Ke@bath.ac.uk) (X. Ke), [A.Heath@bath.ac.uk](mailto:A.Heath@bath.ac.uk) (A. Heath), [liguisheng@usst.edu.cn](mailto:liguisheng@usst.edu.cn) (G. Li)

This file includes:

1. Supporting information statement

2. Nomenclature

3. Table of contents

4. Supporting information text

5. Figures S1 to S24

6. Tables S1 to S8

7. Equations S1 to S41

8. SI References

Supporting Information Statement

Supporting Information is available from the Wiley Online Library, from the author, or from the University of Bath library repository. To offer necessary protection and respect on the intellectual assets, this document is protected in ***University of Bath Research Data Archive*** with a unique DOI: <https://doi.org/10.15125/BATH-01577>. Citation of this Supporting Information is mandatory if referring to any originally developed methods in this study.

Nomenclature

| **Notation** | **Physical meaning** | **Unit** |
| --- | --- | --- |
| $I$ (or IS) | Ionic strength | mol/L |
| $c_{i}$ | Ionic concentration | mol/L |
| $z_{i}$ | Charge of ion | / |
| $\gamma_{CH}$ | Mean activity coefficient of Ca(OH)_2_ | / |
| $M$ | Coefficient | / |
| $Q$ | Coefficient | / |
| $z_{C}$ | Charge of Ca^2+^ | / |
| $z_{H}$ | Charge of OH^-^ | / |
| $\rho$ | Coefficient | / |
| $g$ | Coefficient | / |
| $K_{sp}$ | Solubility product | mol/L |
| $Z\left( \omega\right)$ | Impedance magnitude | Ω |
| $Z'\left( \omega\right)$ | Real impedance | Ω |
| $Z''\left( \omega\right)$ | Imaginary impedance | Ω |
| $\omega$ | Angular frequency | rad/s |
| $j$ | Operator $\sqrt{-1}$ | / |
| $\sigma\left( \omega\right)$ | Intrinsic conductivity | S/m |
| R_b_ | Bulk resistance | Ω |
| $k$ | Geometric factor | m |
| $\varepsilon_{r}(\omega)$ | Relative permittivity | / |
| $\varepsilon_{0}$ | Absolute permittivity of vacuum | F/m |
| $tan\delta(\omega)$ | Loss tangent | / |
| $\varepsilon"(\omega)$ | Imaginary permittivity | F/m |
| $\varepsilon'(\omega)$ | Real permittivity | F/m |
| $\sigma_{PS}$ | Intrinsic conductivity of pore solution | S/m |
| $F$ | Faraday’s constant | J/mol |
| $R$ | Ideal gas constant | J/(K·mol) |
| $T$ | Temperature | K or °C |
| $D_{i}(T)$ | Diffusion coefficient | m^2^/s |
| $\gamma_{i}(T)$ | Ionic activity coefficient | / |
| $\alpha_{i}(T)$ | Activity coefficient correction factor | / |
| $k_{B}$ | Boltzmann constant | eV/K |
| $\eta(T)$ | dynamic viscosity | g/(m·s) |
| $r_{i}$ | Stokes radius | m |
| $\sigma$ | Intrinsic bulk conductivity | S/m |
| $\sigma_{0}$ | Arrhenius pre-exponential factor | S/m |
| $e$ | Natural base | / |
| $E_{a}$ | Effective activation energy of bulk electrical conductivity | eV |
| $d_{1}$, $d_{2}$, and $d_{3}$ | Coefficients | / |
| $\sigma_{{OH}^{-}}$ | Conductivity by OH^-^ ion migration | S/m |
| $A_{poly}$ | Coefficient | / |
| $B_{i}$ | Coefficient | / |
| $\sigma_{00}$ | MNR pre-exponential factor | S/m |
| $E_{MN}$ | Meyer–Neldel energy | eV |
| $T_{iso}$ | isokinetic temperature | K or °C |
| $\tau$ | Pore tortuosity | / |
| $\alpha_{en}$ | Pore entrapment fraction | / |
| $D_{s}$ | Pore fractal dimension | / |
| $N_{s}$ | Number of copies of the original geometry | / |
| $R_{s}$ | Iiteration of scaling | / |
| $S$ | Pore surface area | m^2^ |
| $V$ | Pore volume | m^3^ |
| $W_{n}$ | Cumulative work down by mercury during intrusion | N·m |
| $r_{n}$ | Pore radius | m |
| $V_{n}$ | Intruded pore volume | m^3^ |
| $C_{s}$ | Coefficient | / |
| $P_{i}$ | Applied pressure | N/m^2^ |
| $\Delta V_{i}$ | Increased pore intrusion volume | m^3^ |
| $S_{h}$ | Area under heating SH curve | K^-1^ |
| $S_{c}$ | Area under cooling SH curve | K^-1^ |
| $S_{top}$ | Area at the top of heating SH curve | K^-1^ |
| $S_{bottom}$ | Area at the bottom of heating SH curve | K^-1^ |
| $S_{\chi}$ | Area for quantifying environmental susceptibility | K^-1^ |
| $f_{h}$ | SH heating fitting function | / |
| $f_{c}$ | SH cooling fitting function | / |
| $R_{0}$ | Bulk resistance at 20°C pristine condition | Ω |
| $R_{h,90}$ | Bulk resistance at 90°C during heating | Ω |
| $R_{c,20}$ | Bulk resistance at 20°C during cooling | Ω |
| $y_{20}$ | Value of $\frac{1000}{T}$ at 20°C | K^-1^ |
| $y_{90}$ | Value of $\frac{1000}{T}$ at 90°C | K^-1^ |
| $A_{s}$, $B_{s}$, and $C_{s}$ | Coefficients | K^-1^ |
| $A_{h}$, $B_{h}$, and $C_{h}$ | Coefficients | K^-1^ |
| $A_{c}$, $B_{c}$, and $C_{c}$ | Coefficients | K^-1^ |
| $\chi$ | Environmental susceptibility | K^-1^ |

*$i$ and $n$ are numbering utilities.

Table of contents

[SI – 1. Materials and mix design 6](#_Toc212135936)

[SI – 2. Fabrication 8](#_Toc212135937)

[SI – 2.1. Plain mortar and electrically conductive cement 8](#_Toc212135938)

[SI – 2.2. Simulated pore solution 10](#_Toc212135939)

[SI – 3. Thermal cycle configurations 12](#_Toc212135940)

[SI – 3.1. Plain mortar and electrically conductive cement 12](#_Toc212135941)

[SI - 3.2. Simulated pore solution 15](#_Toc212135942)

[SI – 4. Electrochemical impedance spectroscopy (EIS) 17](#_Toc212135943)

[SI – 5. Variation in moisture content during thermal cycle 18](#_Toc212135944)

[SI – 6. Chemical equilibria of simulated pore solution 19](#_Toc212135945)

[SI – 6.1. Determination programme 19](#_Toc212135946)

[SI – 6.2. Validation programme 21](#_Toc212135947)

[SI – 7. Thermodynamic modelling 21](#_Toc212135948)

[SI – 7.1. Methodology 21](#_Toc212135949)

[SI – 7.2. Modelling results for low-concentration species in pore solution 22](#_Toc212135950)

[SI – 7.3. Modelling results for variation of hydration products in the form of both volume and mass 23](#_Toc212135951)

[SI – 7.4. Modelling results for thermodynamic activity coefficient and correction factor 23](#_Toc212135952)

[SI – 8. Calculation of electrical parameters 24](#_Toc212135953)

[SI – 8.1. Intrinsic bulk conductivity, relative permittivity, and loss tangent (using as-measured experimental data) 24](#_Toc212135954)

[SI – 8.2. Intrinsic pore solution conductivity (using thermodynamic simulation data) 25](#_Toc212135955)

[SI – 9. Calculation of activation energies 26](#_Toc212135956)

[SI – 9.1. Arrhenius behaviour 26](#_Toc212135957)

[SI – 9.2. Non-Arrhenius behaviour 26](#_Toc212135958)

[SI – 10. Meyer–Neldel Rule (MNR) validation 28](#_Toc212135959)

[SI – 11. Microstructural characterization 29](#_Toc212135960)

[SI – 11.1. Backscattered electrons imaging (SEM – BSE) and Energy Dispersive X-Ray Spectroscopy (EDX) 29](#_Toc212135961)

[SI – 11.2. Mercury Intrusion porosimetry (MIP) 32](#_Toc212135962)

[SI – 12. Development of electrical conductivity through curing age 36](#_Toc212135963)

[SI – 12.1. Complex impedance of PM and CEMes at 97 days of curing within a fabrication batch 36](#_Toc212135964)

[SI – 12.2. Development of percolation process through curing age 38](#_Toc212135965)

[SI – 13. Effect of temperature and fibre content on impedance behaviours 38](#_Toc212135966)

[SI – 13.1. Complex impedance of PM and CEMe 38](#_Toc212135967)

[SI – 13.2. Complex impedance of simulated pore solution 41](#_Toc212135968)

[SI – 13.3. Loss tangent 41](#_Toc212135969)

[SI – 14. Thermally induced alteration in the architecture of conduction pathways 43](#_Toc212135970)

[SI – 14.1. Ionic conduction pathway (pore network) 43](#_Toc212135971)

[SI – 14.2. Electronic conduction pathway (fibrous network) 44](#_Toc212135972)

[SI – 14.3. Materials degradation energy diagrams 45](#_Toc212135973)

[SI – 15. Determination on ionic and electronic conductivity percentages 45](#_Toc212135974)

[SI – 16. Solving process of environmental susceptibility $\boldsymbol{\chi}$ 46](#_Toc212135975)

[SI – 17. SI References 50](#_Toc212135976)

Supporting Information Text

This supporting document provides detailed information on the experimental programme, microstructural characterization, thermodynamic modelling, and mathematical deductive approaches. All experimentally acquired and/or extracted results are included in this document.

1. Materials and mix design

Standard sand conforming to BS EN 196-1 (SNL, Leucate, France), CEM**Ⅰ**42.5R conforming to BS EN 197-1 (Dragon Alpha Cement Limited, Sharpness, UK), and deionized water were used as the raw materials for preparing the plain mortar (PM). Electrically conductive cements (CEMes) were prepared by admixing 0.1, 0.3, 0.5, 0.8, 1, 1.2, and 1.5 volume percentages (vol%) of polyacrylonitrile (PAN-based) chopped carbon fibres (SGL Carbon Fibers Ltd, Wiesbaden, Germany) into mortar matrix. These samples were termed as PM (plain mortar), S1, S3, S5, S8, S10, S12, and S15, respectively. Polycarboxylate superplasticizer (PERAMIN COMPAC, Upplands Vasby, Sweden) was used as dispersant for improving the dispersibility of carbon fibres in cementitious matrix as well as the workability of the wet mix at a content of 0.5% by mass of cement for each sample. Tributyl phosphate (Sigma-Aldrich, Dorset, UK) was employed as defoamer to reduce the air bubbles during mixing at a content of 3 mL for each fabrication batch. Sand to cement and water to cement ratios for each sample were fixed at 3:1 and 0.45:1, respectively. Reagent grade NaOH, KOH, Na_2_SO_4_, and Ca(OH)_2_ powders (SIGMA-ALDRICH, Dorset, UK), as well as deionized water were used to prepare the simulated pore solution (SPS), potassium hydroxide solution (KOH), and saturated calcium hydroxide solutions (SCH). The bulk chemicals of CEM**Ⅰ**42.5R are presented in Table S1. Detailed physical properties of carbon fibres used in this study were presented in Table S2. Temperature dependence of intrinsic conductivity for single carbon fibre filament used in this study was presented in Figure S1. Table S3 summarised the thermal expansion/shrinkage properties of solid and liquid components in a hardened CEMe.

1. XRF oxide analysis on CEMⅠ42.5R

| **Oxides** | **Value (wt%)** |
| --- | --- |
| CaO | 62.8 |
| SiO_2_ | 20.1 |
| Al_2_O_3_ | 5.4 |
| Fe_2_O_3_ | 3.0 |
| MgO | 2.2 |
| SO_3_ | 2.2 |
| K_2_O | 0.9 |
| P_2_O_5_ | 0.2 |
| TiO_2_ | 0.2 |
| Na_2_O | 0.1 |
| Mn_3_O_4_ | 0.1 |
| LOI | 3.4 |

1. Technical specifications of PAN-based chopped carbon fibres from manufacturer

| **Physical properties** | **Value** |
| --- | --- |
| Density (g • cm^-3^) | 1.8 |
| Fibre length (mm) | 6 |
| Filament diameter (μm) | 7.5 |
| Tensile strength (GPa) | 4 |
| Tensile modulus (GPa) | 240 |
| Elongation at break (%) | 1.7 |
| Sizing type | Glycerine |
| Electrical resistivity (ohm • m) | 1.5 × 10^-5^ |
| Coefficient of thermal expansion (CTE) (°C^-1^) | -0.7 × 10^-6^ |

1. Coefficient of Thermal Expansion (CTE) of solid and liquid components in hardened CEMe

| **Comprising components in CEMe** | **CTE × 10^-6^ (°C^-1^)** |
| --- | --- |
| Near-saturated cement paste | 10 – 15 [1] |
| Sand | 6 [1] |
| PAN-based carbon fibres | -0.7 (Table S2) |
| Water | 210 [2] |


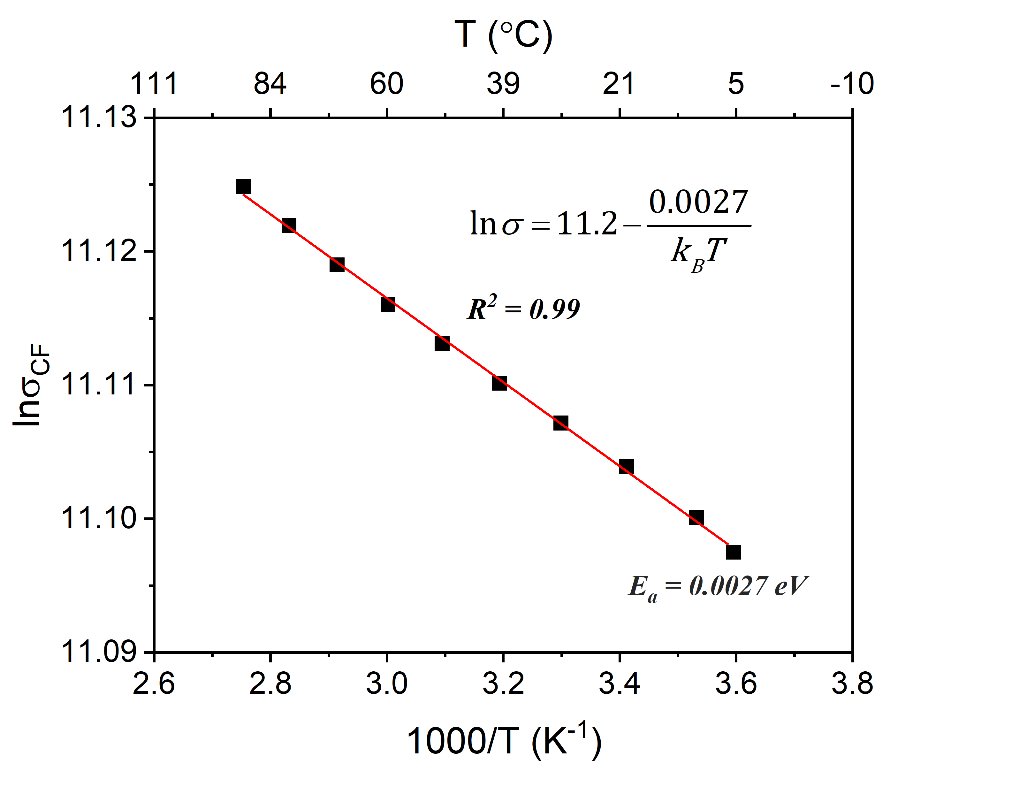


1. Temperature dependence of intrinsic conductivity of PAN-based carbon fibre in this study under thermal cycle 5 – 90 °C.
2. Fabrication

SI – 2.1. Plain mortar and electrically conductive cement

The standard procedures specified in BS EN 196-1 were followed for casting: the duration of blending carbon fibre with wet cement was extended and the sand was added into the wet mixture at last. Prior to the fabrication of CEMe, the carbon fibres were submerged in deionized water and put under ultrasonication at 45 °C for 30 min. This procedure was repeated for 3 times in order to clean the sizing off the carbon fibre surface. The cleaned carbon fibres were then put in 50 °C oven for drying until the mass reached equilibrium before further use. Before being mixed with cement to fabricate CEMe, the surfaces of the cleaned carbon fibres were functionalized by polycarboxylate superplasticizer to ensure strong hydrophilicity. Detailed fabrication processes of CEMe are presented below:

1. The whole portion of the polycarboxylate superplasticizer (i.e., abbrev. PCE, powdery form) was mixed with the whole portion of mixing water in a beaker in an ultrasonic bath (Shesto Ltd., Watford, UK) under 45°C for 10 min, during which half portion of the Tributyl phosphate (i.e., abbrev. TPH, translucent liquid form, 5 drops for 1.5 mL) was dropped into the mix and manually stirred with a glass stirrer (Figure S2a).
2. The whole portion of carbon fibre was added into the mix, then kept in bathed under ultrasonication at 45°C for 20 min. The remaining half portion of the TPH was dropped into the mix with manual stirring during the same time (Figure S2b).
3. The fibrous solution mixture was mixed with the whole portion of cement in a Digital Mortar Mixer (CONTROLS Ltd., Hertfordshire, UK) for 5 min at low speed (around 140 rpm). Subsequently, the whole portion of sand was added into the wet mixture and mixed for another 3 min at high-speed (around 285 rpm). Then, the mixer was stopped for less than 90 s for scraping off the mixture adhering to the bowl wall and bottom, as well as for manual homogenization. Afterwards, another 5-min of high-speed blending was employed on the mixture (Figure S2c).
4. The wet mixture was poured into a standard steel mould where three 40 × 40 × 160 mm prismatic samples conforming to BS EN 196-1 were cast. The wet mix was then compacted on a vibration table for 2 min (Figure S2d). The stainless-steel electrode array was inserted into the wet sample at the depth of 30 mm (Figure S2e).
5. Finally, the mould containing the wet samples were wrapped with airtight film to minimize moisture loss and carbonation (Figure S2e). The samples were demoulded after being settled under laboratory condition (i.e., 23 ± 2°C, 50%RH) for 24 hours (Figure S2f).


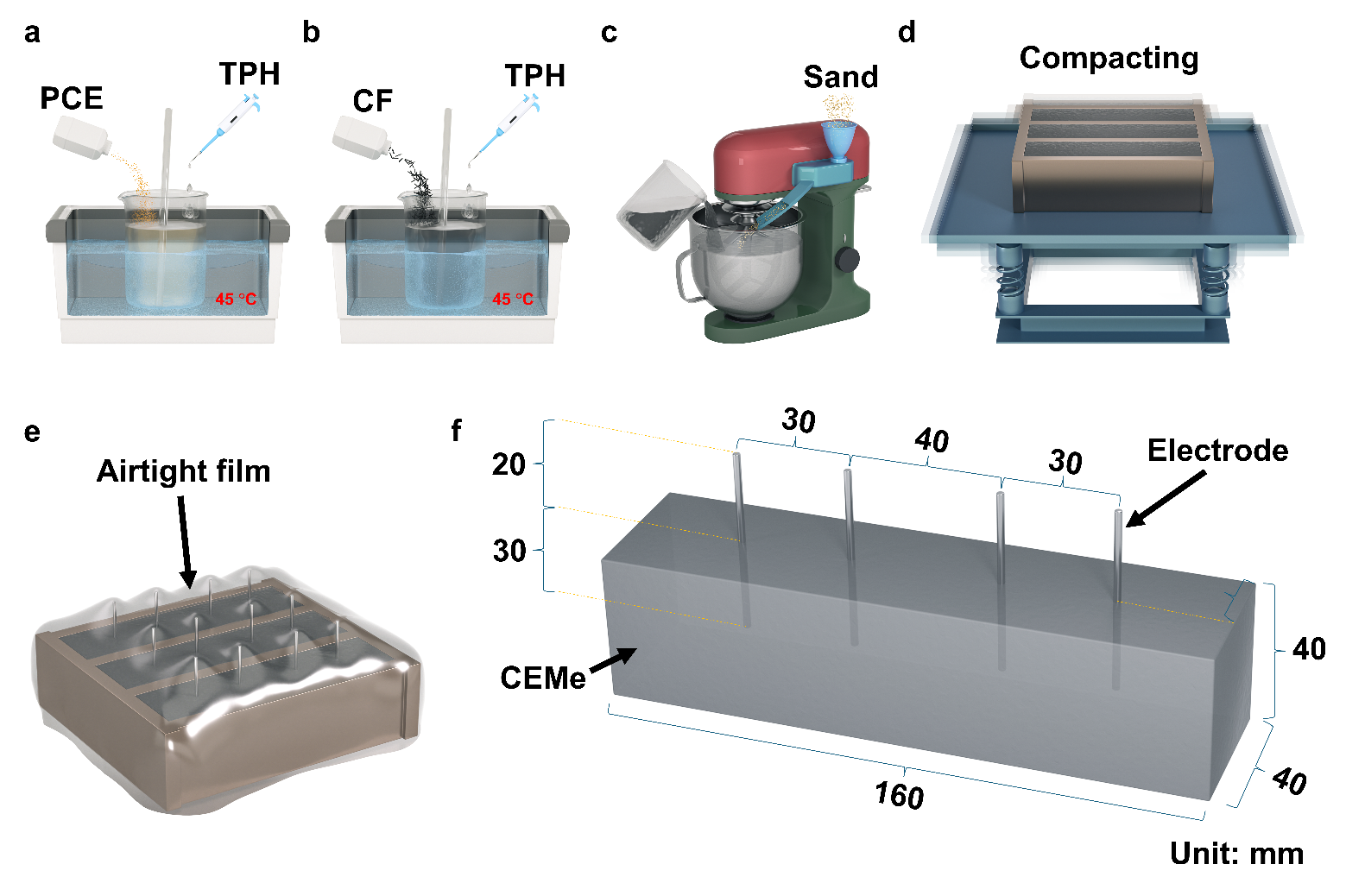


1. Schematic illustrations of the manufacturing process of electrically conductive cement (three standard prismatic samples were produced within one batch for each fibre content as specified in BS EN 196-1): PCE is polycarboxylate superplasticizer, TPH is tributyl phosphate defoamer, and CF is carbon fibre.

There were three samples made within one fabrication batch for each fibre content. Two samples were crushed for microstructural characterizations (Section SI – 11, Supporting Information). The remaining one sample was used for testing under thermal cycle which was termed as testing sample (Section SI – 3, Supporting Information), which was further fractured for the characterization of microstructural conditions after thermal cycle (Section SI – 11, Supporting Information). The low variation in impedance response and the consistent development of the conductivity percolation trend at different curing ages (as presented in Section SI – 12) indicate that the fabrication procedure and the dosage of PCE superplasticizer are appropriate. These factors ensure a high quality of fibre dispersion, as well as a consistent development of the conduction state amongst the three samples within a fabrication batch. Hence, the thermal cycling results are expected to be of high quality, reliability, and reproducibility.

SI – 2.2. Simulated pore solution

Simulated pore solution was prepared following the chemical equilibria determined by the NIST’s data [3–5] and the Moragues et al.’s methods [6,7] (Section SI – 6, Supporting Information). Detailed synthesis process is presented as follows:

1. 0.825g of Ca(OH)_2_ powder was added into 500 mL deionized water in a beaker with gentle manual stirring.
2. 12.6g of KOH, 0.852g of Na_2_SO_4_, and 1.32g of NaOH powders were added into the solution with gentle manual stirring. At this step more solids were precipitated due to the reaction between dissolved Ca(OH)_2_ and Na_2_SO_4_, producing CaSO_4_
3. Another 0.825g of Ca(OH)_2_ powder was added into the solution with gentle manual stirring to reach a stable supersaturation of Ca(OH)_2_ forming a white suspension (Figure S3a). The appearance is very close to a curing water without changing at a long enough time.
4. After a 3-min manual stirring, to minimize carbonation, the suspension was immediately transferred for vacuum filtration (Figure S3b). A seemingly translucent liquid (light transmissible but marginally cloudy) containing ionic species Na^+^, K^+^, Ca^2+^, OH^-^, and SO_4_^2-^ was obtained (Figure S3c) for further testing. These five ions are also the major free ions contributing to bulk ionic conduction process in CEMe.


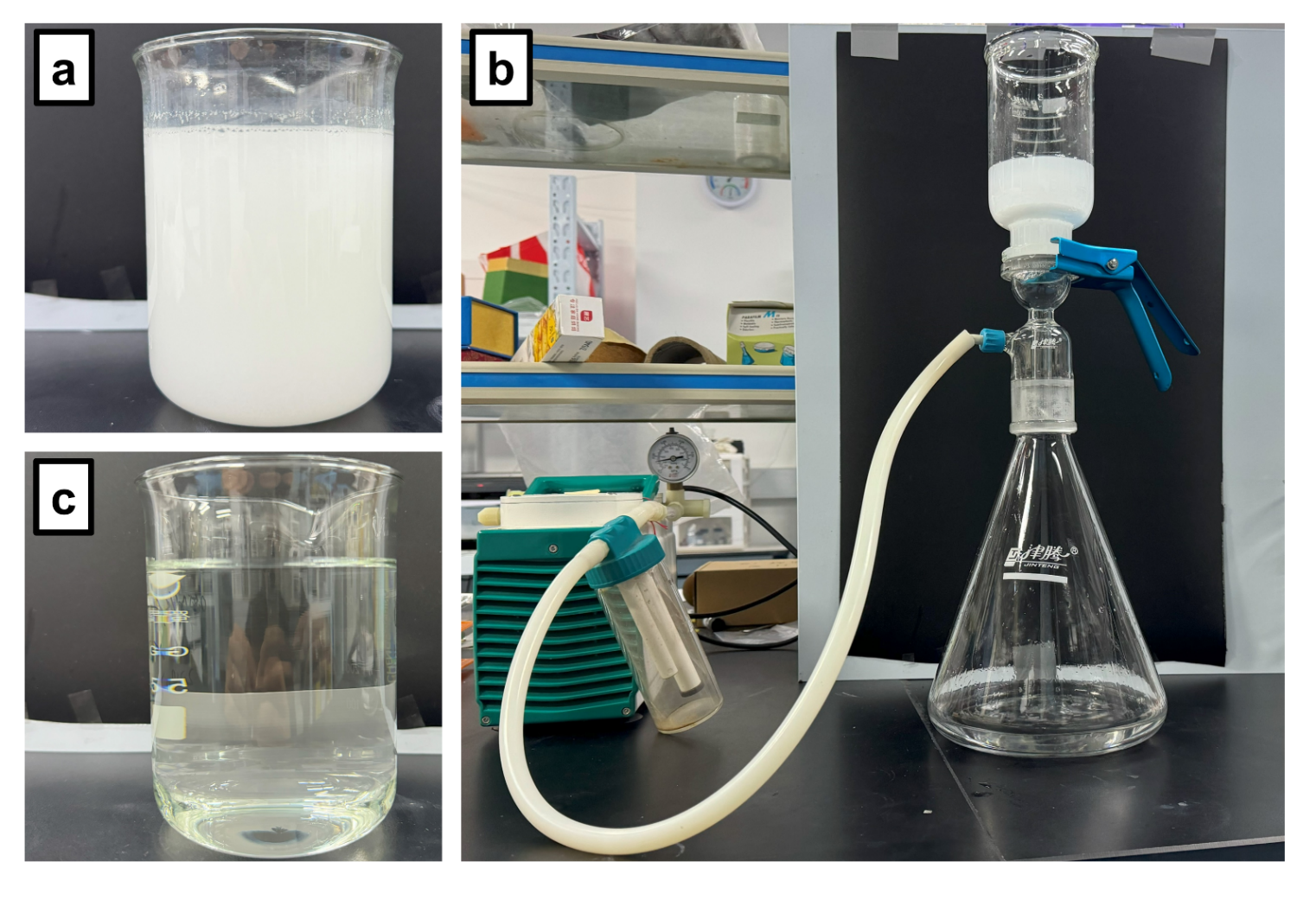


1. Synthesis of simulated pore solution. a) as-prepared simulated pore solution in Step 3. b) vacuum filtration for separation of liquid phase and solid precipitations. c) appearance of simulated pore solution after filtration in Step 4.

Potassium hydroxide solution (0.54 mol/L) and saturated calcium hydroxide solution (0.022 mol/L) were also prepared for referenced behaviours during thermal cycle under the same protocol. The pH values of all as-prepared solutions were tested using a SevenExcellence S400 pH metre (Mettler-Toledo Ltd., Leicester, UK). The final molar concentrations of ionic species in simulated pore solution, potassium hydroxide solution, and saturated calcium hydroxide solutions are presented in Table S4 along with their as-prepared pH values and calculated ionic strength (IS). Detailed determination of their chemical equilibria is presented in Section SI – 6, Supporting Information. It can be seen that the concentration of Ca^2+^ in simulated pore solution was only about half of that in the binary saturated calcium hydroxide solution, which is due to high concentrations of the alkalis. Dependence of solubility on temperature between 5 – 90°C was positive for NaOH and KOH, negative for Ca(OH)_2_, and piecewise for CaSO_4_ (Table S5).

1. Molar concentration of free ions in simulated pore solution (SPS), potassium hydroxide solution (KOH), and saturated calcium hydroxide solution (SCH), as well as the measured pH values and Ionic strength (IS).

| **Free ions** | **Molar concentration (mol/L)** | | | | |
| --- | --- | --- | --- | --- | --- |
|  | **SPS** | | **KOH** | | **SCH** |
| Na^+^ | 0.090 | / | | / | |
| K^+^ | 0.450 | 0.540 | | / | |
| Ca^2+^ | 0.012 | / | | 0.022 | |
| SO_4_^2-^ | 0.012 | / | | / | |
| OH^-^ | 0.540 | 0.540 | | 0.044 | |
| pH at 25°C | 13.58 | 13.55 | | 12.56 | |
| IS (mol/L) at 25°C | 0.590 | 0.540 | | 0.066 | |

1. Molar solubility of chemical compounds in water considering only the binary form for a reference

| **Chemical**  **compounds** | **Molar solubility**  **(mol/L) at 25°C** | **Dependence of solubility**  **on temperature (5 – 90°C)** |
| --- | --- | --- |
| NaOH | 25.000 | Positive |
| KOH | 21.570 | Positive |
| Ca(OH)_2_ | 0.022 | Negative |
| CaSO_4_ | 0.015 | Positive at 0 – 40°C, negative at 40 – 90°C [8] |

1. Ionic mobility of the five major free ions in OPC pore solution at infinite dilution at 25°C.

| **Free ions** | **Ionic mobility (10^-8^ m^2^/V⋅s)** |
| --- | --- |
| OH^-^ | 20.5 |
| SO_4_^2-^ | 8.3 |
| K^+^ | 7.6 |
| Ca^2+^ | 6.2 |
| Na^+^ | 5.2 |

1. Thermal cycle configurations

SI – 3.1. Plain mortar and electrically conductive cement

SI – 3.1.1. Curing, Preparation and thermal cycle operation

Upon demoulding, a T-type copper-nickel thermocouple conforming to BS EN IEC 60584 (RS Components Ltd, Bristol, UK) was employed at a depth of 3 mm from the surface of each sample using epoxy glue for further monitoring of sample surface temperature at thermal cycle (Figure S4a). Samples were then put in a water tank and cured under water (20 ± 3°C) for a period of 97 days which was suggested to be the period for OPC to be stabilized in terms of the development of pore structure and electrical conductivity of pore solution [9]. Upon finishing curing, samples were removed from the water tank and cleaned with paper towel on the surface. Each sample was then immediately wrapped with PE cling film for 20 – 30 layers and sealed with adhesive tape with only the electrodes exposed in order to minimize the moisture exchange between the sample and the surrounding atmosphere (Figure S4b). Two purposes were fulfilled for wrapping up the samples: 1) to simulate the realistic service condition where the CEMes were embedded with a certain depth into concrete structure where the moisture exchange between CEMe and outside environment was much slower than the surface layer hence the moisture variation is considered minimized and quasi-static [10]. 2) to preserve the moisture content as much as possible so that the temperature dependence of ionic conduction through pore solution in the pore network in CEMe matrix can be realized and characterized as accurately as possible.


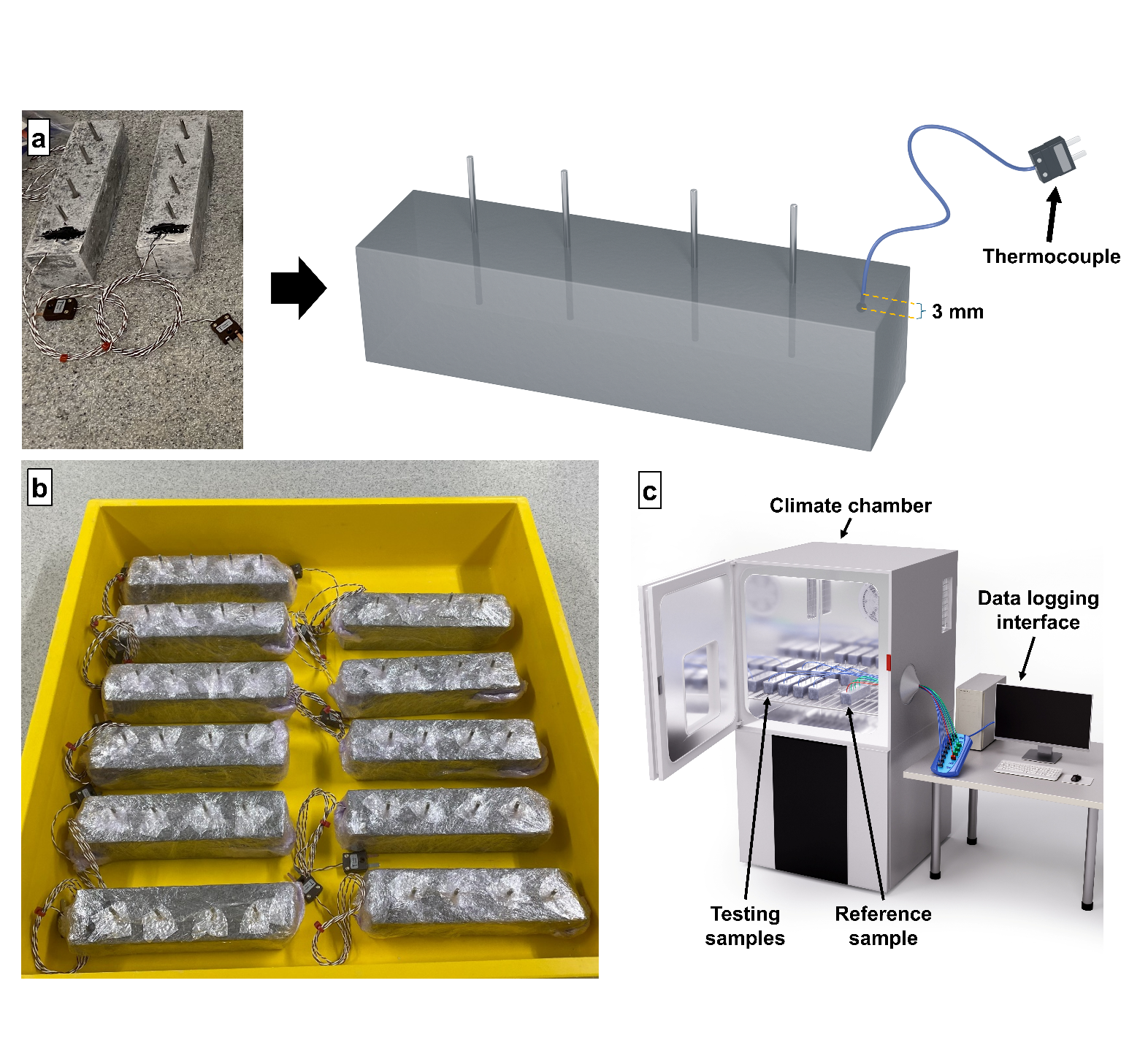


1. Preparation of samples for thermal cycle and detailed thermal cycle configurations. a) testing samples attached with thermocouples. b) testing samples sealed with PE film. c) thermal cycle configuration in climate chamber connected to temperature monitoring interface for real-time temperature monitoring.

After an initial measurement on mass and impedance response at pristine laboratory condition, samples were placed in the climate chamber for thermal cycle (Figure S4c). The RH in the chamber was fixed at 60% and the temperature was elevated progressively in a sequence of 30, 40, 50, 60, 70, 80, 90°C for heating, followed by a downgrading sequence of 80, 70, 60, 50, 40, 30, 20, 10, 5°C for cooling. The power of the climate chamber is 3 kW.

SI – 3.1.2. Temperature equilibrium monitoring

The impedance signal response can be largely influenced if embedding the metallic head of T-type thermocouple into sample bulk between the measuring electrodes. Therefore, only one thermocouple was attached on the surface of each sample with a distance away from the two inner measuring electrodes (Figure S2F and Figure S4A). In order to make sure the location between the electrodes, which was the sample geometric centre where the impedance was measured (Figure S7), had reached the temperature of interest, the duration for the temperatures of sample surface and bulk to reach equilibrium must be ascertained. Therefore, a dedicated reference PM from the same mixing batch as the testing PM was prepared (Figure S5a). Four thermocouples were deployed where three being embedded into the bulk with a depth of 20 mm and one being employed on the sample surface at the same location as the testing samples. The reference PM was then sealed with water resistant gel (Raytech, Settimo Milanese, Italy) and PE film in an acrylic box, and placed alongside with the testing samples in the climate chamber for thermal cycle (Figure S4c). The attached thermocouple on the surface of the testing sample was to ensure the consistency of temperature monitoring with the reference PM. The temperature was recorded through a data logging interface at a frequency of 0.017 Hz (Figure S4c).

The accuracy for temperature measurement was controlled within ±1°C of variation due to the accuracy range of the climate chamber. It was experimented that the duration for all bulk and surface thermocouples to reach temperature equilibrium was 210 minutes for a 10°C of increase during heating and 900 minutes for a 10°C of decrease during cooling (Figure S5b). It has also been evaluated that when the surface thermocouple (marked as surface in Figure S5a) reached the temperature of interest, it would take 60 and 700 minutes for all the bulk thermocouples (marked as bulk 1, 2, and 3 in Figure S5a) to reach the same temperature when heating and cooling, respectively (Figure S5b). When all thermocouples in reference PM and testing samples (Figure S4c) showed consistent reading reaching the temperature of interest as configured above, the testing samples were taken out of the climate chamber for the measurement of mass and impedance response. The duration of the measurements was limited within 30 seconds so that the impedance characteristics at the temperature of interest can be determined as accurate as possible. Through the above rationale, a real-time “temperature – impedance” correspondence can be reliably obtained.


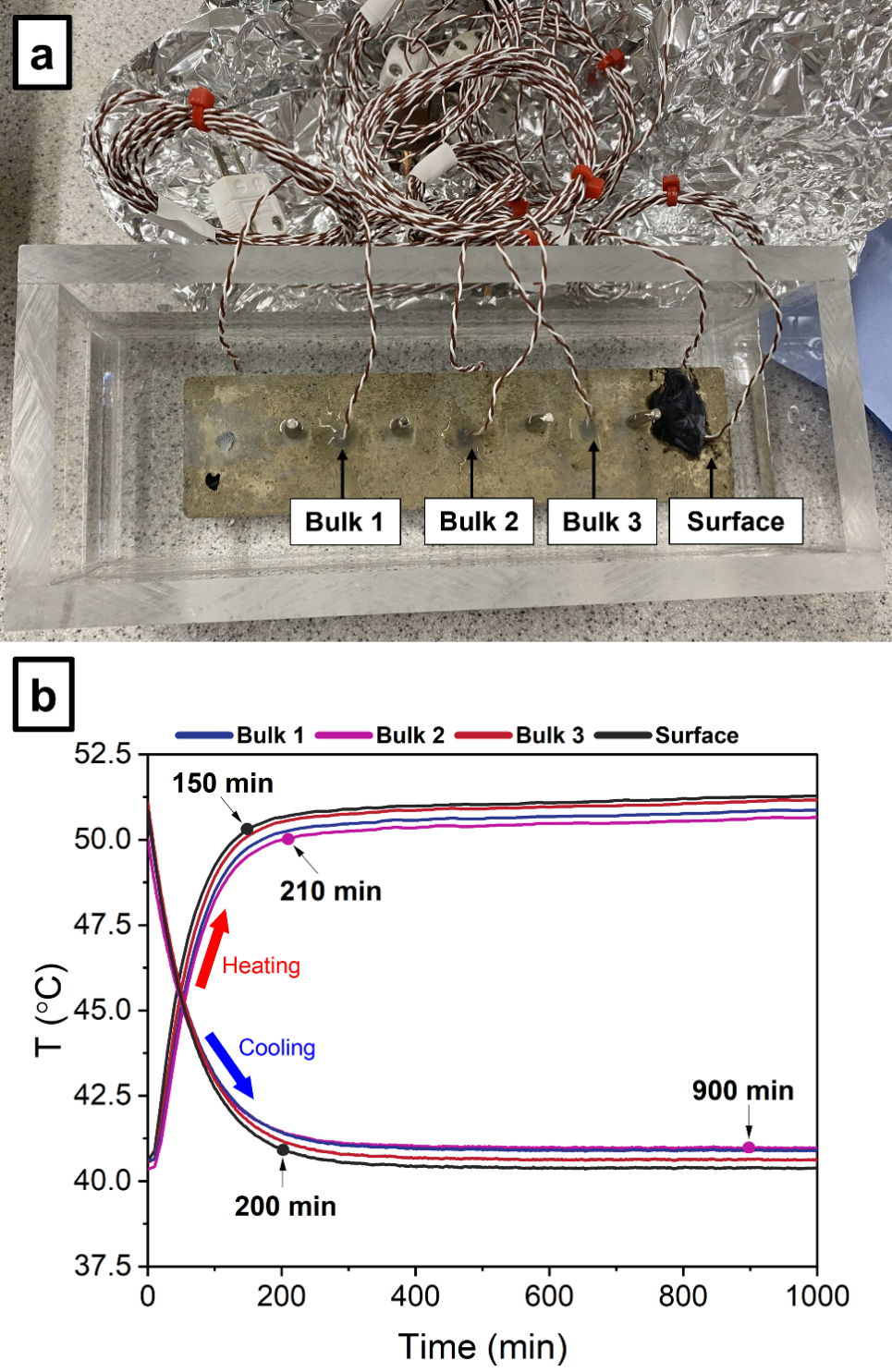


1. A reference PM for ascertaining the minimal duration to reach temperature equilibrium throughout sample body. a) location of the thermocouples in reference PM: 3 in the bulk and 1 near the surface (3mm of depth into the surface). b) duration to reach temperature equilibrium (the interval between 40 and 50°C as an example).

Finally, upon finishing the thermal cycle, the PE sealing was removed from each sample. Continuous drying was performed to determine the dry mass and the water saturation degree, following the experimental protocol in [11] and Section SI – 14, Supporting Information. The changes in moisture content during thermal cycle were recorded in Section SI – 5, Supporting Information.

SI - 3.2. Simulated pore solution

Heating and cooling cycle was immediately proceeded upon finishing preparing the three solutions to minimize carbonation. A testing cell was assembled where two stainless steel electrodes were fixed on the top of a beaker using an acrylic fixer and the acrylic fixer was glued to the beaker aligning with the centre of the top opening (Figure S6a). 100 mL of solution was transferred into the testing cell which was then immediately sealed with PE film to minimize carbonation. The distance between the electrodes was 25 mm and the electrodes were in contact with the solution with a depth of 15 mm from surface into the solution. The testing cell was then bathed in deionized water for thermal cycle and the whole water bath was again sealed with PE film for a more controlled heating process (Figure S6b). A Kerosene thermometer was penetrated through the PE film into the solution for real-time monitoring on the solution temperature and crocodile electrodes were employed for impedance measurement. Finally, the whole setup was placed on a hotplate stirrer platform (Joan Lab Equipment Ltd., Zhejiang, China) for heating and cooling process. The solution was homogenized with a magnetic stirrer bar with a rotational speed of 520 rpm throughout the entire thermal cycle (Figure S6c). This created a shear force in the solution which assisted the secondary nucleation process [12].


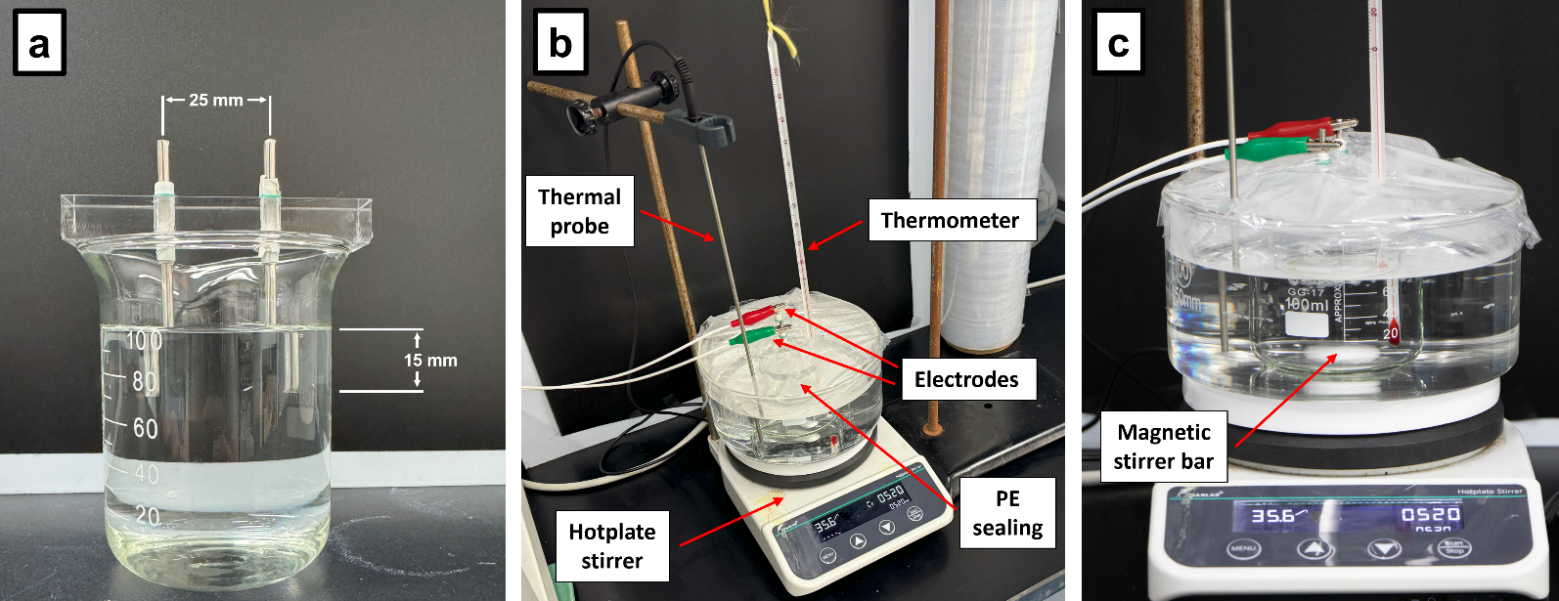


1. Heating and cooling configurations for testing the thermal dependence of the electrical conductivities of simulated pore solution, potassium hydroxide solution, saturated calcium hydroxide solution. a) testing cell dimension (with 100mL KOH solution). b) heating and cooling of the testing cell containing electrolytic solution in a water bath. c) magnified observation for the testing configuration.

During heating, the hotplate was configured at different temperatures to heat up the water bath and the solution was indirectly heated through the heated water bath. Impedance measurement was undertaken at pristine laboratory temperature (23 ± 2°C) and at as many temperatures as possible from 30 to 90°C including the temperatures as mentioned above for the cement samples. This is to gain a comprehensive understanding of the thermo-electrical properties of the electrolyte solutions because they are well ionic conductors and can be very sensitive to the temperature changes. During cooling, the PE sealing of water bath was removed but the PE sealing of the testing cell was kept in place to minimize carbonation. The heating was stopped, and the solution was naturally cooled from 90°C to laboratory temperature, during which the impedance measurement was also taken at different temperatures. Liquid nitrogen was used for cooling the water bath to 5°C. The impedance of the solution was measured at 10°C and 5°C. The precision of temperature control was maintained within ±1°C.

In addition, a reference test was conducted using the same heating and cooling configurations and the same solutions but without the impedance measurement. Meanwhile photographs were taken in order to visualize the dynamic precipitation process of calcium in the pore fluid during heating and cooling at important temperatures showing distinctive discrepancy in precipitations as presented in Figure 4.

1. Electrochemical impedance spectroscopy (EIS)

Development of impedance response of cementitious samples and the electrolytic solutions during thermal cycle were characterized via two-point EIS on an impedance spectrometer (PSM 3750 Newtons4th, Leicester, UK). The frequency range was configured to be 10 Hz – 10 MHz and the signal sweeping mode was 10 points per logarithmic cycle. The measuring voltage was configured at an RMS of 707.107 mV. A high-frequency fixture was employed to stabilize the signal perturbation at higher frequencies which guaranteed that the voltage and current responses both belonged to the material under test instead of the internal circuit of the impedance spectrometer. Zview software (Scribner, North Carolina, US) was used for data processing. The inner two electrodes were used for signal excitation and acquisition (Figure S7).


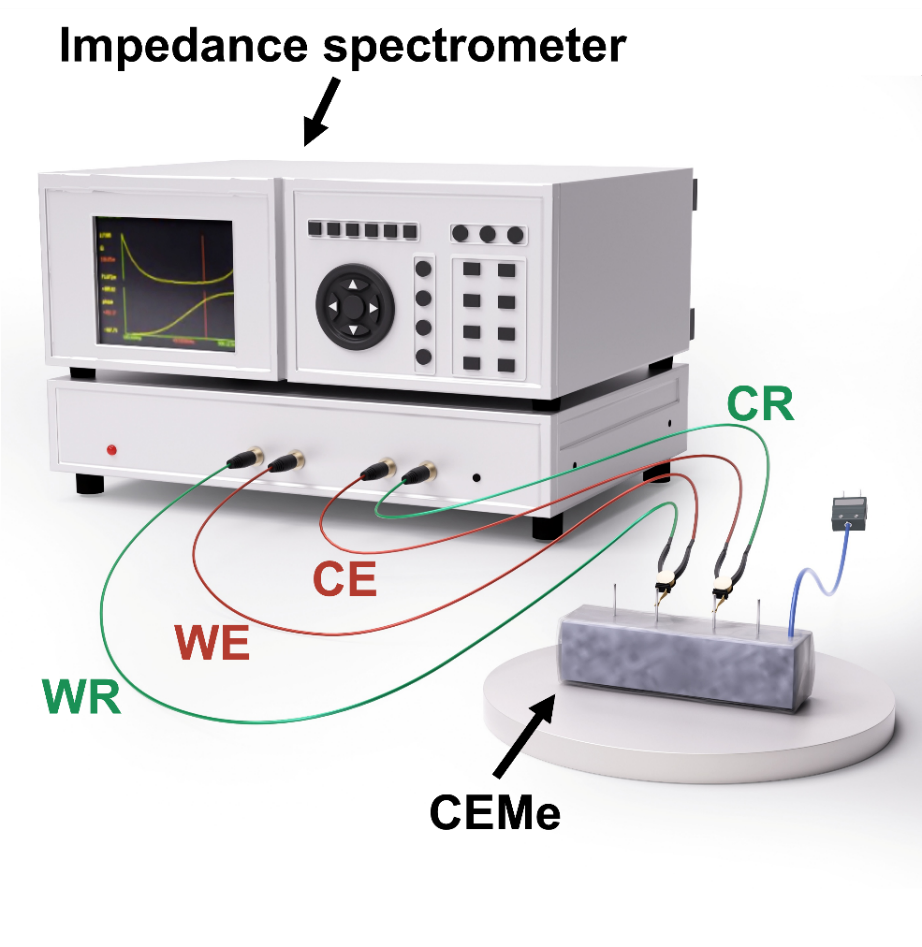


1. Two-point configuration of EIS measurement on CEMes during temperature intervals where CEMe samples were wrapped with PE film and connected with thermocouple. WR is working electrode reference, WE is working electrode, CE is the counter electrode, and CR refers to counter electrode reference.
2. Variation in moisture content during thermal cycle

Although the samples were well wrapped, moisture exchange between sample and the surrounding air was inevitable during thermal cycle due to water vapour travel through the voids and interstices of PE layers. The change in moisture content during heating and cooling must not be omitted because the ionic conduction through continuously connected liquid filled pore is one of the most important conduction pathways in the mortar matrix, in turn influencing the thermal dependence of impedance of the samples. Therefore, out of rigor of this study, the change in moisture content during thermal cycle has been recorded at each time whenever the impedance was measured. It can be seen in Figure S8a that most of the moisture loss (i.e., reduction in mass) took place during the heating stage, where the greatest moisture loss was on S8 (i.e., 4.35 g) and the lowest moisture loss was on PM (i.e., 2.64 g). During the cooling stage, all samples had marginal changes in moisture content. It can therefore be concluded that the moisture loss has been minimized through PE wrapping.


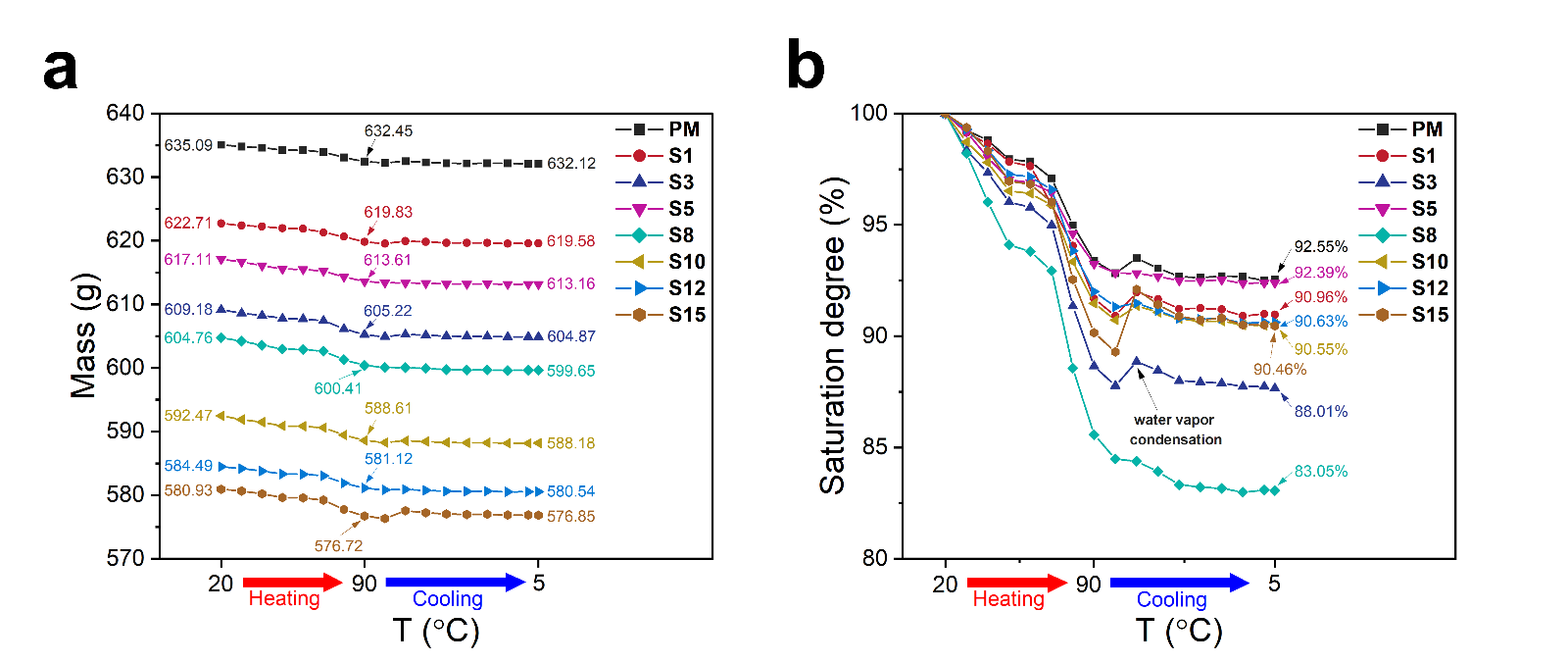


1. Monitoring on moisture content during heating and cooling stages, mass measurement interval was 10 °C. a) development of sample mass. b) development of saturation degree.

The dynamic states of moisture exchange between testing samples can be better realized by normalizing moisture content into the degree of saturation [13], as depicted in Figure S8b. Throughout the thermal cycle, the degree of saturation for all samples maintained at around 90% except for S8 settling at around 83% at the end of cooling. Based on previous study [11], it can be safe to conclude that continuity of ionic conduction pathway has been well preserved through the measure of PE wrapping and the moisture loss during thermal cycle has been significantly minimized. It can therefore be safe to define the moisture condition of the samples as “near water saturated” throughout the overall testing process. Noticeably, there exists an increase in saturation degree whilst cooling from 80°C to 70°C for all samples (excluding S8 and S5). This can be corresponded to condensation of vapour into liquid on sample surface.

1. Chemical equilibria of simulated pore solution

SI – 6.1. Determination programme

The pore solution of OPC is mainly comprised of Na^+^, K^+^, OH^-^, Ca^2+^, and SO_4_^2-^. These ionic species have fast ionic mobility and much higher concentration in comparison to other chemicals hence contributing predominantly to the ionic conduction of PM and CEMes [14] (Table S6). The chemical composition of simulated pore solution was constructed based on the principle that the pore solution was either saturated or supersaturated with respect to Ca(OH)_2_ from early hydration to long term hardening stages of OPC for a period as long as 4 months [15].

Molar concentrations of high activity coefficient ionic species, K^+^, Na^+^, and OH^-^, which were from high K_sp_ solutes (i.e., KOH and NaOH), was determined by NIST’s prediction model [4,5] using the XRF data on the percentage of alkaline oxides (Table S1), which were 0.09 mol/L, 0.45 mol/L, and 0.54 mol/L, respectively. NIST’s model was dedicated to estimate the concentration of the alkalis in pore solution at certain degree of hydration based on the assumption that 75% of the alkali oxides were dissolved upon mixing using the data of water/cement ratio, percentage of SCMs, as well as the alkali and silica contents. The model uses empirical coefficients of ionic species at infinite dilution for constructing the equivalent conductivity of a multicomponent aqueous solution and directly uses IS for adjusting the influence of interionic environment on the equivalent conductivity. The degree of hydration was estimated based on Transportation Research Board (TRB)’s database and Knudsen’s kinetic model [16–19], which was 78.78% at 97 days of curing in this study.

Molar concentrations of low activity coefficient ionic species, Ca^2+^ and SO_4_^2-^, which were from low K_sp_ solutes Ca(OH)_2_ and CaSO_4_, was determined through a combination of Moragues et al.’s method [7], Bromley’s model [20], and the principle of electroneutrality [21]. It has been proved that sharing the concentration of Ca^2+^ between slightly dissolved Ca(OH)_2_ and CaSO_4_ by simply applying a constant solubility product K_sp_ was unable to produce satisfactory results for the prediction of the concentrations of Ca^2+^ and SO_4_^2-^ [6,22,23]. The K_sp_ of Ca(OH)_2_ in highly concentrated aqueous solution increases with the increasing IS $I$ in a thermodynamic manner [7,24,25]. The IS $I$ is defined as

| $I=\frac{1}{2}\sum_{i=1}^{n} c_{i}z_{i}^{2}$ | (S1) |
| --- | --- |

where $c_{i}$ is the ionic concentration of $i$th ion in mol/L, $z_{i}$ the charge of $i$th ion.

From Equation S1, it can be approximated that pore solution in this study has an $I$ of

| $I=\frac{1}{2}\left( \left[ {Na}^{+} \right]+\left[ K^{+} \right]+\left[ {OH}^{-} \right]+4*\left[ {Ca}^{2+} \right]+4*\left[ {SO}_{4}^{2-} \right] \right)=0.54+2\left[ {Ca}^{2+} \right]+2\left[ {SO}_{4}^{2-} \right]$ | (S2) |
| --- | --- |

being > 0.54 mol/L, which was beyond the capabilities of Davies equation which was commonly used in thermodynamics of cementitious materials (i.e., of an IS lower than 0.5 mol/L) [25,26], as well as the classic Debye–Hückel equation which was based on diluted condition. In addition, it can be predicted that if assuming the concentrations of Ca^2+^ and SO_4_^2-^ are very low, the final IS should be above but close to 0.54 mol/L. The Bromley’s computation has been valid for an IS as high as 6 mol/L, which was suitable for simulated pore solution in this study with an IS exceeding 0.54 mol/L. First of all, the relationship between the concentration of Ca^2+^ and SO_4_^2-^ can be defined through electroneutrality:

| $\left[ {Na}^{+} \right]+\left[ K^{+} \right]+2*\left[ {Ca}^{2+} \right]=2*\left[ {SO}_{4}^{2-} \right]+\left[ {OH}^{-} \right]$ | (S3) |
| --- | --- |

using the data predicted via NIST’s model, Equation S3 yields

| $\left[ {Ca}^{2+} \right]=\left[ {SO}_{4}^{2-} \right]$ | (S4) |
| --- | --- |

Combining Eqs. S2 and S4, the $I$ of simulated pore solution can be rewritten into a function of $\left[ {Ca}^{2+} \right]$:

| $I=\frac{1}{2}\left( \left[ {Na}^{+} \right]+\left[ K^{+} \right]+\left[ {OH}^{-} \right]+4*\left[ {Ca}^{2+} \right]+4*\left[ {SO}_{4}^{2-} \right] \right)=0.54+4*\left[ {Ca}^{2+} \right]$ | (S5) |
| --- | --- |

Through Bromley’s estimation (a modified Debye–Hückel model), the $I$ influences the mean activity coefficient of Ca(OH)_2_, $\gamma_{CH}$, via a temperature dependent manner, that:

| $\log\gamma_{CH}=\frac{-M\left\vert z_{C}z_{H} \right\vert I^{\frac{1}{2}}}{1+\rho I^{\frac{1}{2}}}+\frac{(0.06+0.6Q)\left\vert z_{C}z_{H} \right\vert I}{{(1+gI)}^{2}}+QI$ | (S6) |
| --- | --- |

where $M$ and $Q$ are temperature dependent coefficients and equals to 0.51 and 0.0056 respectively in the case of Ca(OH)_2_ at laboratory temperature [27,28], $z_{C}$ and $z_{H}$ the charges of Ca^2+^ and OH^-^ respectively, $\rho$ and $g$ are also dependent on the temperature which are 0.8 and 0.75 in the case of simulated pore solution in our study under laboratory temperature [20].

Moragues et al. [7] suggested that the K_sp_ of Ca(OH)_2_ was a power function of the total $I$ under the presence of SO_4_^2-^, that:

| $K_{sp}=-5.8\times{10}^{-4}+8.05\times{10}^{-4}+I^{0.14461}$ | (S7) |
| --- | --- |

and the apparent (or thermodynamic) $K_{sp}$ formulation [24,27,29] is

| $\left[ {Ca}^{2+} \right]\left[ {OH}^{-} \right]^{2}=\frac{K_{sp}}{{\gamma_{CH}}^{3}}$ | (S8) |
| --- | --- |

Combining Eqs S5, S6, S7, and S8, the $I$ of simulated pore solution in our study was eventually determined to be 0.59 mol/L and both the concentrations of Ca^2+^ and SO_4_^2-^ are 0.012 mol/L. The molar concentrations of ionic species of PM and CEMes at 97 days of curing age are presented in Table S4, according to which the simulated pore solutions were prepared in Section SI – 2.2, Supporting Information.

SI – 6.2. Validation programme

In Taylor’s work [9], the concentrations of sulphates and alkalis in the pore solution of OPC system based on the measured data collected from different sources had the following relationship:

| $\left[ {SO}_{4}^{2-} \right]=0.06*\left( \left[ K^{+} \right]+\left[ {Na}^{+} \right] \right)^{2}$ | (S9) |
| --- | --- |

The results calculated by Equation S9 was 0.017 mol/L for SO_4_^2-^, which was close to but slightly higher than the molarity of SO_4_^2-^ 0.012 mol/L in this study.

1. Thermodynamic modelling

SI – 7.1. Methodology

A thermodynamic modelling programme was used to predict the stable phases at different equilibrium temperature. The open-source software GEM-Selektor v.3 (<http://gems.web.psi.ch/GEMS3/>) was used to perform the modelling. The extended Debye–Hückel equation was used for calculating the activity coefficients of the aqueous species. The most recent Cemdata18 (<https://www.empa.ch/web/s308/thermodynamic-data>) was used as the main chemical thermodynamic database. The solid solution models CSHQ was used for calculating the calcium silicate hydrate (C-S-H) phase. The chemical compositions of the end members are described in detail in Lothenbach et al.’s work [30]. CEM**Ⅰ**was added to the model based on the XRF results shown in Table S1, and the degree of reaction (DoR) was configured at 78.78%. It was assumed that the LOI of the CEM**Ⅰ**clinker used in this study was contributed by the presence of limestone (CaCO_3_). Therefore, equivalent amount of CaCO_3_ was added to the model for replicating the cement system produced in this study. A water to cement clinker ratio of 0.45 was used, in consistency with the experimental design. The simulation of hydration products was first performed and validated at 20 °C and under standard pressure. Then, a prediction process was created based on the validated benchmark, changing the equilibrium temperature from 25 °C to 90 °C with a step size of 5 °C. The DoR and initial input were kept constant during the process. The mass of the solid reaction products was calculated, as well as the pH, total ionic strength, and ionic speciation concentrations in the pore solutions. The results were summarised in the main context and in the following sections.

SI – 7.2. Modelling results for low-concentration species in pore solution


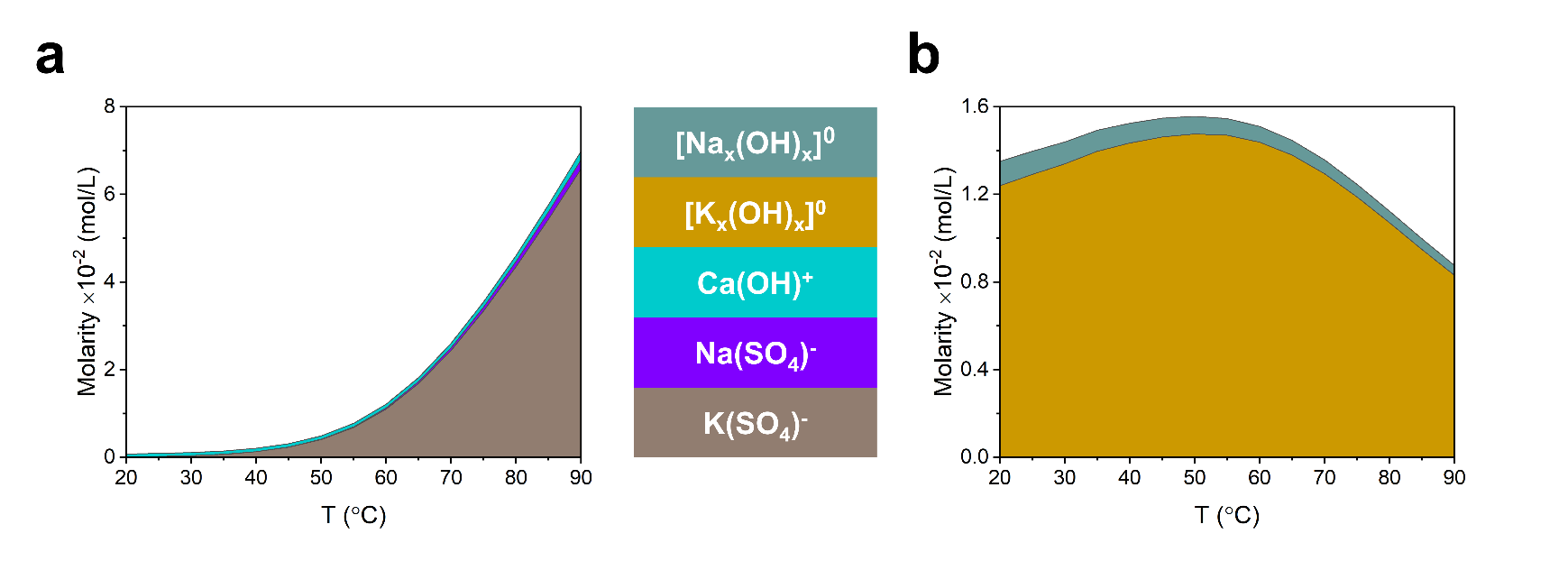


1. Molar concentrations of low-concentration species in pore solution through GEMs modelling considering the ion speciation effect. a) charged large and heavy ionic species. b) neutral ion clusters.

SI – 7.3. Modelling results for variation of hydration products in the form of both volume and mass


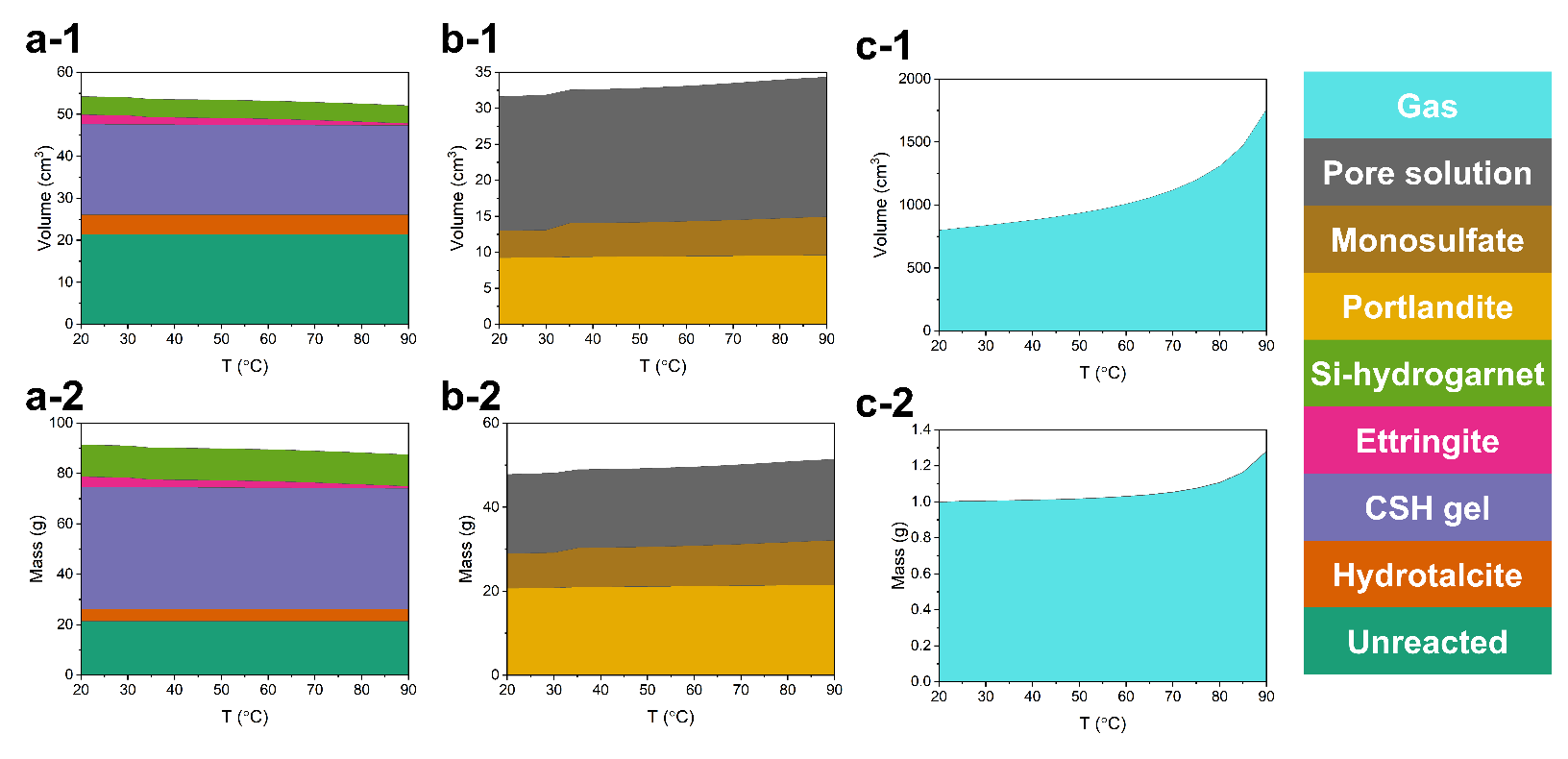


1. Variation in hydration products when heating from 20 to 90°C. a-1 and 2) hydration products whose volume and mass decreased (CSH gel, ettringite, Si-hydrogarnet) and unchanged (unreacted cement, hydrotalcite). b-1 and 2) hydration products whose volume and mass increased (portlandite, monosulfate, pore solution). c-1 and 2) volume and mass of gas phase (The mass of gypsum at 97 days of curing was zero due to being totally consumed through hydration. Raw GEMs simulation output was archived in University of Bath Research Data Archive DOI: <https://doi.org/10.15125/BATH-01577>.).

SI – 7.4. Modelling results for thermodynamic activity coefficient and correction factor

1. Thermodynamic activity coefficient $\gamma_{i}(T)$ and correction factor $\alpha_{i}(T)$

| **T (°C)** | $\boldsymbol{\gamma}_{\boldsymbol{i}}\boldsymbol{(}\boldsymbol{T}\boldsymbol{)}$ | | | | | $\boldsymbol{\alpha}_{\boldsymbol{i}}\boldsymbol{(}\boldsymbol{T}\boldsymbol{)}$ | | | | |
| --- | --- | --- | --- | --- | --- | --- | --- | --- | --- | --- |
|  | **Ca^+2^** | **K^+^** | **Na^+^** | **SO_4_^-2^** | **OH^-^** | **Ca^+2^** | **K^+^** | **Na^+^** | **SO_4_^-2^** | **OH^-^** |
| 20 | 0.239 | 0.742 | 0.742 | 0.239 | 0.742 | 0.424 | 0.6 | 0.6 | 0.424 | 0.6 |
| 25 | 0.236 | 0.740 | 0.740 | 0.236 | 0.740 | 0.424 | 0.6 | 0.6 | 0.424 | 0.6 |
| 30 | 0.234 | 0.738 | 0.738 | 0.234 | 0.738 | 0.424 | 0.6 | 0.6 | 0.424 | 0.6 |
| 35 | 0.231 | 0.736 | 0.736 | 0.231 | 0.736 | 0.424 | 0.6 | 0.6 | 0.424 | 0.6 |
| 40 | 0.228 | 0.733 | 0.733 | 0.228 | 0.733 | 0.424 | 0.6 | 0.6 | 0.424 | 0.6 |
| 45 | 0.224 | 0.730 | 0.730 | 0.224 | 0.730 | 0.424 | 0.6 | 0.6 | 0.424 | 0.6 |
| 50 | 0.220 | 0.727 | 0.727 | 0.220 | 0.727 | 0.424 | 0.6 | 0.6 | 0.424 | 0.6 |
| 55 | 0.214 | 0.723 | 0.723 | 0.214 | 0.723 | 0.424 | 0.6 | 0.6 | 0.424 | 0.6 |
| 60 | 0.207 | 0.719 | 0.719 | 0.207 | 0.719 | 0.424 | 0.6 | 0.6 | 0.424 | 0.6 |
| 65 | 0.199 | 0.714 | 0.714 | 0.199 | 0.714 | 0.424 | 0.6 | 0.6 | 0.424 | 0.6 |
| 70 | 0.190 | 0.708 | 0.708 | 0.190 | 0.708 | 0.424 | 0.6 | 0.6 | 0.424 | 0.6 |
| 75 | 0.181 | 0.702 | 0.702 | 0.181 | 0.702 | 0.424 | 0.611 | 0.611 | 0.424 | 0.611 |
| 80 | 0.172 | 0.697 | 0.697 | 0.172 | 0.697 | 0.424 | 0.635 | 0.635 | 0.424 | 0.635 |
| 85 | 0.163 | 0.691 | 0.691 | 0.163 | 0.691 | 0.424 | 0.659 | 0.659 | 0.424 | 0.659 |
| 90 | 0.155 | 0.685 | 0.685 | 0.155 | 0.685 | 0.424 | 0.683 | 0.683 | 0.424 | 0.683 |

1. Calculation of electrical parameters

SI – 8.1. Intrinsic bulk conductivity, relative permittivity, and loss tangent (using as-measured experimental data)

The measured impedance of a composite (i.e., PM and CEMe) or a multicomponent (i.e., simulated pore solution) system is a sum of the total conduction and polarization processes within the material under AC electric field excitation, which is expressed through a complex format:

| $Z\left( \omega\right)=Z'\left( \omega\right)-jZ''\left( \omega\right)$ | (S10) |
| --- | --- |

where $j=\sqrt{-1}$, $Z'\left( \omega\right)$ the real component (i.e., resistance $R$), $Z''\left( \omega\right)$ the imaginary component (i.e., capacitive reactance $X$), and $\omega$ the angular frequency (unit in rad/s). The frequency-dependent conductivity $\sigma\left( \omega\right)$ and relative permittivity $\varepsilon_{r}(\omega)$ is de-embeded from the measured data via the following expression:

| $\sigma\left( \omega\right)=\frac{Z'\left( \omega\right)}{\left[ Z'\left( \omega\right) \right]^{2}+ \left[ Z"(\omega) \right]^{2}}\cdot\frac{1}{k}$ | (S11) |
| --- | --- |

| $\varepsilon_{r}(\omega)=\frac{Z"\left( \omega\right)}{\varepsilon_{0}\omega\left\{ \left[ Z'\left( \omega\right) \right]^{2}+ \left[ Z"\left( \omega\right) \right]^{2} \right\}}\cdot\frac{1}{k}$ | (S12) |
| --- | --- |

where $\varepsilon_{0}$ is the absolute permittivity of a vacuum (8.854×10^-12^ F/m), $\omega$ the angular frequency, $k$ the geometric factor of the material under test, which was calculated to be 0.0259 m for PM and CEMes, as well as 0.02 m through the method in Ball et al. [31]. Intrinsic bulk conductivity of PM and CEMes are obtained at the cusp frequency points where the surface mechanism diminishes and bulk mechanism initiates, as identified for bulk resistance R_b_ in Figure 1. Intrinsic bulk conductivity of PM and CEMes are all denoted as $\sigma$ with contextual descriptions unless described explicitly. Intrinsic bulk conductivity of simulated pore solution, potassium hydroxide solution, and saturated calcium hydroxide solution are denoted as $\sigma_{SPS}$, $\sigma_{KOH}$, $\sigma_{SCH}$, respectively.

The loss tangent tanδ, which quantifies the ability of a material to transfer electrical energy into heat loss, is thus calculated via the ratio of imaginary permittivity over the real permittivity, that:

| $tan\delta(\omega)=\frac{\varepsilon"(\omega)}{\varepsilon'(\omega)}=\frac{\sigma\left( \omega\right)}{{\omega\varepsilon}_{r}(\omega)}$ | (S13) |
| --- | --- |

where the peak tanδ is obtained at the frequency where the tanδ reaches its highest value at Bode format, which corresponds to dielectric relaxation and energy loss at certain polarization domain, as described in Figure 1.

SI – 8.2. Intrinsic pore solution conductivity (using thermodynamic simulation data)

The intrinsic conductivity of pore solution at different temperatures determined by the thermodynamic simulations was approximated via the modified Nernst-Einstein relation:

| $\sigma_{PS}=\frac{F^{2}}{RT}\sum_{i}^{n} {z_{i}}^{2}D_{i}(T)c_{i}(T)\left[ \gamma_{i}(T) \right]^{\alpha_{i}(T)}$ | (S14) |
| --- | --- |

where $c_{i}(T)$ is the temperature-dependent molar concentrations of the five major free ions, Na^+^, K^+^, OH^-^, Ca^2+^, and SO_4_^2-^, from thermodynamic data in Figure 3c-1 and c-2 (unit in mol/m^3^), $D_{i}(T)$ the temperature-dependent diffusion coefficient of $i$th ion (unit in m^2^/s), $\gamma_{i}(T)$ the temperature-dependent activity coefficient of $i$th ion which was estimated via extended Debye–Hückel equation [32] (Section SI – 7.4, Supporting Information), $\alpha_{i}(T)$ the correction factor (Section SI – 7.4, Supporting Information), R the ideal gas constant 8.31446 J/(K·mol), $F$ the Faraday’s constant 96485 J/mol.

The temperature-dependent diffusion coefficient is calculated using Stokes-Einstein-Sutherland equation:

| $D_{i}\left( T \right)=\frac{k_{B}T}{6\pi\eta(T)r_{i}}$ | (S15) |
| --- | --- |

where $k_{B}$ the Boltzmann constant 8.617 × 10^-5^ eV/K, $\eta(T)$ the dynamic viscosity of water in g/(m·s) which was obtained from International Association for the Properties of Water and Steam (IAPWS R12-08) [33,34], $r_{i}$ the stokes radius of $i$th ion (unit in m).

The correction factor $\alpha_{i}(T)$ is dependent on temperature and IS via:

| $\alpha_{i}\left( T \right)=\left\{ \begin{aligned} \frac{0.6}{\sqrt{\left\vert z_{i} \right\vert}}, &I\leq0.36\left\vert z_{i} \right\vert\\ \frac{\sqrt{I}}{\left\vert z_{i} \right\vert}, &I>0.36\left\vert z_{i} \right\vert\end{aligned} \right.$ | (S16) |
| --- | --- |

It can be seen that the modified Nernst-Einstein relation has its basic form of Nernst–Einstein equation. The activity coefficient, correction factor, and dynamic viscosity were used to scale the hinderance/facilitation effect of ion-ion interaction on the migration of free ions [35,36].

1. Calculation of activation energies

SI – 9.1. Arrhenius behaviour

Classic Arrhenius approach was used to obtain the Activation energy of a materials system exhibiting Arrhenius behaviour:

| $\sigma=\sigma_{0}e^{-\frac{E_{a}}{k_{B}T}}$ | (S17) |
| --- | --- |

where $\sigma$ the intrinsic electrical conductivity in S/m, $E_{a}$ the activation energy required for the electrical conduction process (i.e., long-range drift of charge carriers in the direction of electric field) in eV per ion, $T$ the temperature in K, $k_{B}$ the Boltzmann constant 8.617 × 10^-5^ in eV/K, and $\sigma_{0}$ is the pre-exponential factor in S/m.

Equation S17 has a linearised form by logarithmic transform:

| $\ln\sigma=\ln\sigma_{0}-\frac{E_{a}}{k_{B}}\cdot\frac{1}{T}$ | (S18) |
| --- | --- |

The activation energy $E_{a}$ in this study was thus extracted through the slope of fitted line (i.e., $\ln\sigma$ against $\frac{1}{T}$).

SI – 9.2. Non-Arrhenius behaviour

For a materials system exhibiting non-Arrhenius behaviour, an innovative phenomenological approach was used, for the first time, under the principle where $-\frac{E_{a}}{k_{B}}$ is the algebraic tangent of the correlation between $\ln\sigma$ and $\frac{1}{T}$. Therefore, activation energy of non-Arrhenius behaviour can be obtained by calculating the algebraic tangent of the non-linear curve if a phenomenological fitting was successful.

For S15 during heating with non-Arrhenius behaviour, an exponential law was fitted through between $\ln\sigma$ and $\frac{1}{T}$ with an R^2^ of 0.99 (Figure 2d):

| $\ln\sigma=d_{1}+d_{2}e^{\frac{d_{3}}{T}}$ | (S19) |
| --- | --- |

where $d_{1}$, $d_{2}$, and $d_{3}$ are the coefficients with a value of -2, 3.8 × 10^6^, -5 × 10^3^, respectively.

The derivative of Equation S19 is

| $\frac{d(\ln\sigma)}{d(\frac{1}{T})}=d_{2}d_{3}e^{\frac{d_{3}}{T}}$ | (S20) |
| --- | --- |

Analogous to the classic Arrhenius form (Equation S18), one can write

| $-\frac{E_{a}}{k_{B}}=d_{2}d_{3}e^{\frac{d_{3}}{T}}$ | (S21) |
| --- | --- |

The changing activation energy of S15 during heating can thus be quantified through

| $E_{a}=-k_{B}d_{2}d_{3}e^{\frac{d_{3}}{T}}$ | (S22) |
| --- | --- |

For pore solution (Figure 3d-1) and conduction contribution of OH^-^ ion (Figure 3d-2) under heating exhibiting non-Arrhenius behaviour, polynomial fitting was successful:

| $\ln\sigma_{PS}or \ln\frac{\sigma_{{OH}^{-}}(T)}{\sigma_{{OH}^{-}}(20^{\circ}C)}=A_{poly}+\sum_{i}^{n} B_{i}\frac{1}{T^{i}}$ | (S23) |
| --- | --- |

where $n$ equals to 5 indicating a 5-order polynomial fitting for both the fluctuational behaviour of pore solution and OH^-^ ion, $A_{poly}$ and $B_{i}$ are coefficients and the results are recorded in Table S8.

1. Polynomial fitting results for non-Arrhenius behaviour of pore solution (Figure 3d-1) and conduction contribution of OH^-^ ion (Figure 3d-2) under heating from thermodynamic modelling data

| **Fitting coefficients** | **Pore solution** | **Conduction contribution of OH^-^ ion** |
| --- | --- | --- |
| A_poly_ | 10532.13441 | 7047.79619 |
| B_1_ | -1.70897 × 10^7^ | -1.16878 × 10^7^ |
| B_2_ | 1.10565 × 10^10^ | 7.70328 × 10^9^ |
| B_3_ | -3.56505 × 10^12^ | -2.52419 × 10^12^ |
| B_4_ | 5.7308 × 10^14^ | 4.11524 × 10^14^ |
| B_5_ | -3.67541 × 10^16^ | -2.67239 × 10^16^ |

1. Meyer–Neldel Rule (MNR) validation

An important feature for thermally activated process of a materials system exhibiting Arrhenian behaviour is the compliance of Meyer Neldel Rule (MNR). Since the first discovery in 1937, the MNR was found to exist in many physio-chemical processes (i.e., electrical conduction, dielectric relaxation, ionic diffusion, etc.) in materials such as crystalline and amorphous semiconductors [37], ionic conductors [38], silicate glass [39], composites [40], etc. Referring back to Equation S17, the MNR, exponentially correlates the Arrhenius pre-exponential factor $\sigma_{0}$ to the activation energy $E_{a}$:

| $\sigma_{0}=\sigma_{00}e^{\frac{E_{a}}{E_{MN}}}$ | (S24) |
| --- | --- |

or a more commonly applied linear form:

| $\ln\sigma_{0}=\ln\sigma_{00}+\frac{1}{E_{MN}}\cdot E_{a}$ | (S25) |
| --- | --- |

where $\ln\sigma_{00}$ is the intercept, $E_{MN}$ the slope and termed as Meyer Neldel energy in eV per ion.

Combining Equation S25 with Equation S18, a linear correlation between natural logarithm of conductivity $\ln\sigma$ and activation energy $E_{a}$ can be obtained:

| $\ln\sigma=\ln\sigma_{00}+E_{a}\left( \frac{1}{E_{MN}}-\frac{1}{k_{B}}\cdot\frac{1}{T} \right)$ | (S26) |
| --- | --- |

From Equation S26, it can be seen that there exists a temperature at which the bulk conductivity is independent of activation energy and equals to $\sigma_{00}$ under a characteristic $E_{MN}$, that:

| $\frac{1}{E_{MN}}-\frac{1}{k_{B}}\cdot\frac{1}{T}=0$ | (S27) |
| --- | --- |

yielding

| $T_{iso}=\frac{E_{MN}}{k_{B}}$ | (S28) |
| --- | --- |

where $T_{iso}$ is often termed as isokinetic temperature in K [41].

1. Microstructural characterization

SI – 11.1. Backscattered electrons imaging (SEM – BSE) and Energy Dispersive X-Ray Spectroscopy (EDX)

SI – 11.1.1. Methods

BSE imaging was used to examine the morphology of fracture surface and how the solid components in CEMe (i.e., carbon fibre, hydration products, unreacted cement, sand) interact with each other. EDX element analysis was used to determine the elemental distribution on the surface of carbon fibres upon hardening of CEMe. A SU-3900 scanning electron microscope (SEM) machine (HITACHI, Tokyo, Japan) equipped with a 170 mm^2^ UltiMax detector (Oxford Instruments, Abingdon, UK) was used. The accelerating voltage and beam current were configured at 20 kV and 86 μA, respectively. Prior to microscopic examination, sample from the same mixing batch (Section SI – 2.1, Supporting Information) and curing protocol was fractured in half at the centre point between the two inner electrodes (i.e., the location where the impedance was measured), which were then taken for the same drying process until mass equilibrium (Section SI – 3.1.2, Supporting Information). Samples for examination are at pristine condition without thermal cycling.

SI – 11.1.2. Results

In Figure S11a at 200 times of magnification, the carbon fibres are dispersed in cement matrix in a random manner. CSH and unreacted cement are also visually noticeable. The geometrical arrangement of ionic and electronic conduction pathways is evident as depicted in In Figure S11b at 800 times of magnification. The pores, fibre-matrix interfaces, and microcracks coexists with each other, which can all be pore solution reservoirs in turn conducting electricity ionically. The carbon fibres coexist with the pore solution reservoirs to conduct electricity electronically. Such coexistence contributes to a “dual arc” complex impedance feature for CEMe were both surface and bulk responses are pronounced (Figure 1b-1). In comparison to the carbon fibres, it is evident that the pore solution reservoirs took up large volume of the matrix, hence the ionic conduction cannot be ignored when studying conduction mechanism of CEMe. This reversely justifies the legitimacy of testing “near water saturated” sample to realize the ionic conduction as much as possible. In In Figure S11c at 500 times of magnification, the portlandite flakes are detected which exist in both the matrix and fibre-matrix interface. In In Figure S11d at 2000 times of magnification, the amorphous CSH gel was noticeable appearing like a cloud. The ettringite needles are also detected and are found to exit in both the matrix and fibre-matrix interface. This justified the thermodynamic modelling that the decomposition of ettringite can create additional hollow gaps in fibre-matrix interface and voids in cement matrix to contribute to the “loosening” of pore network for easier ionic migration, hence a reduced $E_{a,ionic}$.

In In In Figure S11e, oxygen, calcium, silicon are high weight percentage elements to be detected together with carbon on the surface of carbon fibres. The aluminium, potassium, ferrite, magnesium, and sulphate elements are also detected but with marginal weight percentages. This suggests that the CSH gel is the main product to be present on the carbon fibre surface, whereas additional cementitious constituents are also present but with trivial amounts. This is because the carbon fibre surfaces, after being treated to be hydrophilic by PCE, can preserve water molecules, hence being additional nucleation site for the growth of hydration products and precipitation of leached products.


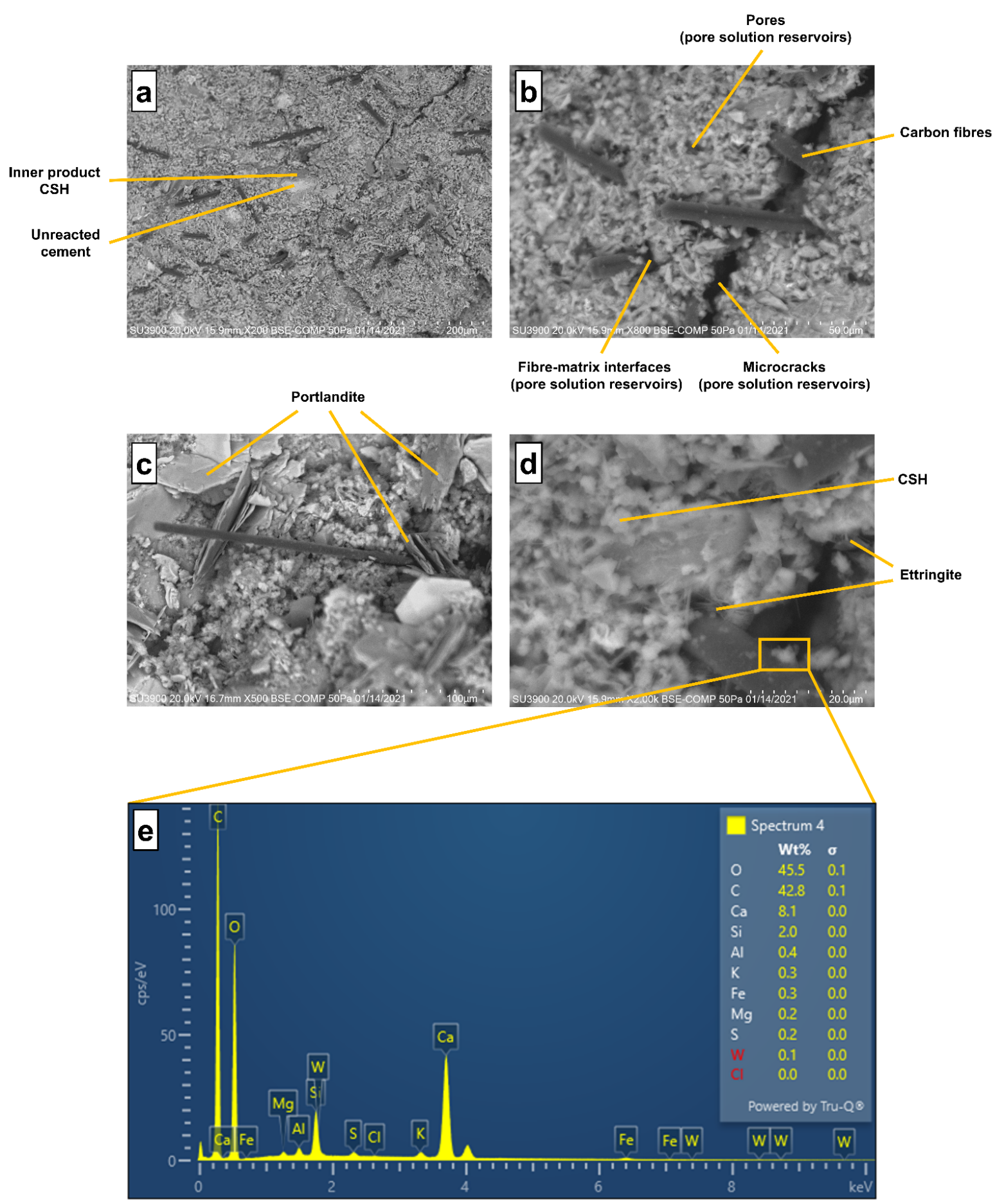


1. Morphological examination on the solid components in CEMe (i.e., S5 at the age of 97 days as an example) using backscattered electrons (SEM – BSE) imaging. a) fracture surface at the centre point of two inner measuring electrodes (× 200 magnification). b) enlarged examination on the geometrical arrangement of conduction pathways (× 800 magnification). c) detection of portlandite (× 500 magnification). d) detection of ettringite (× 500 magnification). e) EDX element analysis on the surface of carbon fibre.

SI – 11.2. Mercury Intrusion porosimetry (MIP)

SI – 11.2.1. Methods

Temperature-induced development in pore structure of PM and CEMes were characterized by mercury intrusion porosimetry (MIP). Pascal 140 and Pascal 440 porosimeters (Thermo Fisher Scientific Inc, Milan, Italy) were used for low- and high-pressure intrusion, respectively. Both intrusion and extrusion of mercury were performed within a pressure range of 0 – 400 MPa. The mercury contact angle was fixed at 140° throughout the pore range of 3 – 10^5^ nm. Porosity, pore size distribution, pore tortuosity, and pore surface fractality were quantified at pristine and residual conditions. Each testing sample has two reference samples from the same mixing batch for the MIP test (Figure S2e). One reference sample was crushed for MIP test immediately after finishing curing for the evaluation of the pore structure at pristine condition without any thermal treatment. Another reference sample, which was placed alongside with the testing sample in the climate chamber under the same configurations for thermal cycle, was used for the evaluation of microstructure after completion of thermal cycle. After the completion of thermal cycle, the reference sample was sealed in an air-tight zip-lock bag and placed under laboratory condition for 3 days until temperature equilibrium has been reached, after which being crushed for MIP test. To ensure a reliable correlation between pore structure and electrical properties, fragmental pieces were taken at the location between the two inner measuring electrodes where the impedance was measured (Figure S7). The fragments were dried to mass equilibrium as defined above under vacuum for MIP test.

SI – 11.2.2. Calculation of Tortuosity

Pore tortuosity $\tau$ was calculated using intrusion and extrusion data via Salmas and Androutsopoulos’s method [42]:

| $\tau=4.6242\ln(\frac{4.996}{1-\alpha_{en}}-1)-5.8032$ | (S29) |
| --- | --- |

where $\alpha_{en}$ is pore entrapment fraction which equals to the ratio of entrapped volume (difference between total intruded volume and total extruded volume) over the total intruded volume.

SI – 11.2.3. Calculation of Pore fractal dimension

It was found that the surface of the pore walls in cementitious materials possess scale-dependent fractal nature [43–45]. Pore fractal dimension D_s_ is a quantification of the morphological complexity and roughness of the surface of the pore walls. In conjunction with Nernst-Einstein relation, higher pore surface fractal dimension is accompanied by lower ionic diffusivity and electrical conductivity [46–50]. In the case of cement-based sensor with a mixed conduction of ions and electrons, higher pore fractal dimension indicates more frequent occurrence of solid obstacles for charge carriers to encounter, hence the increased difficulty for charge carriers to transport. In nature, fractal dimension has non-integer forms which describes geometries exhibiting irregularity (i.e., snowflakes, mountains, coastlines) [51]. Important features of fractal dimension were self-similarity and scale invariance, that, a surface (or curve) scales in a way where any part of itself would resemble the whole:

| $D_{s}=\frac{\log N_{s}}{\log R_{s}}$ | (S30) |
| --- | --- |

where $D_{s}$ is the fractal dimension, $N_{s}$ the number of copies of the original geometry, $R_{s}$ the iteration of scaling. For a surface with fractal nature, $D_{s}$ ranges from 2 to 3. A perfectly smooth surface has a fractal dimension of 2 and increase in $D_{s}$ means the increase in roughness and irregularity of the surface. If $D_{s}$ is approaching 3, the surface is complex enough to resemble a fully solid volume.

Based on the above universal theoretical background, the pore surface fractal dimension of PM and CEMes were calculated through Zhang and Li’s model [52]. Zhang and Li applied thermodynamics on the process of mercury intruding into the pores, and connected the surface area $S$ encountered during MIP to the total volume $V$ through an explicit fractal scaling law by Mandelbrot [51], that:

| $S^{\frac{1}{D_{s}}}\propto V^{\frac{1}{3}}$ | (S31) |
| --- | --- |

and a final quantification on pore surface fractal dimension $D_{s}$ based on MIP was established:

| $\ln\frac{W_{n}}{r_{n}^{2}}=D_{s}\ln\frac{{V_{n}}^{\frac{1}{3}}}{r_{n}}+C_{s}$ | (S32) |
| --- | --- |

where $r_{n}$ is the pore radius at $n_{th}$ step (unit in m), $V_{n}$ the intruded volume at $n_{th}$ step (unit in m^3^), $C_{s}$ a constant, and $W_{n}$ is the cumulative work down by the mercury during intrusion at $n_{th}$ step, which was calculated through:

| $W_{n}=\sum_{i=1}^{n} P_{i}\Delta V_{i}$ | (S33) |
| --- | --- |

where $P_{i}$ (unit in N/m^2^) and $\Delta V_{i}$ is the applied pressure and increased intrusion volume at $i_{th}$ step respectively, and $1\leq i\leq n$.

SI – 11.2.4. Visualization of D_s_1 and D_s_2 in CEMe by characteristic fractal geometries

In Figure 5f and g, at both pristine and residual states, the D_s_1 had values between around 2.6 and 2.9, whilst D_s_2 ranged from 2.3 to 2.6. In Figure S12, various ideal fractal geometries with surface fractal dimensions from a perfectly smooth surface (i.e., D_s_ = 2) to a solid 3D volume without any holes (i.e., D_s_ = 3), were presented, which can be taken as references to visualize the morphological complexity of pore surfaces quantified by D_s_1 and D_s_2. The horizontal change from right side to the left can be an idea visualization of the smoothing process of pore walls during thermal cycle as presented in Figure 5.


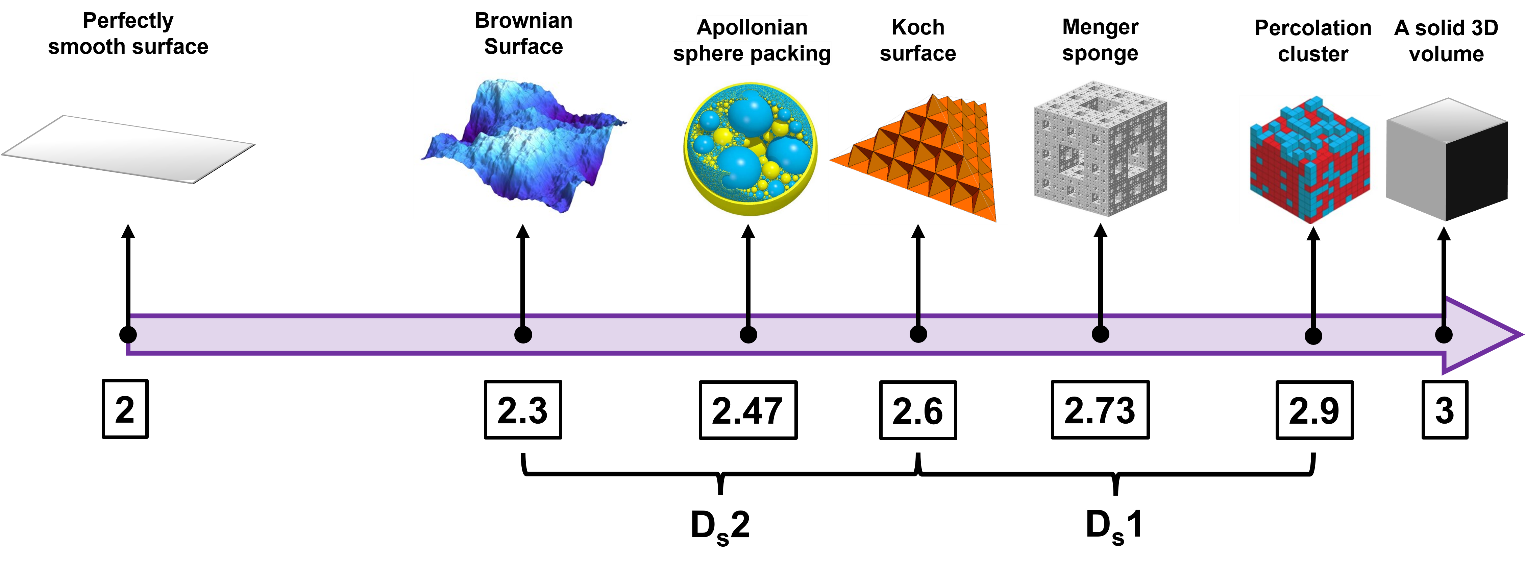


1. Visualization of D_s_1 and D_s_2 by characteristic fractal geometries with fractal dimensions between 2 and 3 [53–63].

The lower and upper boundaries of D_s_2, 2.3 and 2.6, can be visualized by Brownian surface and Koch surface, respectively. In particular, the Koch surface is constructed via building tetrahedrons at the midpoint of the original 2D equilateral triangle, which resembles the repeating surface morphology of silicious tetrahedra layers in C-S-H gel [64]. As to a more complex morphology for D_s_1, 2.6 – 2.9, the Menger sponge scales continuously by dividing the original solid cube into 27 identical cubes and remove the centre cube of each face, where the morphological development of the empty space resembles the percolating process of capillary pore network [65,66] which is exactly within the pore size range of regionⅠ.Comparably, the scaling of percolation cluster obeys percolation law, which can reach a surface fractal dimension as high as 2.9 indicating an ultimately dense geometry nearly filling the whole empty space. This is an indicative of an extremely difficult condition for ionic species to continuously travel through like the D_s_1 = 2.9 for S12 at pristine condition in Figure 5f.

SI – 11.2.5. Differential intrusion results


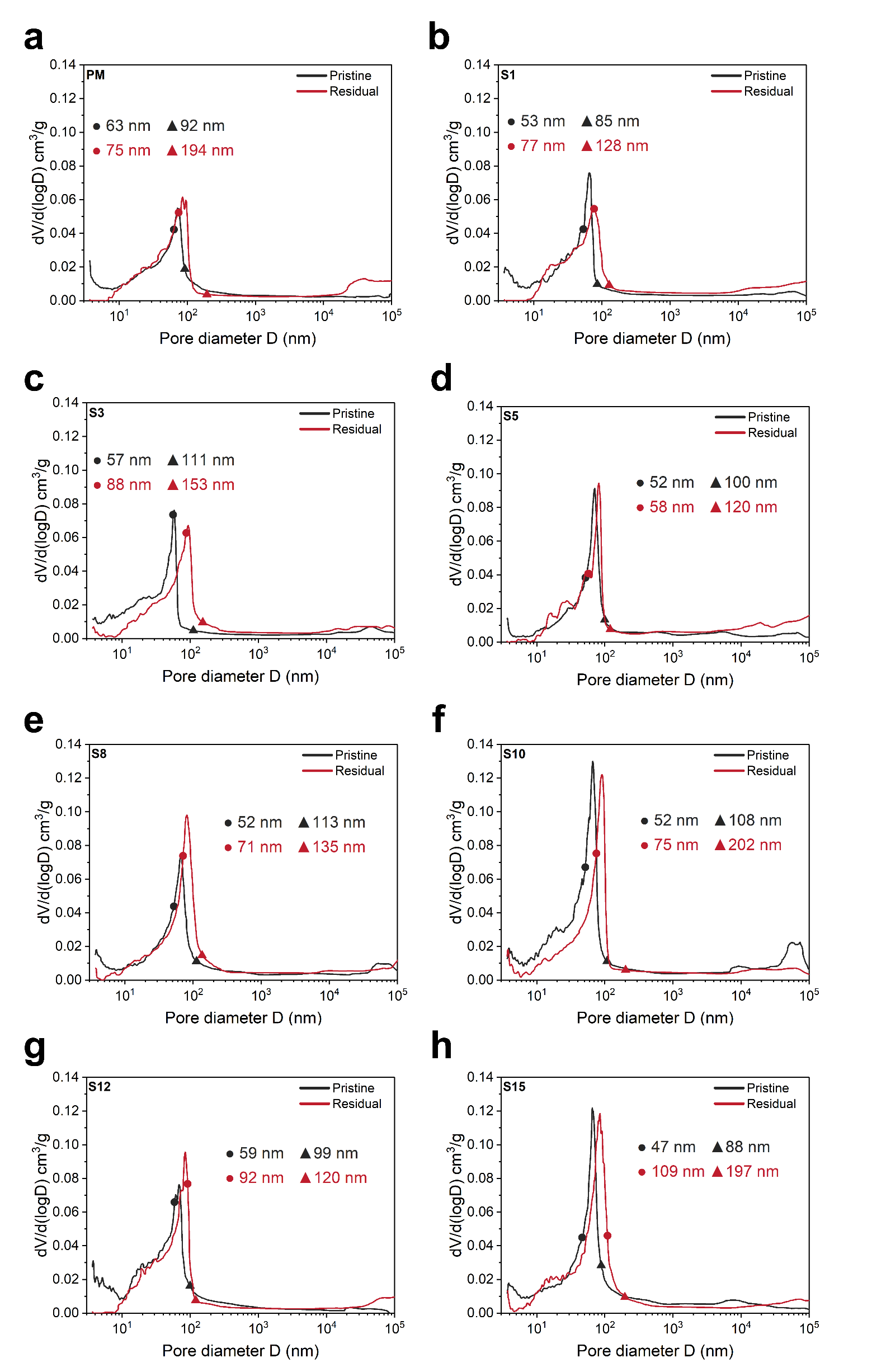


1. Comparison of differential pore intrusion results between pristine and residual states for PM and CEMes: to be consistent with Figure 5e, the upper pore size boundary of regionⅡ is marked as circle, the lower boundary of regionⅠis marked as triangle, and the region of transition resides between the circle and triangle. a) PM. b) S1. c) S3. d) S5. e) S8. f) S10. g) S12. h) S15.
2. Development of electrical conductivity through curing age

SI – 12.1. Complex impedance of PM and CEMes at 97 days of curing within a fabrication batch


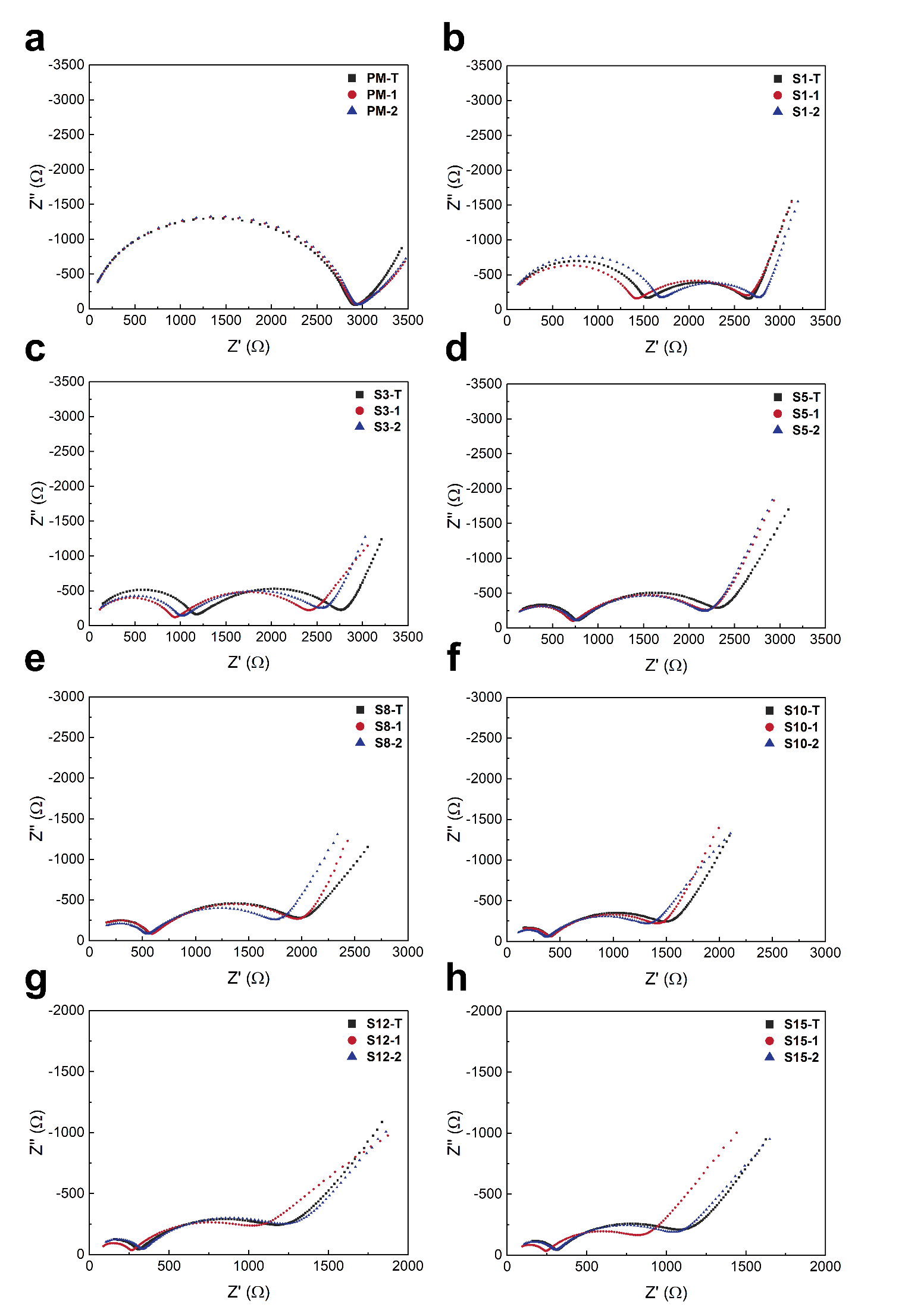


1. Impedance responses of the three samples from one fabrication batch for each fibre content at 97 days of curing at their pristine condition. a) PM. b) S1. c) S3. d) S5. e) S8. f) S10. g) S12. h) S15 (Thermal cycle testing samples are referred to as “sample name - T” and samples for microstructural characterizations are referred to as “sample name – number”. For example, PM – T is the testing sample under thermal cycle, PM – 1 and PM – 2 are the samples fractured for microstructural analyses. Impedance data at 1, 7, and 28 days of curing are archived in the University of Bath Research Data Archive DOI: <https://doi.org/10.15125/BATH-01577>).

SI – 12.2. Development of percolation process through curing age


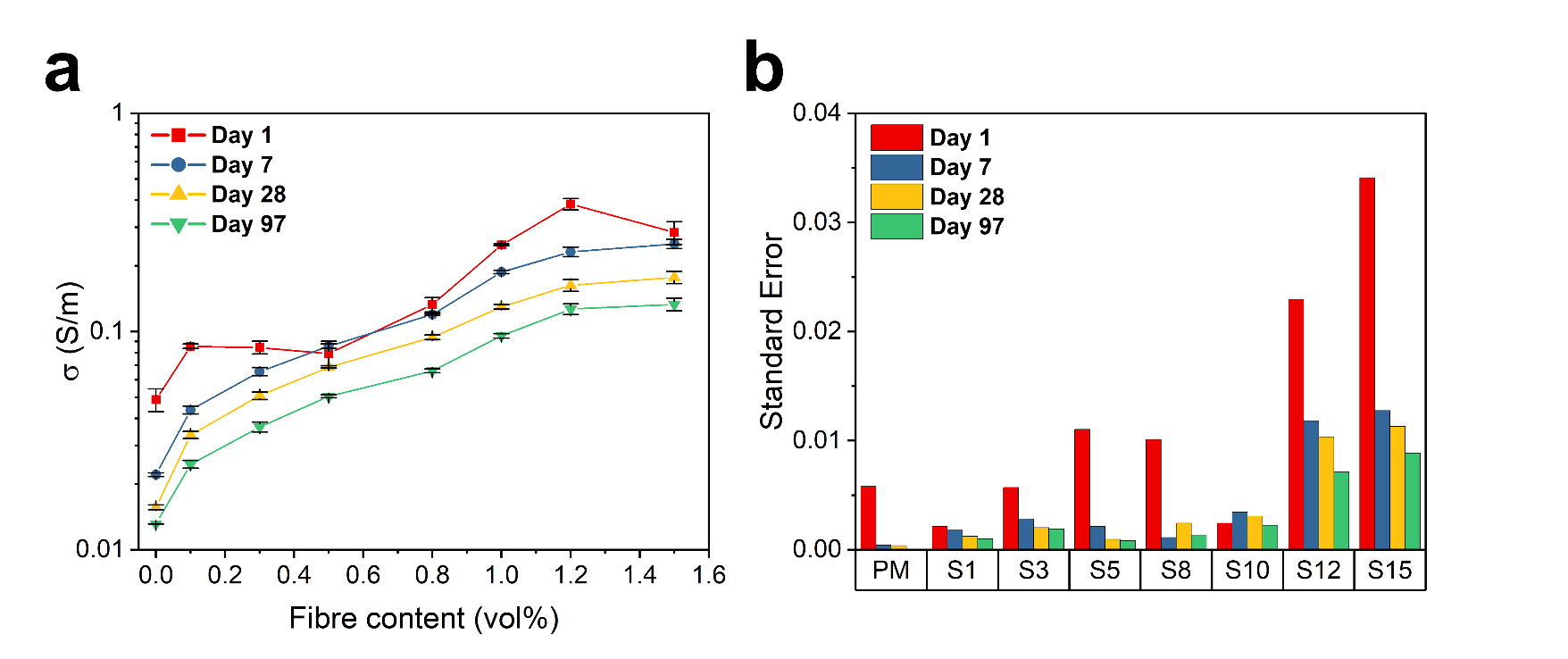


1. Development of percolation trend at the first day and the curing ages of 7, 28, and 97 days. a) Percolation process of carbon fibre in cement matrix from 0 to 1.5 vol% with standard error bars. b) Specific standard error values for the variation of electrical conductivity of the three samples from in the same fabrication batch.
2. Effect of temperature and fibre content on impedance behaviours

SI – 13.1. Complex impedance of PM and CEMe


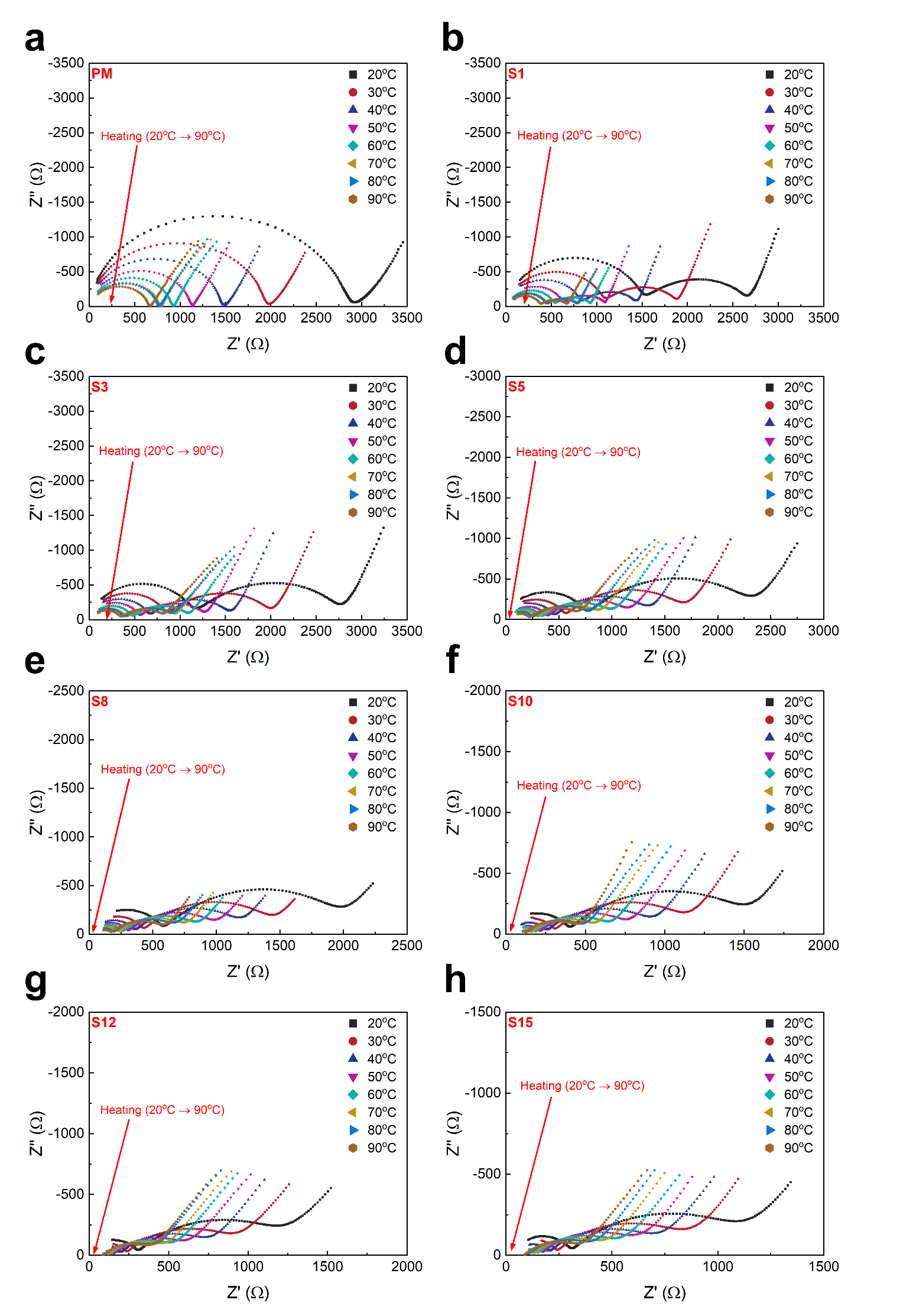


1. Development of impedance response of PM and CEMe with temperature during heating stage. a) PM. b) S1. c) S3. d) S5. e) S8. f) S10. g) S12. h) S15.


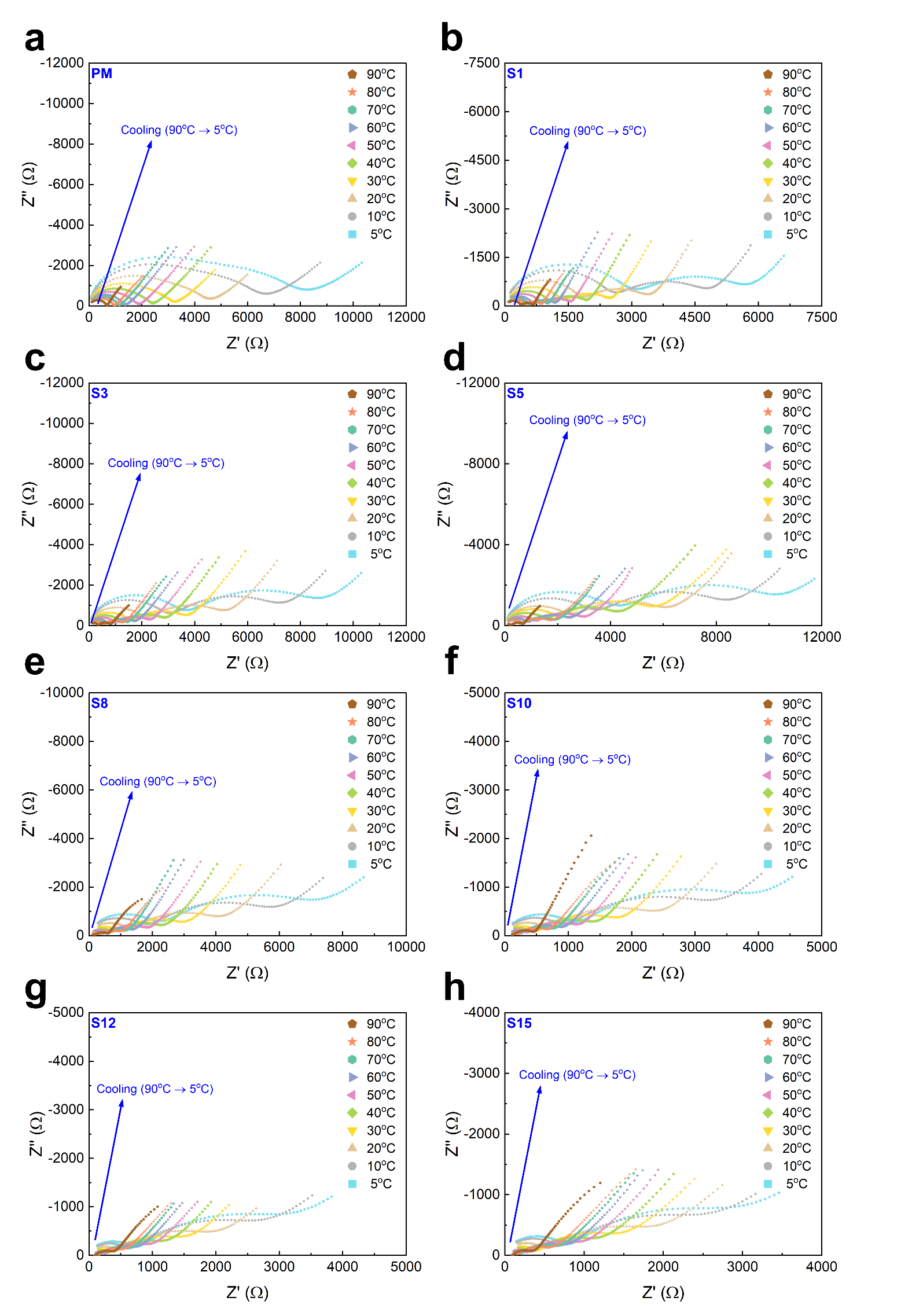


1. Development of impedance response of PM and CEMe with temperature during cooling stage. a) PM. b) S1. c) S3. d) S5. e) S8. f) S10. g) S12. h) S15.

SI – 13.2. Complex impedance of simulated pore solution


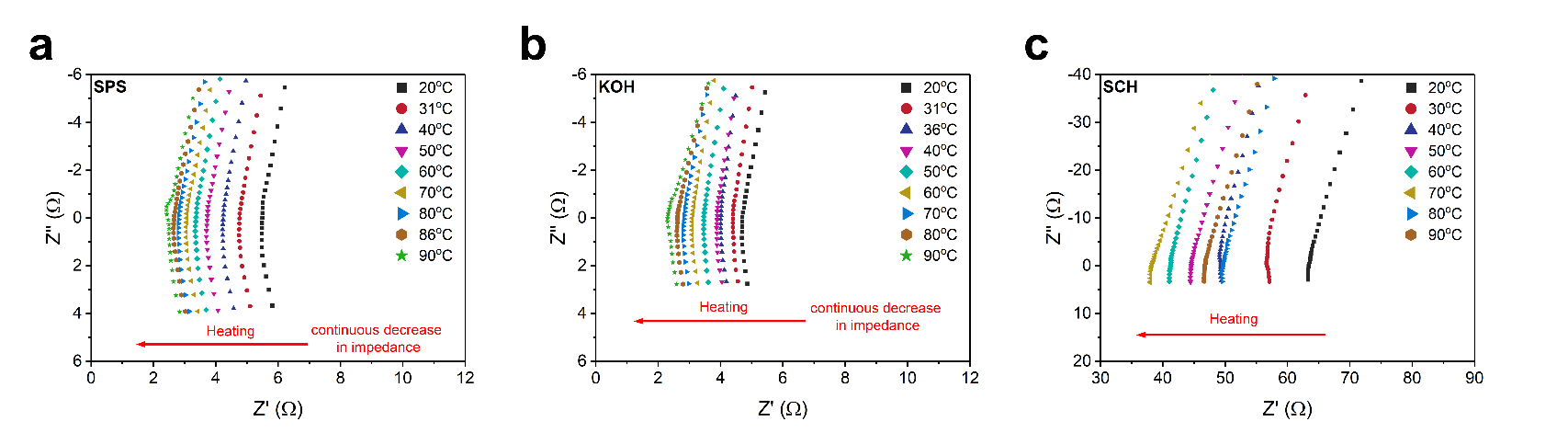


1. Development of impedance response of SPS, KOH, and SCH solutions during heating. a) SPS. b) KOH. c)SCH.


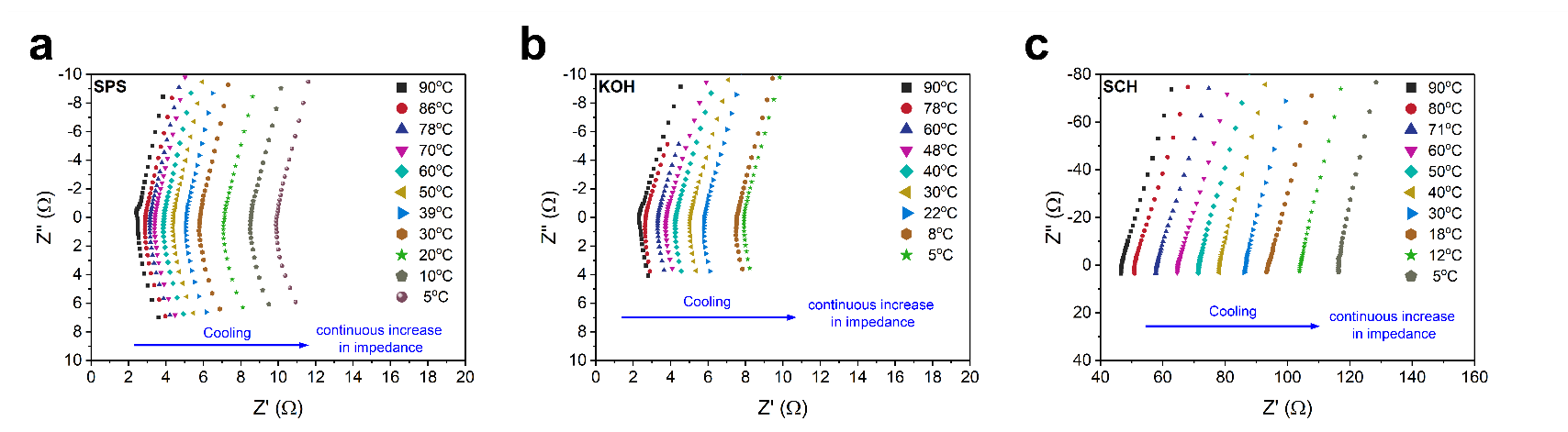


1. Development of impedance response of SPS, KOH, and SCH solutions during cooling. a) SPS. b) KOH. c)SCH.

SI – 13.3. Loss tangent


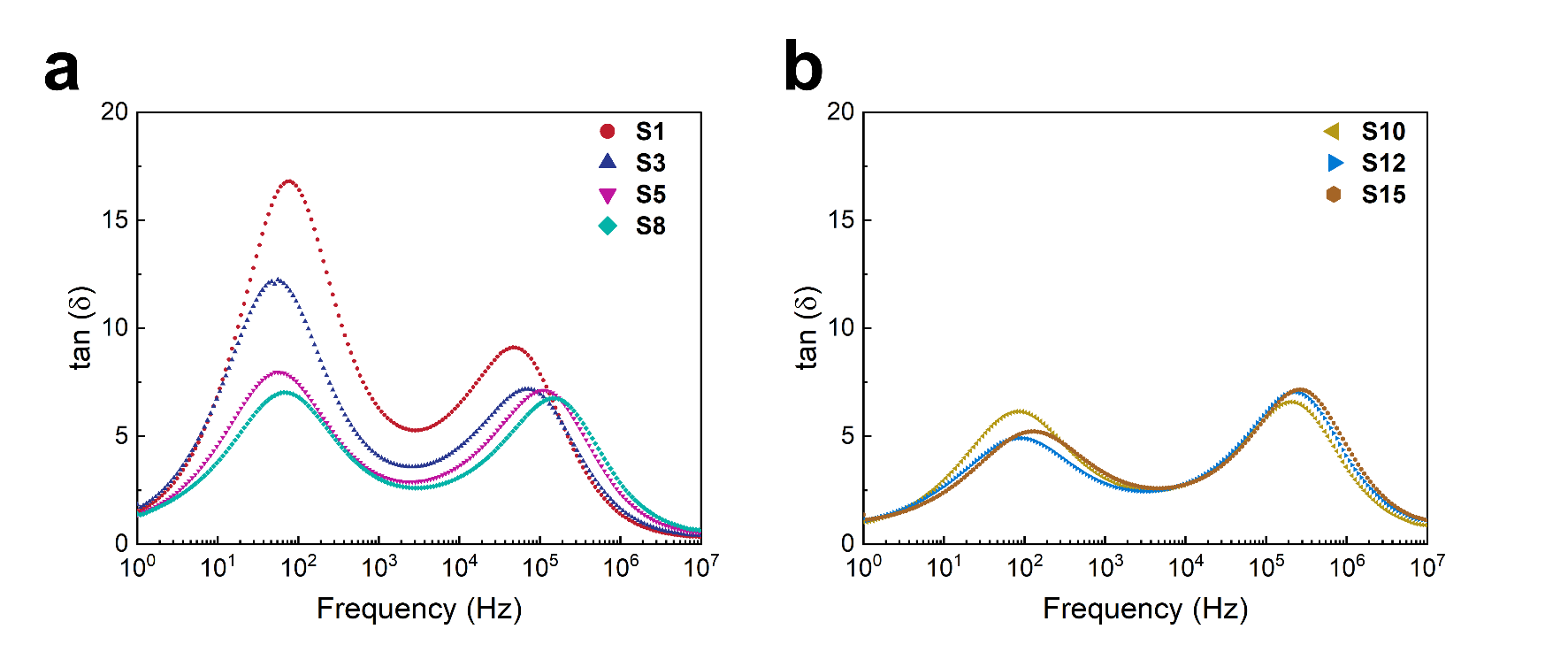


1. Comparison between low-freq peak tanδ and high-freq peak tanδ for CEMes with different fibre contents at pristine condition. a) CEMes with 0.1, 0.3, 0.5, and 0.8 vol% of carbon fibres showing consistent feature of low-freq peak tanδ > high-freq peak tanδ. b) CEMes with 1, 1.2, and 1.5 vol% of carbon fibres showing low-freq peak tanδ < high-freq peak tanδ.


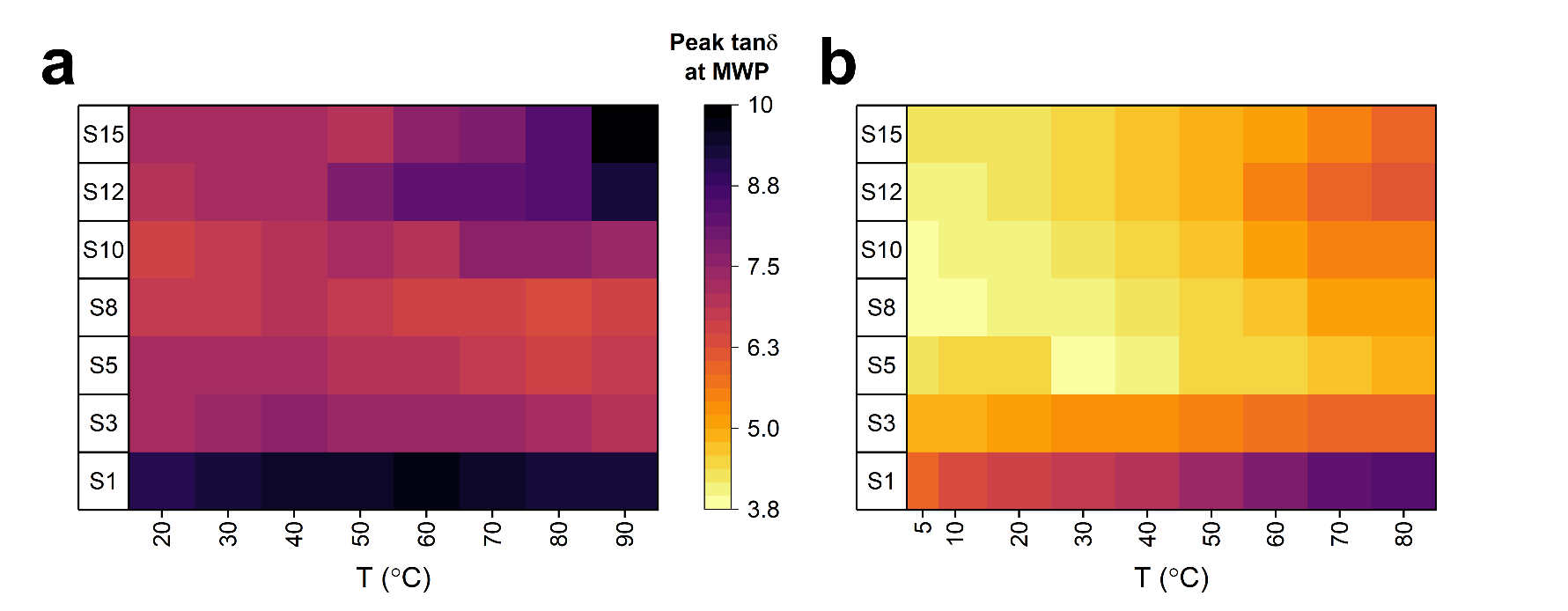


1. Heat map showing the dependency of high-freq peak tanδ at MWP on temperature and fibre content. a) at heating stage. b) at cooling stage.
2. Thermally induced alteration in the architecture of conduction pathways

SI – 14.1. Ionic conduction pathway (pore network)


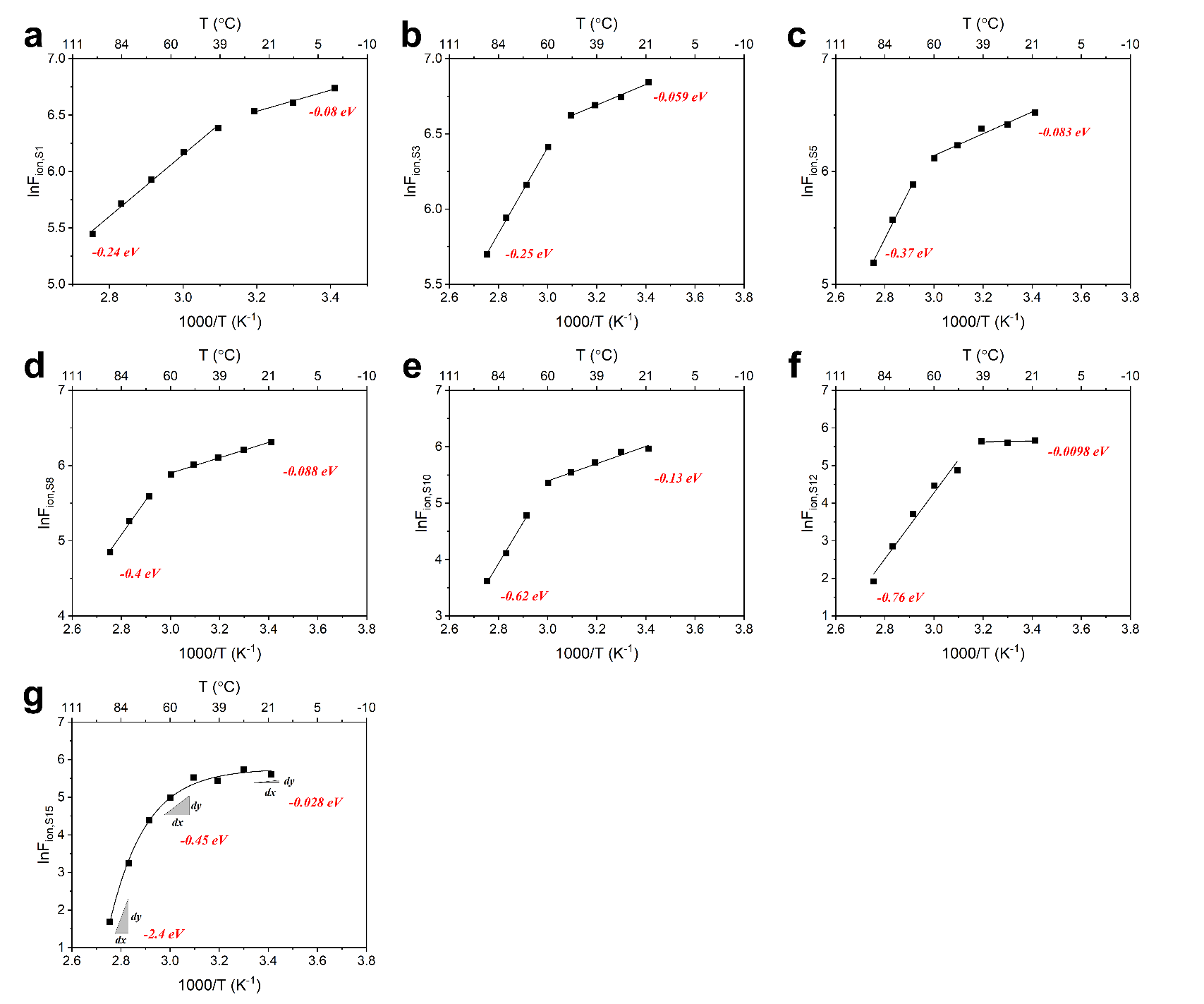


1. Formation factor of ionic conduction pathway $F_{ion}$ for CEMes during heating. a) S1. b) S3. c) S5. d) S8. e) S10. f) S12. g) S15.

SI – 14.2. Electronic conduction pathway (fibrous network)


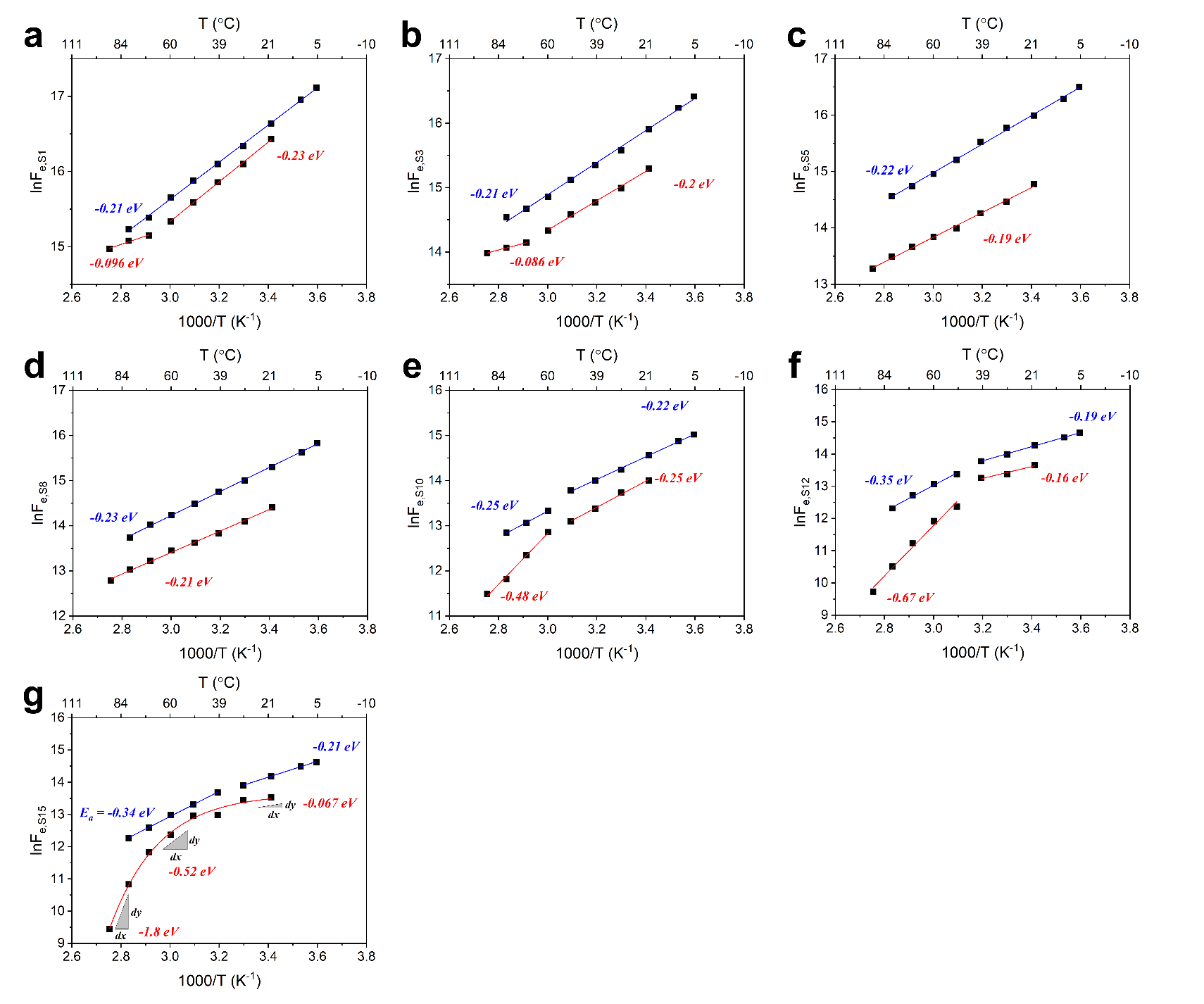


1. Formation factor of electronic conduction pathway $F_{e}$ for CEMes during heating (marked by red fitting line) and cooling (marked by blue fitting line). a) S1. b) S3. c) S5. d) S8. e) S10. f) S12. g) S15.

SI – 14.3. Materials degradation energy diagrams


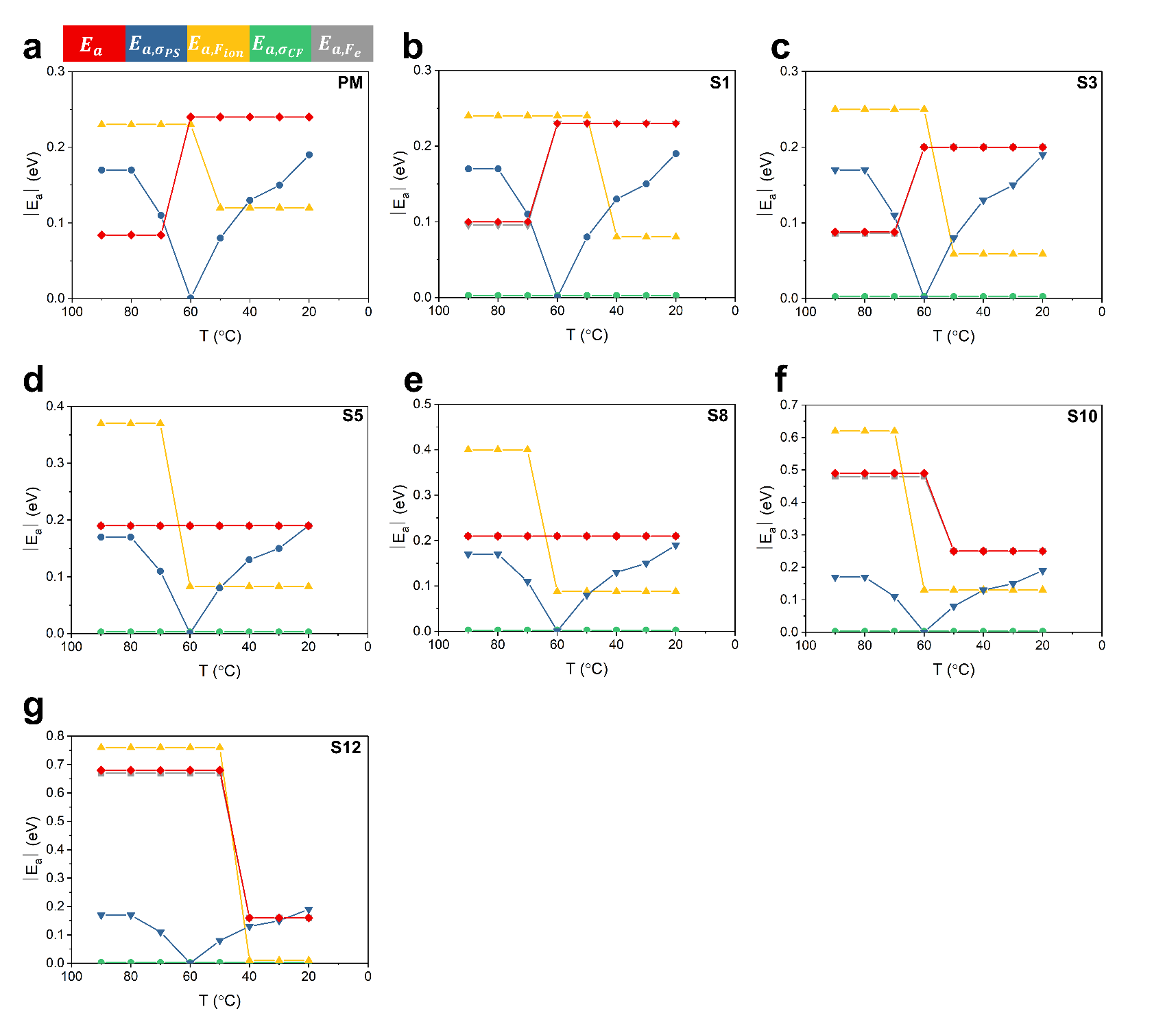


1. Energy diagrams. a) PM. b) S1. c) S3. d) S5. e) S8. f) S10. g) S12. Figure legends: $E_{a}$ the effective activation energy of bulk conductivity in red, $E_{a,\sigma_{PS}}$ the activation energy of intrinsic conductivity of pore solution in deep blue, $E_{a,F_{ion}}$ the activation energy for the “loosening” change of pore network in yellow , $E_{a,\sigma_{CF}}$ the activation energy of intrinsic conductivity of carbon fibre in green, $E_{a,F_{e}}$ the activation energy for “disconnecting” process of fibrous network in grey.
2. Determination on ionic and electronic conductivity percentages

A series of long-term drying process were conducted on samples prepared with the same experimental protocol for the determination of percentages of ionic and electronic conductivity with respect to overall bulk conductivity. Full set of drying data and experimental configurations have been uploaded to ***University of Bath Research Data Archive***. Available from: <https://doi.org/10.15125/BATH-01193>.

1. Solving process of environmental susceptibility $\boldsymbol{\chi}$

First of all, Steinhart-Hart formulation was employed to correlate normalized bulk resistance with temperature, which was a phenomenological approach and originally for calibrating thermistors. Comparing to linearized Arrhenius equation (Equation S18), Steinhart-Hart formulation uses resistance instead of conductivity, and it has an opposite positioning of temperature and electrical property with a polynomial manner hence a maximized fitting capability [67]:

| $\frac{1000}{T}=A_{s}+B_{s}\ln\left( \frac{R_{b}}{R_{0}} \right)+C_{s}\ln^{3} \left( \frac{R_{b}}{R_{0}} \right)$ | (S34) |
| --- | --- |

where $A_{s}$, $B_{s}$, and $C_{s}$ are the fitting coefficients, $T$ the temperature in K, and the dependent variable has been modified into $\frac{1000}{T}$ just to be consistent with Arrhenius approach in this study, $\frac{R_{b}}{R_{0}}$ the normalized bulk resistance with respect to the bulk conductivity at 20°C.

Secondly, $S_{\chi}$ was calculated through area segmentation, as presented in Figure 7d-1:

| $S_{h}=S_{\chi}+S_{c}+S_{top}+S_{bottom}$ | (S35) |
| --- | --- |

where $S_{h}$ is the area enclosed by the curve of heating (in red) and the horizontal axis, $S_{c}$ the area enclosed by the curve of cooling (in blue) and the horizontal axis, $S_{top}$ the area on the top by extending the fitting of heating until having the same $\frac{R_{b}}{R_{0}}$ as that of cooling, $S_{bottom}$ the area at the bottom. Note that this determination is only limited in a temperature range of 20 – 90°C both for heating and cooling. For the ease of calculation and further algorithm development, the $S_{\chi}$ was determined by solving the antiderivative of $d\left( \frac{R_{b}}{R_{0}} \right)$ with respect to horizontal axis. In Figure 7d-1, the antiderivatives of $d\left( \frac{R_{b}}{R_{0}} \right)$ of the heating and cooling curves have the expressions of:

| $S_{h}=\int_{\frac{R_{h,90}}{R_{0}}}^{\frac{R_{c, 20}}{R_{0}}} f_{h}\left( \frac{R_{b}}{R_{0}} \right)d\left( \frac{R_{b}}{R_{0}} \right)$ | (S36) |
| --- | --- |

for heating,

| $S_{c}=\int_{\frac{R_{c,90}}{R_{0}}}^{\frac{R_{c, 20}}{R_{0}}} f_{c}\left( \frac{R_{b}}{R_{0}} \right)d\left( \frac{R_{b}}{R_{0}} \right)$ | (S37) |
| --- | --- |

for cooling,

| $S_{top}=\int_{\frac{R_{h,20}}{R_{0}}}^{\frac{R_{c, 20}}{R_{0}}} f_{h}\left( \frac{R_{b}}{R_{0}} \right)d\left( \frac{R_{b}}{R_{0}} \right)-y_{20}\left( \frac{R_{c, 20}}{R_{0}}-\frac{R_{h, 20}}{R_{0}} \right)$ | (S38) |
| --- | --- |

for the area at the top, and finally

| $S_{bottom}=y_{90}\left( \frac{R_{c, 90}}{R_{0}}-\frac{R_{h, 90}}{R_{0}} \right)$ | (S39) |
| --- | --- |

for the area at the bottom, where $R_{0}$ is the resistance at 20°C pristine condition, $\frac{R_{c, 20}}{R_{0}}$ the normalized resistance of cooling at 20°C, $\frac{R_{c,90}}{R_{0}}$ the normalized resistance of cooling at 90°C (i.e., an algebraic result), $\frac{R_{h,20}}{R_{0}}$ the normalized resistance of heating at 20°C (i.e., the unity), $\frac{R_{h,90}}{R_{0}}$ the normalized resistance of heating at 90°C, $y_{20}$ the value of $\frac{1000}{T}$ at 20°C, $y_{90}$ the value of $\frac{1000}{T}$ at 90°C, and finally the $f_{h}\left( \frac{R_{b}}{R_{0}} \right)$ and $f_{c}\left( \frac{R_{b}}{R_{0}} \right)$ are the Steinhart-Hart fittings of heating and cooling, respectively. In Figure 7d-1, $f_{h,h90}$, $f_{c,c90}$, $f_{h,h20}$, $f_{c,c20}$, and $f_{h,c20}$ were abbreviated expressions of the boundary points above $\left[ f_{h}\left( \frac{R_{h, 90}}{R_{0}} \right),\frac{R_{h, 90}}{R_{0}} \right]$, $\left[ f_{c}\left( \frac{R_{c, 90}}{R_{0}} \right),\frac{R_{c, 90}}{R_{0}} \right]$, $\left[ f_{h}\left( \frac{R_{h, 20}}{R_{0}} \right),\frac{R_{h, 20}}{R_{0}} \right]$, $\left[ f_{c}\left( \frac{R_{c, 20}}{R_{0}} \right),\frac{R_{c, 20}}{R_{0}} \right]$, and $\left[ f_{h}\left( \frac{R_{c, 20}}{R_{0}} \right),\frac{R_{c, 20}}{R_{0}} \right]$, respectively.

Combining Equations S35, S36, S37, S38, and S39, ultimately, the environmental susceptibility $\chi$ between one cycle of heating and cooling for each sample obtains its general formulation as:

| $\chi=S_{\chi}=\frac{R_{c, 20}}{R_{0}}\left( C_{h}\left( \ln^{3} \frac{R_{c, 20}}{R_{0}}-3\ln^{2} \frac{R_{c, 20}}{R_{0}}+6\ln\frac{R_{c, 20}}{R_{0}}-6 \right)+B_{h}\left( \ln\frac{R_{c, 20}}{R_{0}}-1 \right)+A_{h} \right)-\frac{R_{h,90}}{R_{0}}\left( C_{h}\left( \ln^{3} \frac{R_{h,90}}{R_{0}}-3\ln^{2} \frac{R_{h,90}}{R_{0}}+6\ln\frac{R_{h,90}}{R_{0}}-6 \right)+B_{h}\left( \ln\frac{R_{h,90}}{R_{0}}-1 \right)+A_{h} \right)-\frac{R_{c, 20}}{R_{0}}\left( C_{c}\left( \ln^{3} \frac{R_{c, 20}}{R_{0}}-3\ln^{2} \frac{R_{c, 20}}{R_{0}}+6\ln\frac{R_{c, 20}}{R_{0}}-6 \right)+B_{c}\left( \ln\frac{R_{c, 20}}{R_{0}}-1 \right)+A_{c} \right)+\frac{R_{c,90}}{R_{0}}\left( C_{c}\left( \ln^{3} \frac{R_{c,90}}{R_{0}}-3\ln^{2} \frac{R_{c,90}}{R_{0}}+6\ln\frac{R_{c,90}}{R_{0}}-6 \right)+B_{c}\left( \ln\frac{R_{c,90}}{R_{0}}-1 \right)+A_{c} \right)-\frac{R_{c, 20}}{R_{0}}\left( C_{h}\left( \ln^{3} \frac{R_{c, 20}}{R_{0}}-3\ln^{2} \frac{R_{c, 20}}{R_{0}}+6\ln\frac{R_{c, 20}}{R_{0}}-6 \right)+B_{h}\left( \ln\frac{R_{c, 20}}{R_{0}}-1 \right)+A_{h} \right)+\frac{R_{h,20}}{R_{0}}\left( C_{h}\left( \ln^{3} \frac{R_{h,20}}{R_{0}}-3\ln^{2} \frac{R_{h,20}}{R_{0}}+6\ln\frac{R_{h,20}}{R_{0}}-6 \right)+B_{h}\left( \ln\frac{R_{h,20}}{R_{0}}-1 \right)+A_{h} \right)+\left( \frac{R_{c, 20}}{R_{0}}-\frac{R_{h, 20}}{R_{0}} \right)y_{20} -\left( \frac{R_{c, 90}}{R_{0}}-\frac{R_{h, 90}}{R_{0}} \right)y_{90}$ | (S40) |
| --- | --- |

where $A$, $B$, and $C$ with subscripts $h$ and $c$ refer to the fitting coefficients for heating and cooling curves, respectively.

Equation S40 can be further reduced into the final expression:

| $\chi=S_{\chi}=\frac{R_{c,90}}{R_{0}}\left( C_{c}\left( \ln^{3} \frac{R_{c,90}}{R_{0}}-3\ln^{2} \frac{R_{c,90}}{R_{0}}+6\ln\frac{R_{c,90}}{R_{0}}-6 \right)+B_{c}\left( \ln\frac{R_{c,90}}{R_{0}}-1 \right)+A_{c} \right)-\frac{R_{c, 20}}{R_{0}}\left( C_{c}\left( \ln^{3} \frac{R_{c, 20}}{R_{0}}-3\ln^{2} \frac{R_{c, 20}}{R_{0}}+6\ln\frac{R_{c, 20}}{R_{0}}-6 \right)+B_{c}\left( \ln\frac{R_{c, 20}}{R_{0}}-1 \right)+A_{c} \right)-\frac{R_{h,90}}{R_{0}}\left( C_{h}\left( \ln^{3} \frac{R_{h,90}}{R_{0}}-3\ln^{2} \frac{R_{h,90}}{R_{0}}+6\ln\frac{R_{h,90}}{R_{0}}-6 \right)+B_{h}\left( \ln\frac{R_{h,90}}{R_{0}}-1 \right)+A_{h} \right)+\left( \frac{R_{c, 20}}{R_{0}}-\frac{R_{h, 20}}{R_{0}} \right)y_{20}-\left( \frac{R_{c, 90}}{R_{0}}-\frac{R_{h, 90}}{R_{0}} \right)y_{90}+A_{h}-B_{h}-6C_{h}$ | (S41) |
| --- | --- |

Last but not least, considering the moisture state, the quantification of environmental susceptibility based on experimental data here is close to a realistic condition because variation of saturation degree for exposed structure is between 80 – 100% [10]. The deployment method for CEMes can be versatile: a functional array being distributed throughout concrete matrix, a covering layer (or coating), or a monolithic structural element, where the moisture variation can be reduced if deploying deeper into the structure.

This section provides an originally developed method for a solution of real-time quantifying and monitoring on the environmental susceptibility of CEMe. It is suggested that the logic of the algorithm for real-time monitoring can be a continuous automation with sequential steps as follows: step 1 the Steinhart-Hart fitting on heating and cooling cycles, step 2 the area segmentation method for identifying $S_{\chi}$, and finally step 3 is solving the antiderivatives by the embedment of Equation S41 in the automation algorithm. Further development should be automated correction of fitting error (i.e., from unexpected external stimuli such as chloride ingression, unexpected impact loads, etc.) under site trial when the above sequential steps iterate over time.

1. SI References

[1] M. Soutsos, P. Domone, eds., Construction Materials: Their Nature and Behaviour, 5th ed., CRC Press, Fifth edition. | Boca Raton : CRC Press, [2017], 2017. https://doi.org/10.1201/9781315164595.

[2] C. Hall, W.D. Hoff, Water transport in brick, stone and concrete, Third edition, CRC Press, Boca Raton London New York, 2022.

[3] D.P. Bentz, A virtual rapid chloride permeability test, Cement and Concrete Composites 29 (2007) 723–731. https://doi.org/10.1016/j.cemconcomp.2007.06.006.

[4] K.A. Snyder, X. Feng, B.D. Keen, T.O. Mason, Estimating the electrical conductivity of cement paste pore solutions from OH−, K+ and Na+ concentrations, Cement and Concrete Research 33 (2003) 793–798. https://doi.org/10.1016/S0008-8846(02)01068-2.

[5] National Institute of Standards and Technology (NIST), Estimation of Pore Solution Conductivity, Estimation of Pore Solution Conductivity (2019). https://www.nist.gov/el/estimation-pore-solution-conductivity.

[6] A. Moragues, A. Macias, C. Andrade, Equilibria of the chemical composition of the concrete pore solution. Part I: Comparative study of synthetic and extracted solutions, Cement and Concrete Research 17 (1987) 173–182. https://doi.org/10.1016/0008-8846(87)90100-1.

[7] A. Moragues, A. Macias, C. Andrade, J. Losada, Equilibria of the chemical composition of the pore concrete solution Part II: Calculation of the equilibria constants of the synthetic solutions, Cement and Concrete Research 18 (1988) 342–350. https://doi.org/10.1016/0008-8846(88)90068-3.

[8] L.G. Benning, D. Gebauer, M. Kellermeier, A.E.S. Van Driessche, eds., New Perspectives on Mineral Nucleation and Growth: From Solution Precursors to Solid Materials, 1st ed. 2017, Springer International Publishing : Imprint: Springer, Cham, 2017. https://doi.org/10.1007/978-3-319-45669-0.

[9] H.F.W. Taylor, A method for predicting alkazi ion concentrations in cement pore solutions, Advances in Cement Research 1 (1987) 5–17. https://doi.org/10.1680/adcr.1987.1.1.5.

[10] J. Bao, S. Li, P. Zhang, S. Xue, Y. Cui, T. Zhao, Influence of exposure environments and moisture content on water repellency of surface impregnation of cement-based materials, Journal of Materials Research and Technology 9 (2020) 12115–12125. https://doi.org/10.1016/j.jmrt.2020.08.046.

[11] J. Zhang, A. Heath, H.M.T. Abdalgadir, R.J. Ball, K. Paine, Electrical impedance behaviour of carbon fibre reinforced cement-based sensors at different moisture contents, Construction and Building Materials 353 (2022) 129049. https://doi.org/10.1016/j.conbuildmat.2022.129049.

[12] A. Khaleghi, S.M. Sadrameli, M. Manteghian, Thermodynamic and kinetics investigation of homogeneous and heterogeneous nucleation, Reviews in Inorganic Chemistry 40 (2020) 167–192. https://doi.org/10.1515/revic-2020-0004.

[13] J. Zhang, A. Heath, R.J. Ball, B. Chen, L. Tan, G. Li, J. Pan, T.B. Su-Cadirci, K. Paine, Piezoresistivity and piezopermittivity of cement-based sensors under quasi-static stress and changing moisture, Construction and Building Materials 425 (2024) 136052. https://doi.org/10.1016/j.conbuildmat.2024.136052.

[14] B.J. Christensen, T. Coverdale, R.A. Olson, S.J. Ford, E.J. Garboczi, H.M. Jennings, T.O. Mason, Impedance Spectroscopy of Hydrating Cement-Based Materials: Measurement, Interpretation, and Application, Journal of the American Ceramic Society 77 (1994) 2789–2804. https://doi.org/10.1111/j.1151-2916.1994.tb04507.x.

[15] D. Rothstein, J.J. Thomas, B.J. Christensen, H.M. Jennings, Solubility behavior of Ca-, S-, Al-, and Si-bearing solid phases in Portland cement pore solutions as a function of hydration time, Cement and Concrete Research 32 (2002) 1663–1671. https://doi.org/10.1016/S0008-8846(02)00855-4.

[16] R.H. Mills, Transportation Research Board (TRB), Factors influencing cessation of hydration in water cured cement pastes, Symposium on Structure of Portland Cement Paste and Concrete, Highway Research Board Special Report 90 (1966) 406–424.

[17] T. Knudsen, The dispersion model for hydration of portland cement I. General concepts, Cement and Concrete Research 14 (1984) 622–630. https://doi.org/10.1016/0008-8846(84)90024-3.

[18] D.P. Bentz, Three‐Dimensional Computer Simulation of Portland Cement Hydration and Microstructure Development, Journal of the American Ceramic Society 80 (1997) 3–21. https://doi.org/10.1111/j.1151-2916.1997.tb02785.x.

[19] F. Lin, C. Meyer, Hydration kinetics modeling of Portland cement considering the effects of curing temperature and applied pressure, Cement and Concrete Research 39 (2009) 255–265. https://doi.org/10.1016/j.cemconres.2009.01.014.

[20] L.A. Bromley, Thermodynamic properties of strong electrolytes in aqueous solutions, AIChE Journal 19 (1973) 313–320. https://doi.org/10.1002/aic.690190216.

[21] J.O. Bockris, A.K.N. Reddy, J.O. Bockris, Ionics, 2. ed, Plenum Press, New York, NY, 1998.

[22] S. Diamond, Long-term status of calcium hydroxide saturation of pore solutions in hardened cements, Cement and Concrete Research 5 (1975) 607–616. https://doi.org/10.1016/0008-8846(75)90061-7.

[23] W. Rechenberg, S. Sprung, Composition of the solution in the hydration of cement, Cement and Concrete Research 13 (1983) 119–126. https://doi.org/10.1016/0008-8846(83)90135-7.

[24] S.A. Greenberg, L.E. Copeland, THE THERMODYNAMIC FUNCTIONS FOR THE SOLUTION OF CALCIUM HYDROXIDE IN WATER, J. Phys. Chem. 64 (1960) 1057–1059. https://doi.org/10.1021/j100837a023.

[25] B. Lothenbach, Thermodynamic equilibrium calculations in cementitious systems, Mater Struct 43 (2010) 1413–1433. https://doi.org/10.1617/s11527-010-9592-x.

[26] K. Szyszkiewicz-Warzecha, G. Wilczek-Vera, A. Lewenstam, A. Górska, J. Tarasiuk, R. Filipek, The Influence of Chemical Activity Models on the Description of Ion Transport through Micro-Structured Cementitious Materials, Materials (Basel) 16 (2023) 1116. https://doi.org/10.3390/ma16031116.

[27] W. Ma, P.W. Brown, D. Shi, Solubility of Ca(OH)2 and CaSO4·2H2O in the liquid phase from hardened cement paste, Cement and Concrete Research 22 (1992) 531–540. https://doi.org/10.1016/0008-8846(92)90003-E.

[28] M.R. Wright, An introduction to aqueous electrolyte solutions, John Wiley & sons, Chichester, 2007.

[29] F. Schmidt, F.S. Rostásy, A method for the calculation of the chemical composition of the concrete pore solution, Cement and Concrete Research 23 (1993) 1159–1168. https://doi.org/10.1016/0008-8846(93)90176-A.

[30] B. Lothenbach, D.A. Kulik, T. Matschei, M. Balonis, L. Baquerizo, B. Dilnesa, G.D. Miron, R.J. Myers, Cemdata18: A chemical thermodynamic database for hydrated Portland cements and alkali-activated materials, Cement and Concrete Research 115 (2019) 472–506. https://doi.org/10.1016/j.cemconres.2018.04.018.

[31] R.J. Ball, G.C. Allen, G. Starrs, W.J. McCarter, Impedance spectroscopy measurements to study physio-chemical processes in lime-based composites, Appl. Phys. A 105 (2011) 739–751. https://doi.org/10.1007/s00339-011-6509-7.

[32] X. Ke, S.A. Bernal, J.L. Provis, B. Lothenbach, Thermodynamic modelling of phase evolution in alkali-activated slag cements exposed to carbon dioxide, Cement and Concrete Research 136 (2020) 106158. https://doi.org/10.1016/j.cemconres.2020.106158.

[33] J.R. Cooper, R12-08: Release on the IAPWS Formulation 2008 for the Viscosity of Ordinary Water Substance, (2008). https://iapws.org/documents/release/viscosity.

[34] M.L. Huber, R.A. Perkins, A. Laesecke, D.G. Friend, J.V. Sengers, M.J. Assael, I.N. Metaxa, E. Vogel, R. Mareš, K. Miyagawa, New International Formulation for the Viscosity of H2O, Journal of Physical and Chemical Reference Data 38 (2009) 101–125. https://doi.org/10.1063/1.3088050.

[35] R.B. McCleskey, D.K. Nordstrom, J.N. Ryan, Comparison of electrical conductivity calculation methods for natural waters, Limnology and Oceanography: Methods 10 (2012) 952–967. https://doi.org/10.4319/lom.2012.10.952.

[36] H. Bloom, E. Heymann, A.C.D. Rivett, The electric conductivity and the activation energy of ionic migration of molten salts and their mixtures, Proceedings of the Royal Society of London. Series A. Mathematical and Physical Sciences 188 (1997) 392–414. https://doi.org/10.1098/rspa.1947.0016.

[37] H. Schmidt, M. Wiebe, B. Dittes, M. Grundmann, Meyer-Neldel rule in ZnO, Applied Physics Letters 91 (2007) 232110. https://doi.org/10.1063/1.2819603.

[38] Survey and interpretation of pre-exponentials of conductivity, Solid State Ionics 28–30 (1988) 89–94. https://doi.org/10.1016/S0167-2738(88)80013-4.

[39] N. Takamure, X. Sun, T. Nagata, A. Ho-Baillie, N. Fukata, D.R. McKenzie, Thermodynamic Interpretation of the Meyer-Neldel Rule Explains Temperature Dependence of Ion Diffusion in Silicate Glass, Phys. Rev. Lett. 129 (2022) 175901. https://doi.org/10.1103/PhysRevLett.129.175901.

[40] A. Yildiz, F. Iacomi, M. Cazacu, A. Amironesei, G. i. Rusu, S. Simon, The Meyer-Neldel rule in layered silicone-silver nanocomposites, Polymer Composites 32 (2011) 1751–1756. https://doi.org/10.1002/pc.21204.

[41] P. Du, N. Li, X. Ling, Z. Fan, A. Braun, W. Yang, Q. Chen, A. Yelon, Optimizing the Proton Conductivity with the Isokinetic Temperature in Perovskite‐Type Proton Conductors According to Meyer–Neldel Rule, Advanced Energy Materials 12 (2022) 2102939. https://doi.org/10.1002/aenm.202102939.

[42] C.E. Salmas, G.P. Androutsopoulos, A Novel Pore Structure Tortuosity Concept Based on Nitrogen Sorption Hysteresis Data, Ind. Eng. Chem. Res. 40 (2001) 721–730. https://doi.org/10.1021/ie000626y.

[43] Q. Zeng, M. Luo, X. Pang, L. Li, K. Li, Surface fractal dimension: An indicator to characterize the microstructure of cement-based porous materials, Applied Surface Science 282 (2013) 302–307. https://doi.org/10.1016/j.apsusc.2013.05.123.

[44] D.N. Winslow, The fractal nature of the surface of cement paste, Cement and Concrete Research 15 (1985) 817–824. https://doi.org/10.1016/0008-8846(85)90148-6.

[45] W.J. McCarter, The fractal surface of cementitious materials determined by impedance spectroscopy, Advances in Cement Research 6 (1994) 147–154. https://doi.org/10.1680/adcr.1994.6.24.147.

[46] A.J. Katz, A.H. Thompson, Fractal Sandstone Pores: Implications for Conductivity and Pore Formation, Phys. Rev. Lett. 54 (1985) 1325–1328. https://doi.org/10.1103/PhysRevLett.54.1325.

[47] P. Wong, Fractal surfaces in porous media, in: AIP Conference Proceedings, AIP, 1987: pp. 304–317. https://doi.org/10.1063/1.36383.

[48] J. Cai, W. Wei, X. Hu, D.A. Wood, Electrical conductivity models in saturated porous media: A review, Earth-Science Reviews 171 (2017) 419–433. https://doi.org/10.1016/j.earscirev.2017.06.013.

[49] S.W. Coleman, J.C. Vassilicos, Tortuosity of unsaturated porous fractal materials, Phys. Rev. E 78 (2008) 016308. https://doi.org/10.1103/PhysRevE.78.016308.

[50] P. Pfeifer, D. Avnir, Chemistry in noninteger dimensions between two and three. I. Fractal theory of heterogeneous surfaces, The Journal of Chemical Physics 79 (1983) 3558–3565. https://doi.org/10.1063/1.446210.

[51] B.B. Mandelbrot, The fractal geometry of nature, W.H. Freeman, San Francisco, 1982.

[52] B. Zhang, S. Li, Determination of the Surface Fractal Dimension for Porous Media by Mercury Porosimetry, Ind. Eng. Chem. Res. 34 (1995) 1383–1386. https://doi.org/10.1021/ie00043a044.

[53] E.M. Anitas, Small-Angle Scattering from Fractional Brownian Surfaces, Symmetry 13 (2021) 2042. https://doi.org/10.3390/sym13112042.

[54] K.J. Falconer, Fractal geometry: mathematical foundations and applications, Third edition, John Wiley & Sons Inc, Hoboken, 2014.

[55] M. Borkovec, W. De Paris, R. Peikert, THE FRACTAL DIMENSION OF THE APOLLONIAN SPHERE PACKING, Fractals 02 (1994) 521–526. https://doi.org/10.1142/S0218348X94000739.

[56] D.V. Stäger, H.J. Herrmann, CUTTING SELF-SIMILAR SPACE-FILLING SPHERE PACKINGS, Fractals 26 (2018) 1850013. https://doi.org/10.1142/S0218348X18500135.

[57] R. Dickau, Triangular Koch Fractal Surface, Wolfram Demonstrations Project (2011). https://demonstrations.wolfram.com/TriangularKochFractalSurface/ (accessed September 2, 2024).

[58] H.-O. Peitgen, H. Jürgens, D. Saupe, Chaos and fractals: new frontiers of science, 2. ed, Springer, New York Berlin Heidelberg, 2004.

[59] X. Han, B. Wang, J. Feng, Relationship between fractal feature and compressive strength of concrete based on MIP, Construction and Building Materials 322 (2022) 126504. https://doi.org/10.1016/j.conbuildmat.2022.126504.

[60] G. Barbera, G. Barone, V. Crupi, F. Longo, G. Maisano, D. Majolino, P. Mazzoleni, S. Raneri, J. Teixeira, V. Venuti, A multi-technique approach for the determination of the porous structure of building stone, Ejm 26 (2014) 189–198. https://doi.org/10.1127/0935-1221/2014/0026-2355.

[61] Y. Xie, D.M. Artymowicz, P.P. Lopes, A. Aiello, D. Wang, J.L. Hart, E. Anber, M.L. Taheri, H. Zhuang, R.C. Newman, K. Sieradzki, A percolation theory for designing corrosion-resistant alloys, Nat. Mater. 20 (2021) 789–793. https://doi.org/10.1038/s41563-021-00920-9.

[62] M.A. Klatt, S. Winter, Geometric functionals of fractal percolation, Adv. Appl. Probab. 52 (2020) 1085–1126. https://doi.org/10.1017/apr.2020.33.

[63] Y.M. Strelniker, S. Havlin, A. Bunde, Fractals and Percolation, in: R.A. Meyers (Ed.), Encyclopedia of Complexity and Systems Science, Springer New York, New York, NY, 2009: pp. 3847–3858. https://doi.org/10.1007/978-0-387-30440-3_227.

[64] Z. Casar, A.K. Mohamed, P. Bowen, K. Scrivener, Atomic-Level and Surface Structure of Calcium Silicate Hydrate Nanofoils, J. Phys. Chem. C 127 (2023) 18652–18661. https://doi.org/10.1021/acs.jpcc.3c03350.

[65] X. Ding, X. Liang, Y. Zhang, Y. Fang, J. Zhou, T. Kang, Capillary Water Absorption and Micro Pore Connectivity of Concrete with Fractal Analysis, Crystals 10 (2020) 892. https://doi.org/10.3390/cryst10100892.

[66] W. Chen, Y. Wang, D. Wang, Y. Liu, J. Liu, Predicting the effective thermal conductivity of porous building materials using improved Menger sponge fractal structure, International Journal of Thermal Sciences 184 (2023) 107985. https://doi.org/10.1016/j.ijthermalsci.2022.107985.

[67] J.S. Steinhart, S.R. Hart, Calibration curves for thermistors, Deep Sea Research and Oceanographic Abstracts 15 (1968) 497–503. https://doi.org/10.1016/0011-7471(68)90057-0.
